# Copper(ii) ketimides in sp^3^ C–H amination†

**DOI:** 10.1039/d1sc01990b

**Published:** 2021-11-05

**Authors:** Isuri U. Jayasooriya, Abolghasem (Gus) Bakhoda, Rachel Palmer, Kristi Ng, Nour L. Khachemoune, Jeffery A. Bertke, Timothy H. Warren

**Affiliations:** Department of Chemistry, Georgetown University Box 571227-1227 Washington DC 20057 USA warre155@msu.edu

## Abstract

Commercially available benzophenone imine (HN=CPh_2_) reacts with β-diketiminato copper(ii) *tert*-butoxide complexes [Cu^II^]–O^*t*^Bu to form isolable copper(ii) ketimides [Cu^II^]–N=CPh_2_. Structural characterization of the three coordinate copper(ii) ketimide [Me_3_NN]Cu–N=CPh_2_ reveals a short Cu-N_ketimide_ distance (1.700(2) Å) with a nearly linear Cu–N–C linkage (178.9(2)°). Copper(ii) ketimides [Cu^II^]–N=CPh_2_ readily capture alkyl radicals R˙ (PhCH(˙)Me and Cy˙) to form the corresponding R–N=CPh_2_ products in a process that competes with N–N coupling of copper(ii) ketimides [Cu^II^]–N=CPh_2_ to form the azine Ph_2_C=N–N=CPh_2_. Copper(ii) ketimides [Cu^II^]–N=CAr_2_ serve as intermediates in catalytic sp^3^ C–H amination of substrates R–H with ketimines HN=CAr_2_ and ^*t*^BuOO^*t*^Bu as oxidant to form *N*-alkyl ketimines R–N=CAr_2_. This protocol enables the use of unactivated sp^3^ C–H bonds to give R–N=CAr_2_ products easily converted to primary amines R–NH_2_*via* simple acidic deprotection.

## Abstract 2

Commercially available benzophenone imine (HN=CPh_2_) reacts with β-diketiminato copper(ii) *tert*-butoxide complexes [Cu^II^]–O^*t*^Bu to form isolable copper(ii) ketimides [Cu^II^]–N=CPh_2_ that serve as intermediates in catalytic sp^3^ C−H amination *via* radical relay.

## Graphical abstract

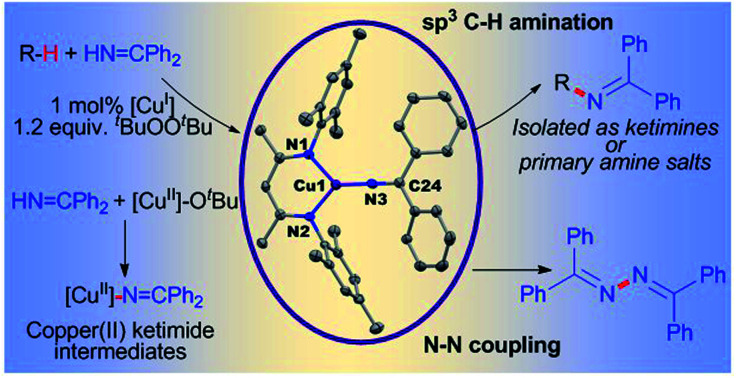

## Introduction

Transition metal-catalysed sp^3^ C–H amination protocols have gained immense attention in the synthetic community over the past couple of decades.^1–4^ A majority of these protocols proceed *via* metal–nitrene^2,5^ [M]=NR′ or metal–amide [M]–NR′R′′ intermediates.^1,6^ Extensive studies on such intermediates and underlying mechanisms have paved the way towards more efficient sp^3^ C–H amination protocols.^1^

**Fig. 1:**
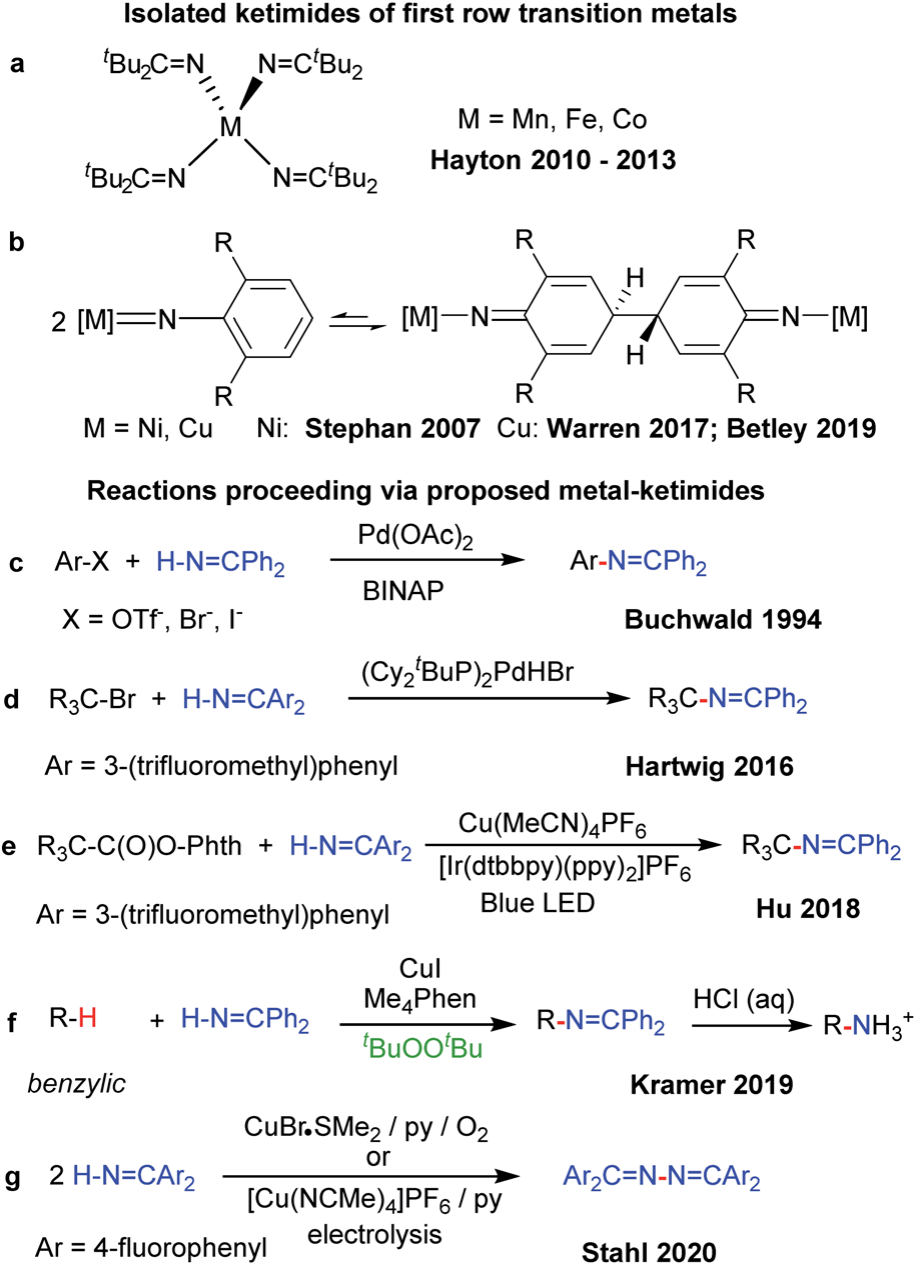
Transition metal–ketimide complexes.

Related metal–ketimide [M]–N=CR′R′′ intermediates, however, have received less attention in C–H amination chemistry. The strong metal–N_ketimide_ interaction makes ketimides effective spectator ligands. For instance, ketimides stabilize high valent homoleptic Mn(iv),^7^ Fe(iv)^8^ and Co(iv)^9^ complexes (Fig. 1a). In some cases, ketimides can also form *via* nickel and copper arylimido/nitrene intermediates [M]=NAr *via* C–C coupling at the *para*-position of the aryl nitrene ligand (Fig. 1b). While this reactivity was initially uncovered with nickel β-diketiminato complexes,^10^ reversible C–C bond formation/cleavage in related copper complexes provides access to terminal copper nitrenes [Cu]=NAr that participate in sp^3^ C–H amination.^11,12^

Fewer examples of ketimides exist, however, in which the ketimide ligand serves as a reactive functional group in discrete transition metal complexes.^13^ Metal ketimide intermediates have been proposed in several Pd-catalysed cross-coupling reactions of aryl (Fig. 1c)^14^ and alkyl halides (Fig. 1d)^15^ with benzophenone imine. Cu-catalysed photoredox cross-coupling reactions of redox-active alkyl esters (Fig. 1e)^16^ and Cu-catalysed benzylic sp^3^ C–H amination with benzophenone imine (Fig. 1f)^17^ are among other examples that may be mediated by metal–ketimide intermediates. Moreover, Stahl and colleagues have proposed copper(ii) ketimides in the N–N oxidative coupling of imines Ar_2_C=NH to azines Ar_2_C=N–N=CAr_2_ under aerobic or electrocatalytic conditions (Fig. 1g).^18,19^

**Fig. 2:**
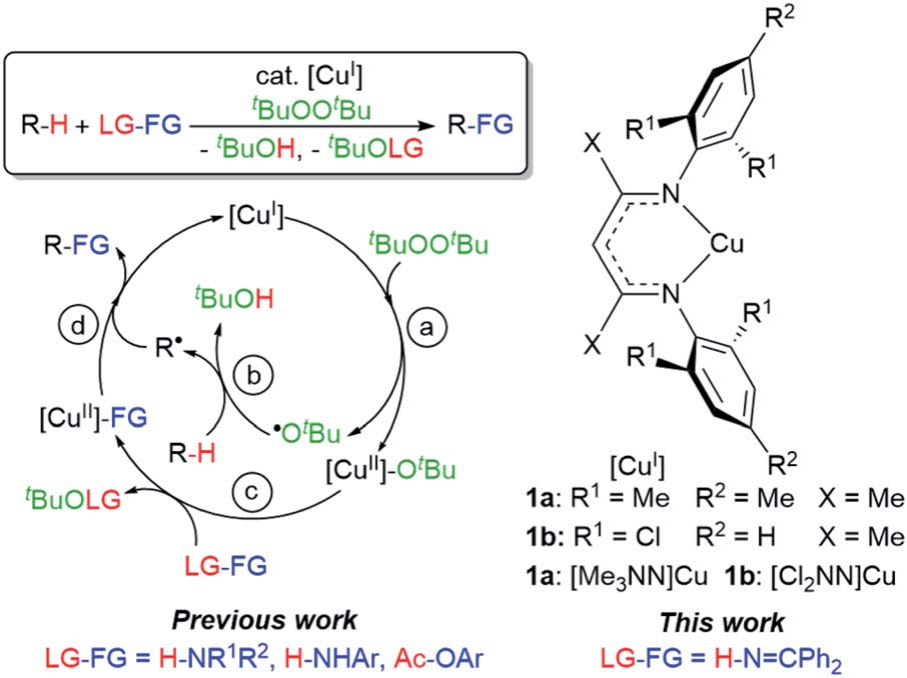
Mechanism of C–H functionalisation *via* β-diketiminato copper(ii) intermediates [Cu^II^]–FG.

Herein we describe discrete first-row transition metal–ketimide complexes intimately involved in C–H amination chemistry. Building upon the Kharasch–Sosnovsky reaction,^20–22^ we previously demonstrated that copper(ii) alkyl amides [Cu^II^]–NHR′,^23^ anilides [Cu^II^]–NHAr,^6,24^ and aryloxides [Cu^II^]–OAr^25^ serve as key intermediates in a radical relay protocol for sp^3^ C–H functionalisation (Fig. 2). Formed *via* acid–base^6,23,24^ or transesterification^25^ reactions between [Cu^II^]–O^*t*^Bu with H-FG or Ac-FG reagents, these copper(ii) complexes [Cu^II^]–FG capture sp^3^-C radicals R˙ generated *via* H-atom abstraction from R–H to furnish the functionalized product R-FG. We anticipated that the relatively high acidity of the imine N–H bond^26^ coupled with a preference for binding at copper with softer N-donors should enable the formation of [Cu^II^]–N=CAr_2_ species from [Cu^II^]–O^*t*^Bu complexes and HN=CPh_2_ allow for an examination of copper(ii) ketimides in C–H amination catalysis.

## Results and discussion

### Synthesis and characterization of copper(ii) ketimides

**Fig. 3:**
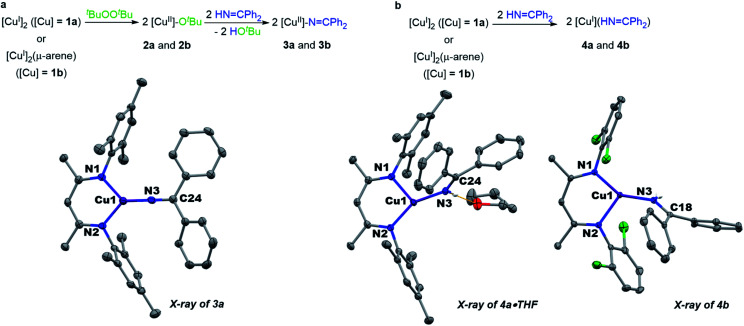
(a) Synthesis and structure of copper(ii) ketimides. (b) Synthesis and structure of copper(i) imine adducts.

Monitored by UV-vis spectroscopy, addition of benzophenone imine (1 equiv.) to a solution of [Me_3_NN]Cu-O^*t*^Bu (2a) in toluene at −80 °C results in decay of the characteristic UV-vis absorption of 2a at 470 nm with growth of a new band at 570 nm (Fig. S2†). Performed on a preparative scale, this new species [Me_3_NN]Cu–N=CPh_2_ (3a) may be isolated as dark purple crystals from pentane at −35 °C in 78% yield (Fig. 3a).

The X-ray crystal structure of [Me_3_NN]Cu–N=CPh_2_ (3a) (Fig. 3a) reveals the Cu–N_ketimide_ distance of 1.700(2) Å, significantly shorter than the Cu–N bond found in the copper(ii) amide [Cl_2_NN]Cu–NHAd (1.839(9) Å)^23^ and copper(ii) anilide [Cl_2_NN]Cu–NHAr^Cl^_3_ (1.847(3) Å).^6^ Copper(ii) ketimide 3a possesses a nearly linear Cu–N3–C24 angle of 178.9(2)°. The short Cu–N_ketimide_ distance and linear Cu–N3–C24 angle support effective sp hybridization at the ketimide N atom. These values remarkably differ from those in the homoleptic copper(i) ketimide [Cu–N=CPh_2_]_4_ with bridging ketimide ligands that lead to a square-like tetrameric structure with Cu–N distances 1.847(2)–1.861(2) Å and Cu–N–Cu angles of 94.17(9)–98.25(9).^27^ To outline differences between coordination of anionic ketimide ligands and their neutral ketimine counterparts, we prepared the corresponding benzophenone imine adducts [Me_3_NN]Cu(NH=CPh_2_) (4a) and [Cl_2_NN]Cu(NH=CPh_2_) (4b) (Fig. 3b). These copper(i) complexes feature substantially longer Cu–N_ketimine_ distances of 1.8940(14) and 1.8937(14) Å. These ketimine adducts 4a and 4b each exhibit a pronounced bend in the Cu–ketimide linkage with Cu–N–C angles of 132.68(12) and 130.25(12)° consistent with sp^2^ hybridization at N.

UV-vis analysis of copper(ii) ketimide [Me_3_NN]Cu–N=CPh_2_ (3a) reveals the presence of a single low energy absorption band at 570 nm (*ε* = 1910 M^−1^ cm^−1^) in toluene at room temperature. The EPR spectrum of 3a in a mixture of toluene and pentane at room temperature shows a signal centred at *g*_iso_ = 2.081 with very well resolved coupling to ^63/65^Cu (*A*_Cu_ = 298.0 MHz) and additional hyperfine modelled with three equivalent ^14^N nuclei (*A*_N_ = 35.0 MHz) (Fig. S13†). The related copper(ii) ketimide [Cl_2_NN]Cu–N=CPh_2_ (3b) prepared from [Cl_2_NN]Cu-O^*t*^Bu (2b) and HN=CPh_2_ exhibits a similar spectroscopic profile. The UV-vis spectrum of [Cl_2_NN]Cu–N=Ph_2_ (3b) exhibits a single absorption at 520 nm (*ε* = 3120 M^−1^ cm^−1^) in toluene at room temperature and possesses a similar isotropic EPR spectrum to that of 3a (Fig. S14†). Unfortunately, the greater thermal sensitivity of [Cl_2_NN]Cu–N=Ph_2_ (3b) has precluded its crystallographic characterization.

**Fig. 4:**
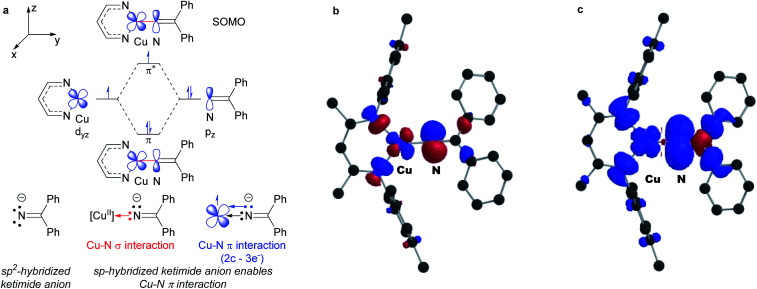
(a) Electronic structure of copper(ii) ketimides. (b) SOMO and (c) spin density plot of copper(ii) ketamide 3a (net spin α: blue, net spin β: red, 0.001 isospin value).

DFT calculations reveal remarkably high unpaired electron density on the ketimide N atom of both 3a (0.58) and 3b (0.61) (Fig. 4 and S23†). These values are significantly higher than values reported for related three coordinate β-diketiminato Cu(ii) anilides [Cu^II^]–NHAr (0.23–0.25)^6^ and a copper(ii) amide [Cu^II^]–NHAd (0.49).^23^ We rationalize this as a result of a 2-center 3-electron π interaction between the highest energy d orbital at the copper(ii) center destabilized by the β-diketiminato N-donors and a p orbital of the sp-hybridized ketimide N atom (Fig. 4a). In addition, the orthogonal orientation of the Cu–N_ketimide_ π-interaction relative to the conjugated ketimide N=CPh_2_ π system further limits the delocalization of unpaired electron density away from the ketimide N atom (Fig. 4b and c).

### Copper(ii) ketimide reactivity: radical capture and N–N bond formation

**Fig. 5:**
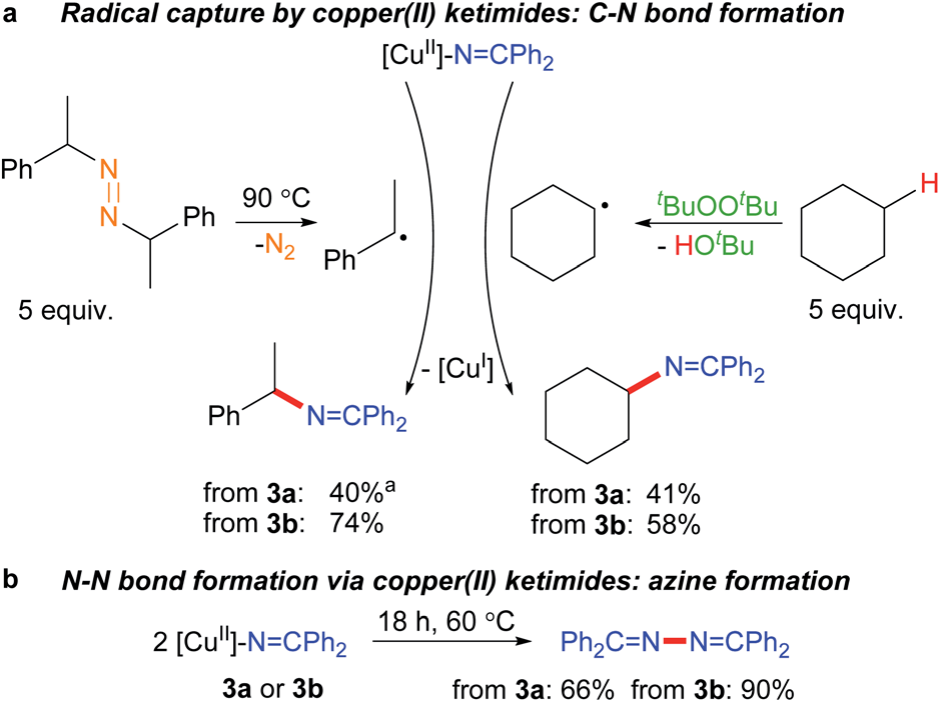
Reactivity of copper(ii) ketimides. ^*a*^ 2 equiv. diazene radical precursor.

The ability of many β-diketiminato copper(ii) complexes to participate in catalytic sp^3^ C–H functionalisation *via* radical relay (Fig. 2) encouraged us to assess the reactivity of copper(ii) ketimides 3 towards alkyl radicals. We find that [Cu^II^]–N=CPh_2_ species 3a and 3b capture alkyl radicals R˙ to provide the corresponding R–N=CPh_2_ products (Fig. 5a). [Cu^I^] is anticipated to form in these radical capture reactions that correspond to step d in the radical relay catalytic cycle (Fig. 2). For instance, reaction of 3a and 3b with (*E*/*Z*)-azobis(α-phenylethane) at 90 °C that generates the benzylic radical PhCH(˙)Me upon heating provides the alkylated imine PhCH(N=CPh_2_)Me in 40% and 74% yields, respectively. Generation of Cy˙ radicals in the presence of 3a and 3b by heating ^*t*^BuOO^*t*^Bu in cyclohexane (*via* H-atom abstraction by ^*t*^BuO˙ radicals) provides Cy-N=CPh_2_ in 58% and 41% yields, respectively.

Upon heating to 60 °C, copper(ii) ketimides 3a and 3b undergo N–N coupling to form benzophenone azine Ph_2_C=N–N=CPh_2_ isolated in 66% and 90% yields, respectively (Fig. 5b). This represents a competing reaction for radical capture at copper(ii) ketimides 3a and 3b.

### Copper(ii) ketimides in sp^3^ C–H amination

With a fundamental understanding of copper(ii) ketimide formation and reactivity, we explored these complexes in catalytic C–H amination *via* radical relay. Using ethylbenzene as a model R–H substrate, we screened a modest range of copper(i) β-diketiminato catalysts 1 that possess different electronic and steric properties (Table 1). The catalyst [Cl_2_NN]Cu (1b) provides the highest yield compared to more electron-rich (1a and 1c) and electron-poor (1d) catalysts. Increasing the ^*t*^BuOO^*t*^Bu oxidant amount does not significantly improve the yield. Lowering the temperature from 90 °C reduces the yield drastically (Table S1†), possibly due to binding of the ketimine HN=CAr_2_ to the copper(i) catalyst (Fig. 3b) that inhibits ^*t*^BuOO^*t*^Bu activation.^28^

**Table 1:** Copper catalysed C–H amination of ethylbenzene with benzophenone imine^a^

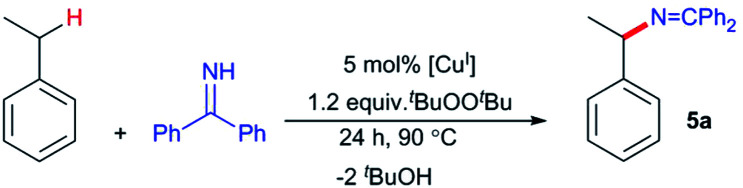			
Entry	Catalyst	(X, R^1^, R^2^)	Yield (%)
1	[Me_3_NN]Cu 1a	(Me, Me, Me)	34
2	[Cl_2_NN]Cu 1b	(Me, Cl, H)	65
3	[^*t*^Pr_2_NN]Cu 1c	(Me, ^*t*^Pr, H)	30
4	[Cl_2_NN_F6_]Cu 1d	(CF_3_, Cl, H)	42

a Conditions: 50 equiv. R–H. All yields determined by ^1^H NMR

While (1-(*tert*-butoxy)ethyl)benzene forms in trace amounts *via* C–H etherification,^28^ the azine Ph_2_C=N–N=CPh_2_ is the main byproduct in these catalytic C–H amination reactions, representing non-productive consumption of H–N=CPh_2_. In a previous study of C–H amination with anilines H_2_NAr employing the [Cl_2_NN]Cu/^*t*^BuOO^*t*^Bu catalyst system, electron-poor anilines provided the highest yields in the face of competing diazene ArN=NAr formation.^24^ Copper(ii) anilido intermediates [Cu^II^]–NHAr serve as intermediates in C–H amination with anilines H_2_NAr; those derived from electron-poor anilines H_2_NAr (*e.g.* Ar = 2,4,6-Cl_3_C_6_H_2_) proved more resistant to reductive bimolecular N–N bond formation.^6,24^

To examine whether similar electronic changes in the ketimine H–N=CAr_2_ could similarly promote more efficient catalysis, we explored two electron-poor ketimine derivatives H–N=CAr_2_ (Ar = 4-CF_3_C_6_H_4_ and 4-FC_6_H_4_) in C–H amination (Table 2). Although the *p*-CF_3_ substituted imine provides a higher C–H amination yield with cyclohexane (C–H BDE = 97 kcal mol^−1^),^29^ the increase in yield is modest with the benzylic substrate ethylbenzene (C–H BDE = 87 kcal mol^−1^).^29^ No significant differences were observed between benzophenone imine and the *p*-F substituted analogue.

**Table 2:** Copper catalysed C–H amination with benzophenone imine derivatives^a^

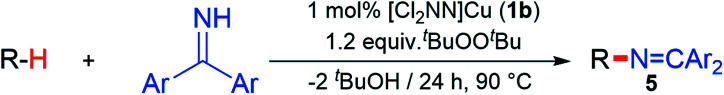			
Entry	Ar	Yield (%)	
		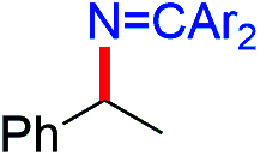	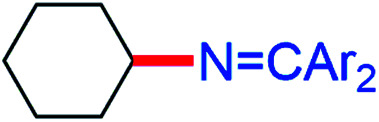
1	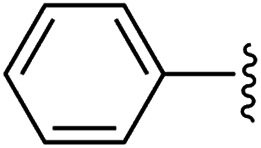	44 (5a)	40 (5b)
2	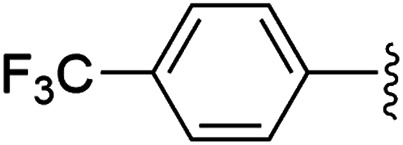	51 (5a-CF_3_)	56 (5b-CF_3_)
3	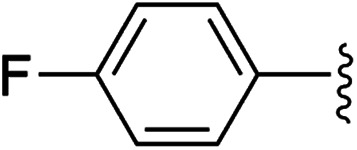	36 (5a-F)	39 (5b-F)

a Conditions: 10 equiv. R–H, 1.2 equiv. ^*t*^BuOO^*t*^Bu, 1 mol% [Cl_2_NN]Cu, 90 °C, 24 h. Yields are determined by ^1^H NMR.

While electron-poor imines can give somewhat higher C–H amination yields, we most broadly examined the commercially available H–N=CPh_2_ to survey the scope of R–H substrates in sp^3^ C–H amination (Table 3). Ethers such as THF, 1,4-dioxane, or even 12-crown-4 undergo C–H amination at the α-carbon in relatively high yields (6a–6d). Amination of the benzylic secondary C–H bonds in heteroaromatic substrates occurs (6f–6g), though yields may be lower due to the possibility of coordination of these substrates and/or products to the copper(i) centre that can decrease the rate of reoxidation with ^*t*^BuOO^*t*^Bu.^28^ Aromatic substrates with benzylic C–H bonds undergo C–H amination in moderate to high yields (6h–6k). Cycloalkanes with stronger, unactivated sp^3^ C–H bonds give moderate yields with electron-poor ketimine HN=CAr′_2_ (Ar′ = 4-CF_3_C_6_H_4_) (6l–6o). The bicyclic eucalyptol undergoes C–H amination in 32% yield (6e). These aminated products may be isolated either as synthetically versatile protected primary amines R–N=CPh_2_*via* column chromatography (6a–6g) or as the primary ammonium salts [R–NH_3_]Cl *via* deprotection upon simple acidic work up (6h–6o) under mild conditions. The potential to use recovered benzophenone from deprotection of ketimine products and azine byproducts to regenerate the Ph_2_C=NH starting material^30^ enhances the overall atom economy of this amination protocol.

**Table 3:** Copper catalyzed sp^3^ C–H amination with ketimines HN=CAr_2_^a^

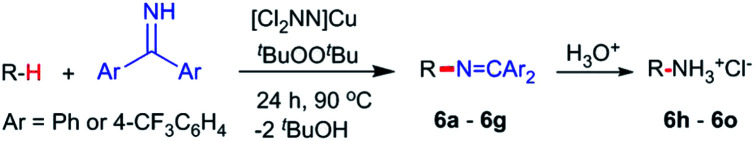
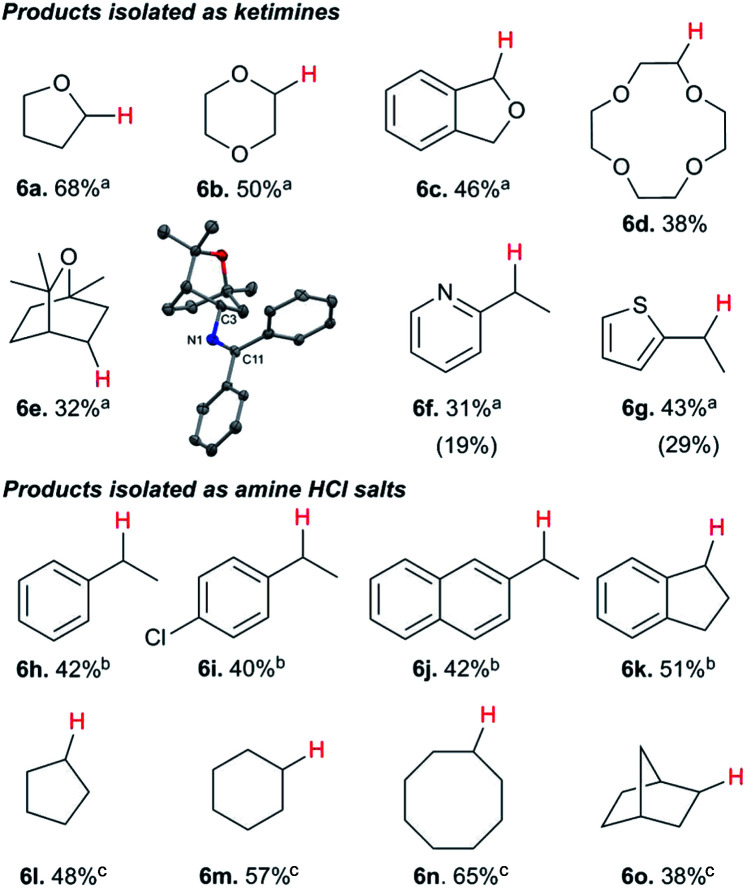

a Conditions: 10 equiv. R–H, 1.2 equiv. ^*t*^BuOO^*t*^Bu, 1 mol% [Cl_2_NN]Cu, 90 °C, 24 h.

b Yields with HN=CPh_2_.

c Yields with HN=CAr′_2_ (Ar′ = 4-CF_3_C_6_H_4_). ^1^H NMR yields (isolated yields) for 6f and 6g.

## Conclusions

The isolation of mononuclear copper(ii) ketimides [Cu^II^]–N=CPh_2_ reveals the role that they play as intermediates in sp^3^ C–H amination. These reactive intermediates readily form *via* acid–base exchange between [Cu^II^]–O^*t*^Bu and HN=CPh_2_, amenable to spectroscopic and structural investigation. Importantly, [Cu^II^]–N=CPh_2_ complexes efficiently intercept alkyl radicals R˙ generated *via* H-atom abstraction by ^*t*^BuO˙ from substrates R–H that ultimately enable the C–H amination of unactivated sp^3^ C–H substrates. DFT analysis reveals a significant amount of unpaired electron density at the ketimide N atom of 0.58 and 0.61 e^−^ for [Me_3_NN]Cu–N=CPh_2_ (3a) and [Cl_2_NN]Cu–N=CPh_2_ (3b) (Fig. 4 and S23†), respectively, opening a facile pathway for C–N bond formation with radicals R˙ to form R–N=CPh_2_ products (Fig. 5a). Moreover, this spin density at the ketimide N-atom likely facilitates N–N bond formation *via* copper(ii) ketimides [Cu^II^]–N=CPh_2_ to give the azine Ph_2_C=N–N=CPh_2_ (Fig. 5b), a competing pathway in sp^3^ C–H functionalisation. Use of the more electron-poor ketimine HN=CAr′ (Ar′ = 4-CF_3_C_6_H_4_) extends the scope of catalysis to unactivated sp^3^ C–H bonds in cycloalkanes (Table 3; entries 6l–6o). Nonetheless, facile N–N bond formation also by copper(ii) ketimides [Cu^II^]–N=CAr_2_ underscores the role that they may play in the (electro)catalytic copper(ii) promoted oxidative N–N coupling of benzophenone imine to form benzophenone azine (Fig. 1g).^18^

## Experimental section

Detailed experimental procedures are provided in the ESI.†

## Data availability

All synthetic procedures, characterization data, spectroscopic data, computational data, supplementary figures and tables, and detailed crystallographic information can be found in the ESI.† Crystallographic data are available *via* the Cambridge Crystallographic Data Centre (CCDC): 1940417, 1945374, 1940418, 1945375, 1940420, 2035780.

## Author contributions

I. U. J. and A. B. prepared and characterized the metal complexes, I. U. J. performed reactivity and computational studies with metal complexes, I. U. J., R. P., K. N., and N. L. K. carried out catalytic amination experiments, isolating and characterizing organic products, J. A. B. solved and refined X-ray diffraction data, T. H. W. guided the research and assisted with data analysis, I. U. J. and T. H. W. wrote the manuscript with input from all authors.

## Conflicts of interest

There are no conflicts to declare.

## Supplementary Material

SC-012-D1SC01990B-s001

SC-012-D1SC01990B-s002
