# Supplementary material for: Copper(ii) ketimides in sp3 C–H amination
Source: Chem Sci. 2021 Nov 5;12(47):15733–8. doi: 10.1039/d1sc01990b (PMC8654034; doi:10.1039/d1sc01990b)
Supplement: SC-012-D1SC01990B-s001 [file SC-012-D1SC01990B-s001.pdf]

Electronic Supplementary Material (ESI) for Chemical Science.
This journal is © The Royal Society of Chemistry 2021

Supporting Information for

Copper(II) Ketimides in sp^3 C-H Amination

Isuri U. Jayasooriya,[a] Abolghasem (Gus) Bakhoda,[a,b] Rachel Palmer,[a] Kristi Ng,[a] Nour L. Khachmoune,[a] Jeffery Bertke,[a] and Timothy H. Warren[a,c,]*

[a]Department of Chemistry, Georgetown University, Box 571227-1227, Washington, DC 20057 United States

[b]Current Address: Department of Chemistry, Towson University, 8000 York Road, Towson, MD 21252, United States

[c]Current Address: Department of Chemistry, Michigan State University, 578 S. Shaw Lane, East Lansing, MI 48824, United States

Table of Contents for Supporting Information

1. **General Procedures and Instrumentation**
2. **Synthesis, Characterization and Reactivity of Cu Complexes**
3. **Synthesis of Benzophenone Imine Derivatives**
4. **Catalytic C-H Amination**
5. **EPR Spectra of Cu(II) Intermediates**
6. **Crystallographic Data**
7. **References for Synthetic and Crystallographic Details**
8. **Computational Methods and Results**
9. **NMR Spectra**

1. General Procedures and Instrumentation

All catalytic reactions were carried out in a nitrogen-filled glovebox. 4A molecular sieves were activated *in vacuo* at 180 ºC for 24 h. Celite was dried overnight at 200 ºC under vacuum. Extra dry solvents (≥99.5%) with Acroseal® and deuterated solvents were purchased from Acros organics and Cambridge Isotope Laboratories, respectively. Both anhydrous and deuterated solvents were sparged with nitrogen and stored over activated 4A molecular sieves under a nitrogen atmosphere. 1,4-dioxane, 1,3-dihydrobenzofuran, 12-crown-4, eucalyptol, 2-ethylpyridine, ethylbenzene, 4-chloroethylbenzene, indane, 2-ethylnaphthalene, cyclohexane, cyclopentane, cyclooctane, norbornane were purchased from Sigma and TCI and sparged with nitrogen and stored over 4A molecular sieves before use. All solvents were tested before use with a drop of sodium benzophenone ketyl in THF solution.

Benzophenone imine (97%) was purchased from Acros, purged with nitrogen and stored over 4A molecular sieves prior to use. *para*-substituted benzonitriles, aryl halides and Grignard reagents for benzophenone imine derivative synthesis were purchased from Sigma and used as received. (*E/Z*)-azobis(α-phenylethane),[1] {[Cl_2NN]Cu}$_2$(μ-benzene),[2] {[Me_3NN]Cu}$_2$(μ-toluene),[3] [^{i}Pr$_2$NN]Cu(NCMe)[4] {[Cl_2NN_{F6}]Cu}$_2$(μ-toluene)[5] and [Cl_2NN]Cu-O^{t}Bu[6] were prepared according to literature reported methods. {[Cl_2NN]Cu}$_2$(μ-benzene) may be obtained from Strem Chemicals.

^{1}H, ^{13}C{H} and ^{19}F NMR spectra were recorded on a 400 MHz Varian Spectrometer (400, 100.47 and 376 MHz respectively). All NMR spectra were recorded at room temperature unless otherwise noted. ^{1}H NMR and ^{13}C{H} NMR spectra were indirectly referenced to tetramethylsilane using residual solvent signal as the internal standard. ^{19}F NMR spectra were recorded in presence of an internal reference of fluorobenzene (δ = −113.15 ppm vs. $CFCl_3$ δ =0.00) or (trifluormethyl)trimethylislane (δ = -67.3 ppm vs. $CFCl_3$ δ =0.00).

Elemental analyses were performed on a Perkin−Elmer PE2400 microanalyzer at Georgetown University. UV−vis spectra were recorded on Agilent 8454 Diode Array spectrometer equipped with stirrer and Unisoku USP−203 cryostat for variable temperature (−105 °C to 90 °C) experiments. The molar extinction coefficients of different isolated complexes were determined from Beer's law plots (absorbance vs concentration) with at least

four different concentrations. High resolution mass data were collected at mass spectrometry facility at Indiana University.

EPR spectra were collected on a JEOL continuous wave spectrometer JES-FA200 equipped with an X-band Gunn oscillator bridge, a cylindrical mode cavity, and a liquid nitrogen cryostat. EPR measurements were performed in sealed quartz tubes. All spectra were obtained on freshly prepared solutions (1-3 mM in toluene) of at least 2 independently synthesized batches and were checked carefully for reproducibility. Background spectra were obtained on clean solvents at the same measurement conditions. Spectral simulation was performed using the program QCMP 136 by Prof. Dr. Frank Neese from the Quantum Chemistry Program Exchange as used by Neese *et al.* in *J. Am. Chem. Soc.* **1996**, *118*, 8692-8699. The fittings were performed by the "chi by eye" approach and always taking all available spectra at different temperatures into account.

2. Synthesis, Characterization and Reactivity of Cu Intermediates

Synthesis of $[Me_3NN]Cu\text{-}O^tBu$ (2a).

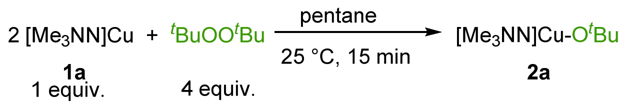

Scheme S1. Synthesis of $[Me_3NN]Cu\text{-}O^tBu$ (**2a**).

In a nitrogen filled glovebox, to a stirring solution of $\{[Me_3NN]Cu\}_2$(*μ*-toluene) (104 mg, 0.117 mmol) in pentane (*ca.* 15 mL) $^tBuOO^tBu$ (81.2 μL, 0.450 mmol, 4 equiv.) was added. After stirring for 15 minutes, the solution was filtered through a syringe filter and volatiles were removed *in vacuo* to get a reddish-brown oil in 94% yield. While usually isolated as an oil, a few X-ray quality crystals could be obtained from toluene at -37 °C. This intermediate was further characterized with UV-visible spectroscopy (Figure S1), EPR spectroscopy (Figure S14), and X-ray crystallography (Figure S17).

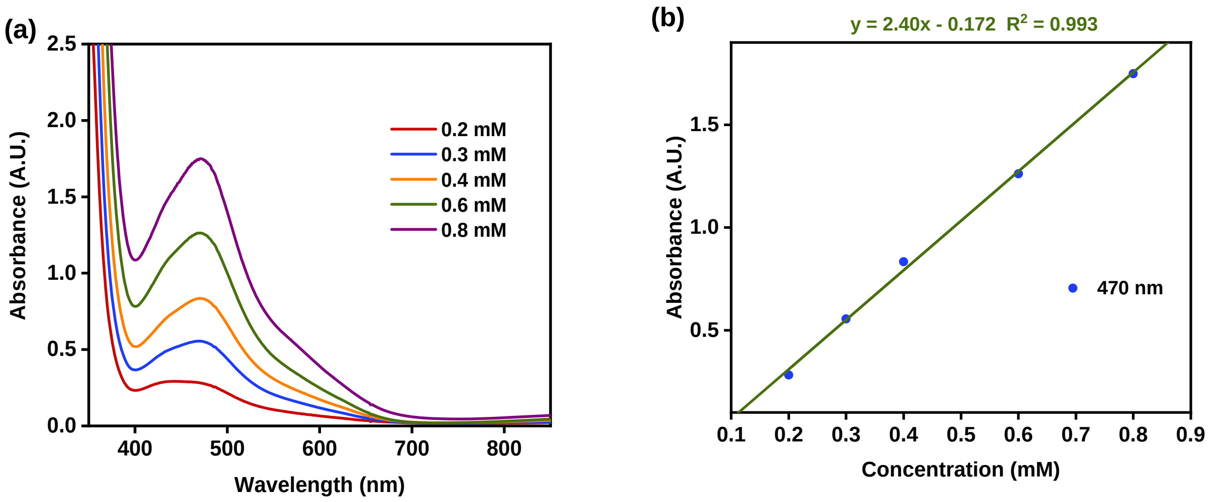

Figure S1. (a) UV-vis spectra of $[Me_3NNCu]\text{-}O^tBu$ (**2a**) in toluene at 25 °C at different concentrations (0.2 mM, 0.3 mM, 0.4 mM, 0.6 mM, 0.8 mM). (b) Beer's law plot for $[Me_3NNCu]\text{-}O^tBu$ (**2a**) depicts λ_{max} = 470 nm (ε = 2400 $M^{-1}cm^{-1}$).

Reaction of [Me_3NN]Cu-O^{t}Bu (2a) with benzophenone imine

In a nitrogen filled glovebox, a stock solution of [Me_3NN]Cu-O^{t}Bu (**2a**) was prepared by dissolving [Me_3NN]Cu-O^{t}Bu (68 mg, 0.140 mmol) in toluene and diluting up to 10.00 mL (13.5 mM). 148.0 μL of this solution was diluted up to 5.00 mL with toluene to obtain a 0.4 mM solution of [Me_3NN]Cu-O^{t}Bu. 3.50 mL of this solution was transferred into a cuvette. A stock solution of benzophenone imine was prepared by dissolving benzophenone imine (47.0 μL, 0.280 mmol) in toluene and diluting up to 10.00 mL (28.0 mM). Upon addition of 0.50 mL of the benzophenone imine stock solution (10 equiv.) to the cuvette, the growth of the absorbance at 570 nm was observed at -60 °C over the course of 2 h.

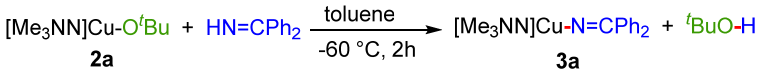

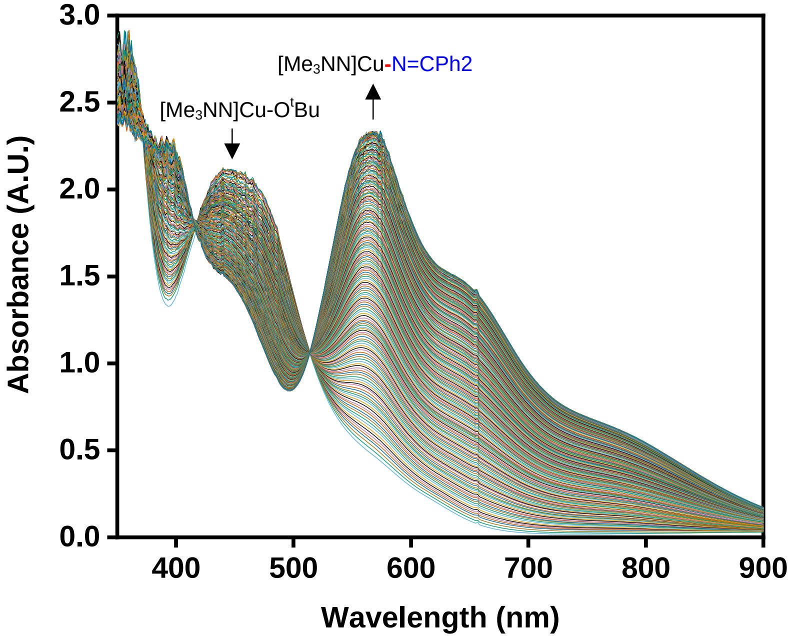

Figure S2. UV-vis spectra recorded in every 2 s during exchange reaction of [Me_3NN]Cu-O^{t}Bu (**2a**) (0.35 mM) and benzophenone imine (10 equiv.) in toluene at -60 °C over 2 h.

Synthesis of [Me_3NN]Cu-N=CPh_2 (3a).

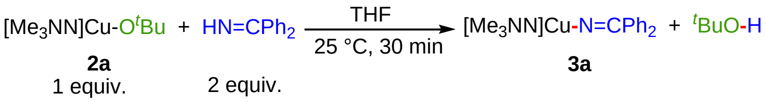

Scheme S2. Synthesis of [Me_3NN]Cu-N=CPh_2 (**3a**).

To a stirring solution of [Me_3NN]Cu-O^{t}Bu (**2a)** (118 mg, 0.230 mmol, 1 equiv.) in THF (8 mL) was added benzophenone imine (84.0 μL, 0.450 mmol, 2 equiv.). After stirring for 30 minutes, the solution was filtered through a pipette of Celite *ca.* 2 cm and volatiles were evaporated to provide the purple solid [Me_3NN]Cu-N=CPh_2 (**3a**) in 96% yield. Recrystallization from pentane afforded X-ray diffraction quality crystals in 78% yield. This species was further characterized with UV-vis spectroscopy (Figure S3), EPR spectroscopy (Figure S15), and X-ray crystallography (Figure S18). Due to high thermal sensitivity, this species could not be characterized by elemental analysis.

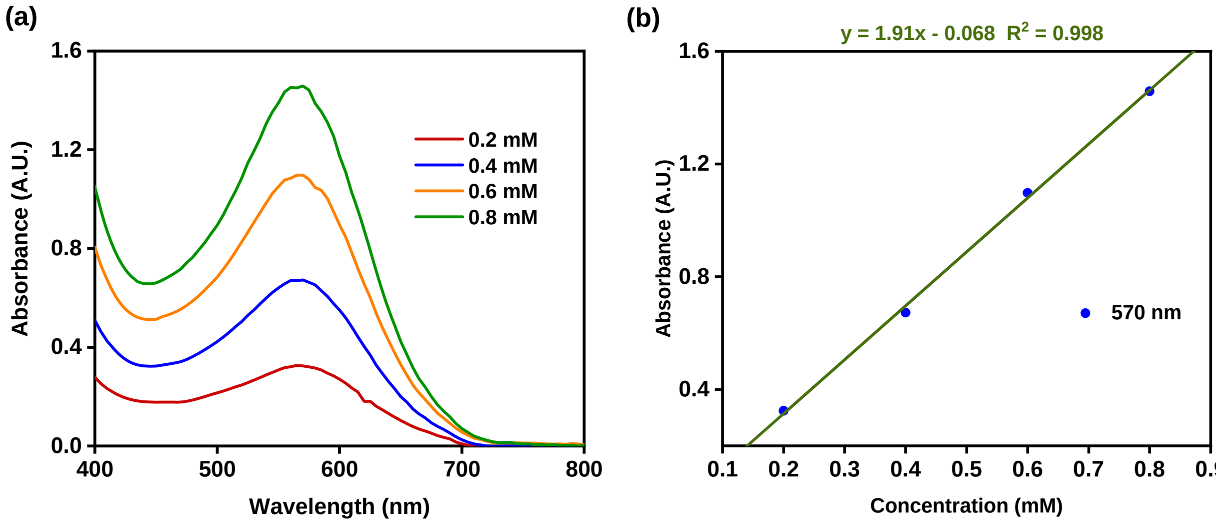

Figure S3. (a) UV-vis spectra of [Me_3NNCu]-N=CPh_2 (**3a**) in toluene at 25 °C at different concentrations (0.2 mM, 0.4 mM, 0.6 mM, 0.8 mM). (b) Beer's law plot for [Me_3NNCu]-N=CPh_2 (**3a**) depicts λ_{max} = 570 nm (ε = 1910 $M^{-1}cm^{-1}$).

Reaction of [Cl_2NN]Cu-O^{t}Bu (2b) with benzophenone imine.

[Cl_2NN]Cu-O^{t}Bu (**2b**) was synthesized following a reported procedure.[6] In a nitrogen filled glovebox, a stock solution was prepared by dissolving [Cl_2NN]Cu-O^{t}Bu (17 mg, 0.031 mmol) in toluene and diluting up to 5.00 mL (6.3 mM). 159.0 µL of this solution was diluted upto 5.0 mL with toluene to obtain a 0.2 mM solution of [Cl_2NN]Cu-O^{t}Bu. 3.00 mL of this solution was transferred into a cuvette. A stock solution of benzophenone imine was prepared by dissolving benzophenone imine (10.0 µL, 0.060 mmol) in toluene and diluting up to 10.00 mL (12.0 mM). A second stock solution was prepared by diluting 0.50 mL of 12.0 mM stock solution up to 5.0 mL (1.2 mM). Upon addition of 0.50 mL of 1.2 mM benzophenone imine stock solution (1 equiv.) to the cuvette, band at 550 nm corresponding to [Cl_2NN]Cu-N=CPh_2 (**3b**) grew in at the expense of a band at 470 nm corresponding to [Cl_2NN]Cu-O^{t}Bu (**2b**) that decayed over the course of 4.25 h at -80 °C.

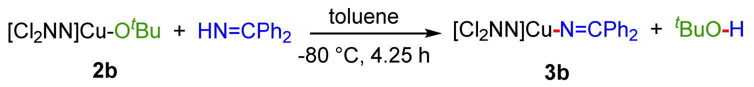

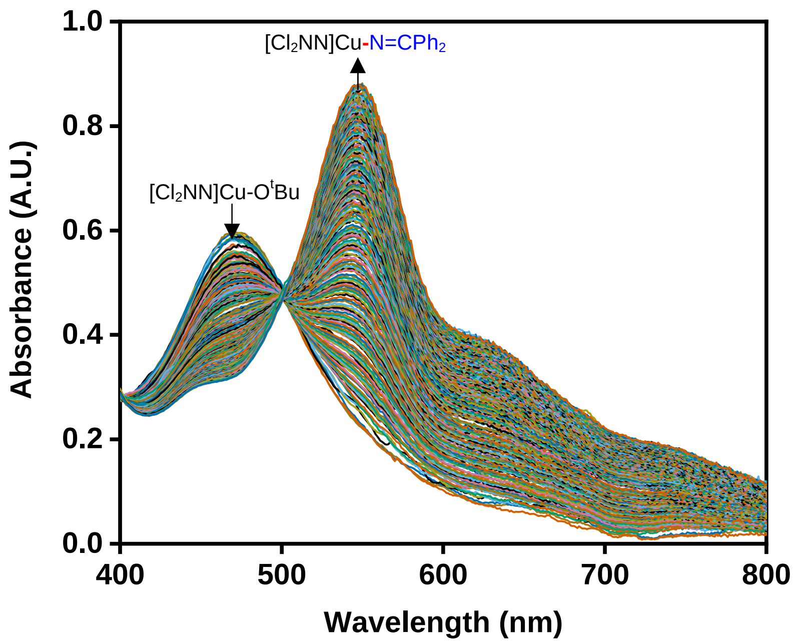

Figure S4. UV-vis spectra recorded in every 16 s during exchange reaction of [Cl_2NN]Cu-O^{t}Bu (**2b**) (0.17 mM) and benzophenone imine (1 equiv.) in toluene at -80 °C over 4.25 h.

Synthesis of $[Cl_2NN]Cu\text{-}N{=}CPh_2$ (3b).

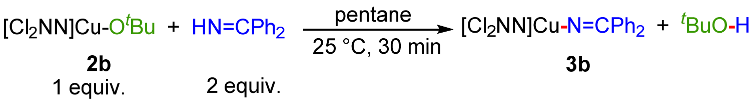

Scheme S3. Synthesis of $[Cl_2NN]Cu\text{-}N{=}CPh_2$ (**3b**).

In a nitrogen filled glovebox, to a solution of $[Cl_2NN]Cu\text{-}O^tBu$ (**2b**) (105 mg, 0.20 mmol, 1 equiv.) in pentane (10 mL) benzophenone imine (67.0 µL, 0.40 mmol, 2 equiv.) was added and stirred for 30 minutes. The solution was filtered through a pipette of Celite *ca.* 2 cm and volatiles were removed *in vacuo* to get a purple red oil of $[Cl_2NN]Cu\text{-}N{=}CPh_2$ (**3b**) in 98% yield. Attempts to crystalize this species from pentane at -37 °C afforded red color crystals in a very low yield. Due to high thermal sensitivity of crystals this species could not be characterized with X-ray crystallography or elemental analysis. This species was further characterized with UV-vis spectroscopy (Figure S5) and EPR spectroscopy (Figure S16).

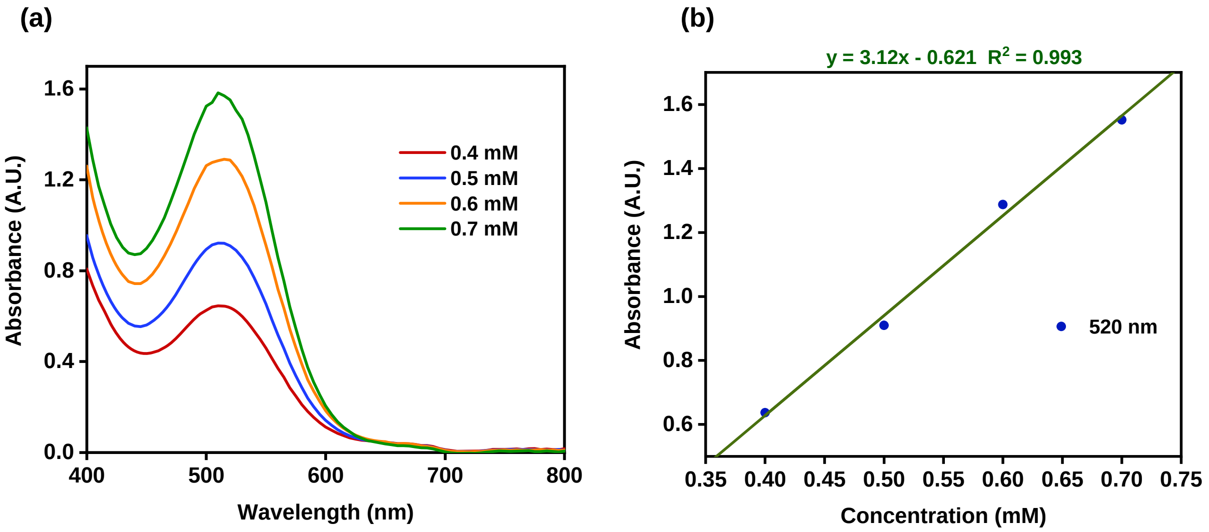

Figure S5. (a) UV-vis spectra of $[Cl_2NN]Cu\text{-}N{=}CPh_2$ (**3b**) in toluene at 25 °C at different concentrations (0.4 mM, 0.5 mM, 0.6 mM, 0.7 mM). (b) Beer's law plot for $[Cl_2NN]Cu\text{-}N{=}CPh_2$ (**3b**) depicts λ_{max} = 520 nm (ε = 3120 $M^{-1}cm^{-1}$).

Synthesis of [Me$_3$NN]Cu(NH=CPh$_2$)•THF (4a•THF)

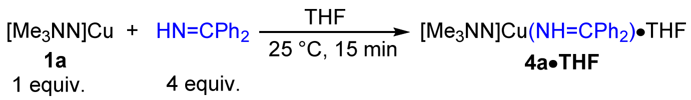

Scheme S4. Synthesis of [Me$_3$NN]Cu(NH=CPh$_2$)•THF (**4a•THF**).

In a nitrogen filled glovebox, to a stirring solution of {[Me$_3$NN]Cu}$_2$(μ-toluene) (100 mg, 0.112 mmol, 1 equiv.) in THF (10 mL) was added benzophenone imine (80.4 µL, 0.480 mmol, 4 equiv.). The resulting bright purple color solution was stirred for 15 min and filtered through a syringe filter. The filtrate was concentrated *in vacuo* to 2 mL. Bright purple color crystals were crashed out from THF and isolated in 95% yield at -35 °C. This species was further characterized with ^{1}H NMR, ^{13}C NMR, and UV-vis spectroscopies (Figure S6) as well as X-ray crystallography (Figure S19).

^{1}H NMR (400 MHz, C$_6$D$_6$): δ 7.87 (s, 1H), 7.53 (m, 2H), 7.10 (m, 1H), 7.01 (m, 3H), 6.89 (m, 6H), 6.51 (m, 2H), 4.95 (s, 1H), 3.58 (m, 2H, THF solvent), 2.26 (s, 6H), 2.22 (s, 12H), 1.80 (s, 6H), 1.42 (m, 2H, THF solvent). ^{13}C{^{1}H} NMR (100.47 MHz, C$_6$D$_6$): δ 170.86, 161.96, 149.29, 140.11, 138.13, 130.77, 130.44, 130.05, 130.01, 129.26, 129.01, 128.55, 128.46, 93.19, 67.83 (THF), 25.85 (THF), 22.75, 21.08, 19.09. Anal. calcd. for C$_{36}$H$_{40}$CuN$_3$: C, 74.77; H, 6.97; N, 7.27. Found: C, 74.71; H, 7.05; N, 7.06.

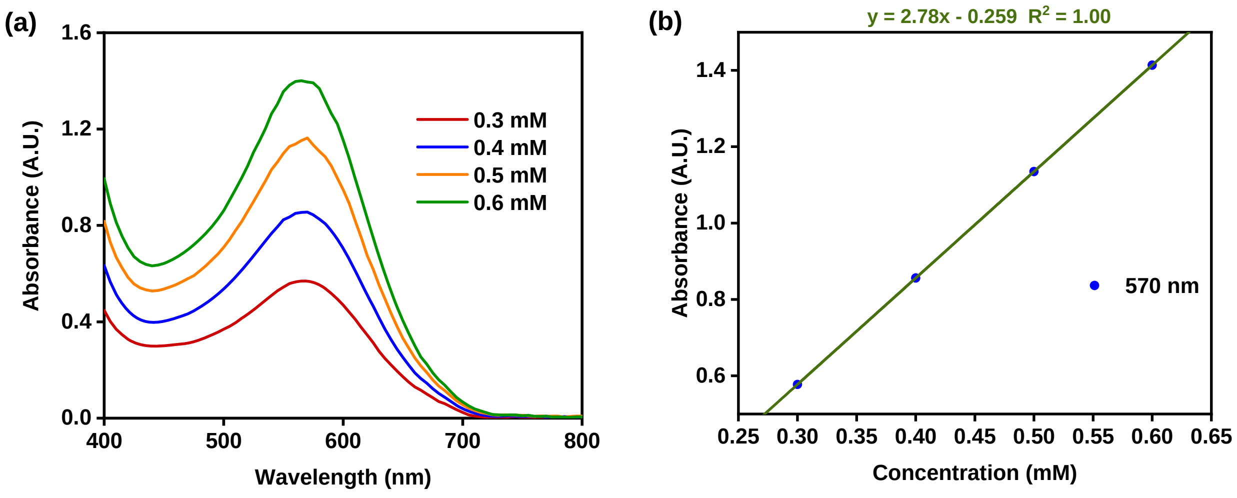

Figure S6. (a) UV-vis spectra of [Me$_3$NN]Cu-NH=CPh$_2$ (**4a**) in toluene at 25 °C at different concentrations (0.3 mM, 0.4 mM, 0.5 mM, 0.6 mM). (b) Beer's law plot for [Me$_3$NN]Cu(NH=CPh$_2$) (**4a**) depicts λ_{max} = 570 nm (ε = 2790 M^{-1}cm^{-1}).

Synthesis of [Cl_2NN]Cu(NH=CPh_2) (4b).

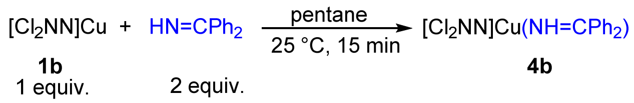

Scheme S5. Synthesis of [Cl_2NN]Cu(NH=CPh_2) (**4b**).

In a nitrogen filled glovebox, to a stirring solution of {[Cl_2NN]Cu}$_2$(*μ*-benzene) (100 mg, 0.102 mmol, 1 equiv.) in THF was added benzophenone imine (34.3 μL, 0.20 mmol). The resulting bright red color solution was stirred for 15 minutes and filtered through a syringe filter. The filtrate was concentrated *in vacuo* to 2 mL. Bright red crystals were isolated in 98% yield after allowing the solution to stand overnight at -35 °C. This species was further characterized by X-ray crystallography (Figure S20).

^{1}H NMR (400 MHz, C_6D_6): δ 8.30 (s, 1H), 7.71 (m, 2H), 7.16 (4H), 6.97 (m, 4H), 6.83 (t, J = 7.6 Hz, 2H), 6.71 (d, J = 7.5 Hz, 2H), 6.27 (t, J = 8.0 Hz, 2H), 5.01 (s, 1H), 1.86 (s, 6H). ^{13}C{^{1}H} NMR (100.47 MHz, C_6D_6): δ 195.71, 174.85, 163.74, 147.92, 140.53, 138.29, 138.16, 132.12, 130.79, 130.23, 130.20, 129.73, 129.54, 128.56, 128.45, 128.38, 128.19, 127.74, 122.63, 94.92, 23.19. Anal. calcd. for $C_{30}H_{24}Cl_4CuN_3$: C, 57.02; H, 3.83; N, 6.65. Found: C, 57.01; H, 4.04; N, 6.67.

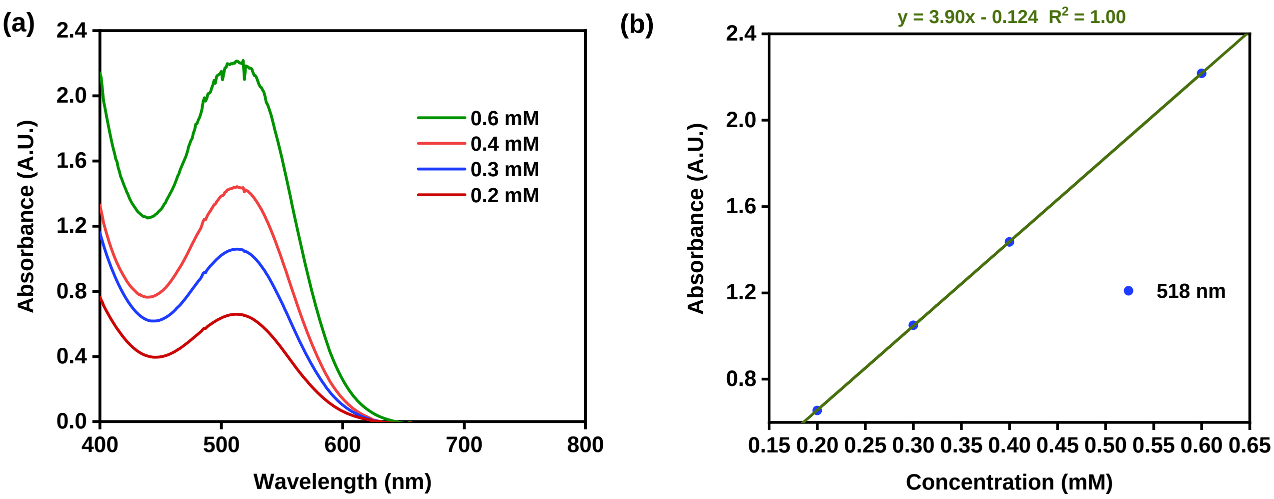

Figure S7. (a) UV-vis spectra of [Cl_2NNCu](NH=CPh_2) (**4b**) in toluene at 25 °C at different concentrations (0.2 mM, 0.3 mM, 0.4 mM, 0.6 mM). (b) Beer's law plot for [Cl_2NNCu](NH=CPh_2) (**4b**) depicts λ_{max} = 518 nm (ε = 3900 $M^{-1}cm^{-1}$).

Radical capture by $[Cu^{II}]$-N=CPh_2 intermediates

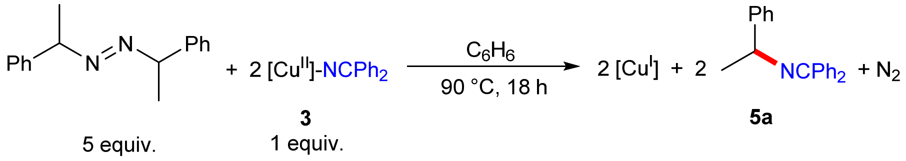

Scheme S6. Reaction of $[Cu^{II}]$-N=CPh_2 (**3**) intermediates with (*E/Z*)-azobis(α-phenylethane).

<u>Reaction of $[Me_3NN]$Cu-N=CPh_2 (**3a**) with (*E/Z*)-azobis(α-phenylethane)</u>

In a nitrogen filled glovebox, $[Me_3NN]$Cu-N=CPh_2 (**3a**) (46 mg, 0.08 mmol, 1 equiv.) was dissolved in benzene (0.25 mL) in a thick walled 5 mL pressure vessel and (*E/Z*)-azobis(α-phenylethane) (42 mg, 0.18 mmol, 2 equiv.) was added. The mixture was stirred for 18 h at 90 °C. Reaction mixture was cooled to room temperature quenched by opening to air. Naphthalene (10 mg, 0.08 mmol, 1 equiv.) was added to the quenched reaction mixture as an internal standard. The yield was determined by ^{1}H NMR in $CDCl_3$ against the internal standard to be 40% by considering the peak at δ 4.58 ppm (benzylic 1H).

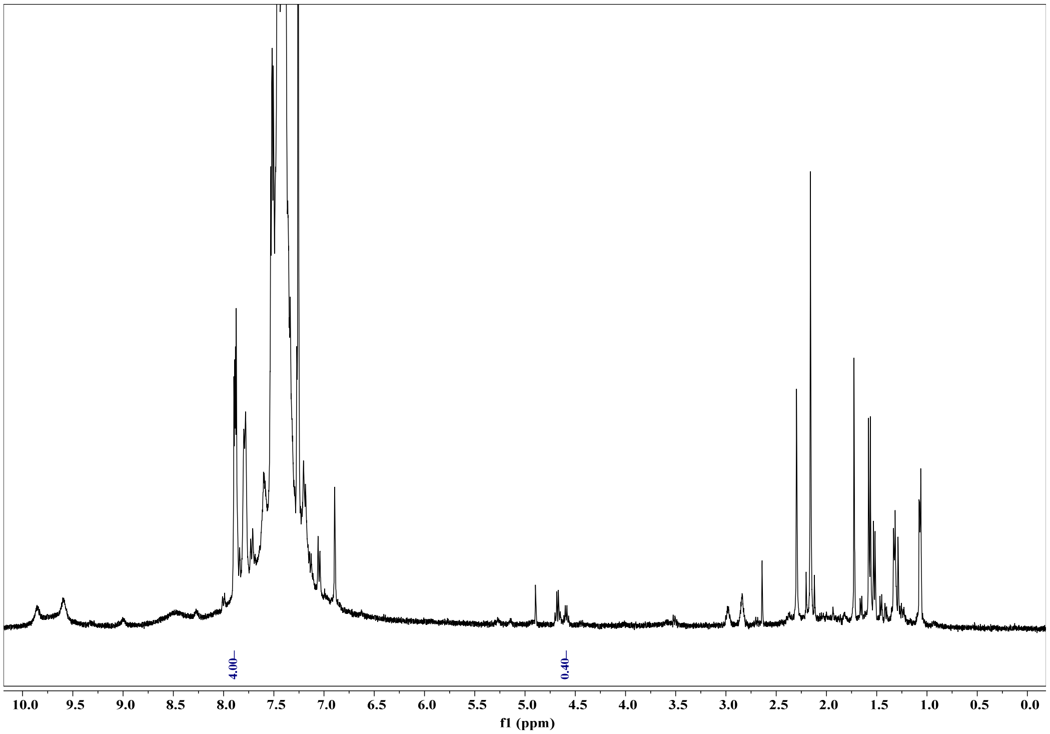

Figure S8. ^{1}H NMR spectrum (400 MHz, $CDCl_3$) of the crude reaction mixture of $[Me_3NN]$Cu-N=CPh_2 (**3a**) with (*E/Z*)-azobis(α-phenylethane).

Reaction of [Cl_2NN]Cu-N=CPh_2 (**3b**) with (*E/Z*)-azobis(α-phenylethane)

In a nitrogen filled glovebox, [Cl_2NN]Cu-N=CPh_2 (**3b**) (51 mg, 0.08 mmol, 1 equiv.) was dissolved in benzene (1.00 mL) in a thick walled 5 mL pressure vessel and (*E/Z*)-azobis(α-phenylethane) (20 mg, 0.08 mmol, 5 equiv.) was added. The mixture was stirred for 18 h at 90 °C. Reaction mixture was cooled to room temperature quenched by opening to air. Naphthalene (10 mg, 0.08 mmol, 1 equiv.) was added to the quenched reaction mixture as an internal standard. The yield was determined by ^{1}H NMR in $CDCl_3$ against the internal standard to be 74% considering the peak at δ 4.58 ppm (benzylic 1H).

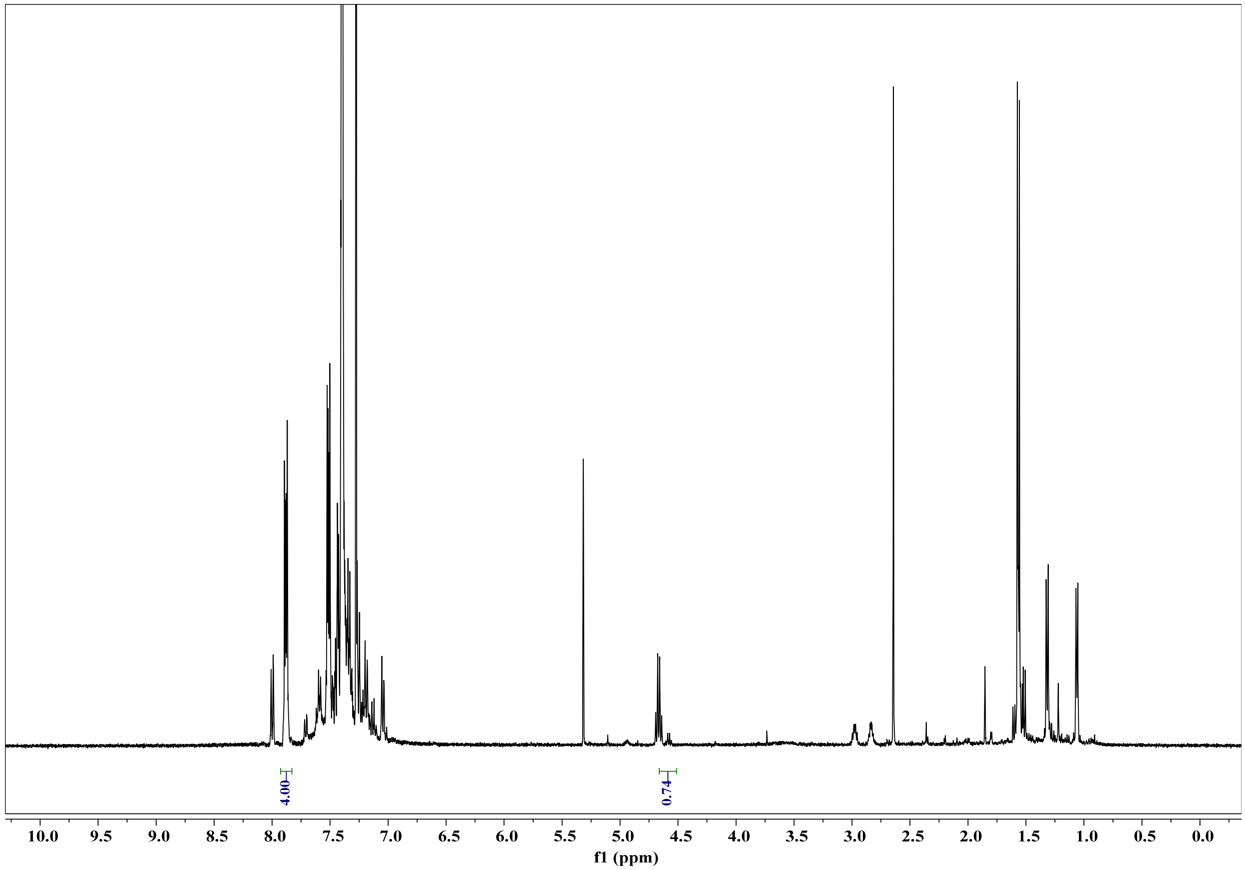

Figure S9. ^{1}H NMR spectrum (400 MHz, $CDCl_3$) of the crude reaction mixture of [Cl_2NN]Cu-N=CPh_2 (**3b**) with (*E/Z*)-azobis(α-phenylethane).

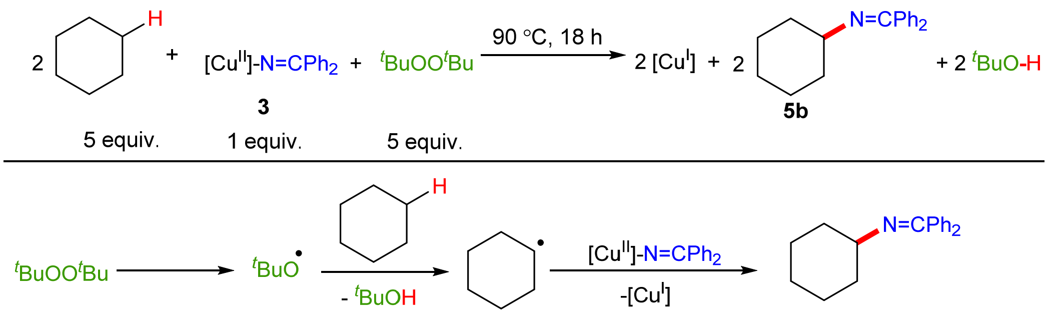

Scheme S7. Reaction of $[Cu^{II}]$-N=CPh_2 (**3**) intermediates with ${}^tBuOO{}^tBu$ and cyclohexane.

Reaction of $[Me_3NN]$Cu-N=CPh_2 (**3a**) with ${}^tBuOO{}^tBu$ and cyclohexane

In a nitrogen filled glovebox, $[Me_3NN]$Cu-N=CPh_2 (**3a**) (95 mg, 0.165 mmol, 1 equiv.) was dissolved in cyclohexane (87.0 μL, 5 equiv.) in a thick walled 5 mL pressure vessel and ${}^tBuOO{}^tBu$ (151.6 μL, 0.825 mmol, 5 equiv.) was added. The mixture was stirred for 18 h at 90 °C, cooled to room temperature and quenched by opening to air. Naphthalene (21 mg, 0.165 mmol, 1 equiv.) was added to the quenched reaction mixture as an internal standard. The yield was determined by ^{1}H NMR in $CDCl_3$ against the internal standard to be 41% by considering the peak at δ 3.11 ppm (tertiary 1H).

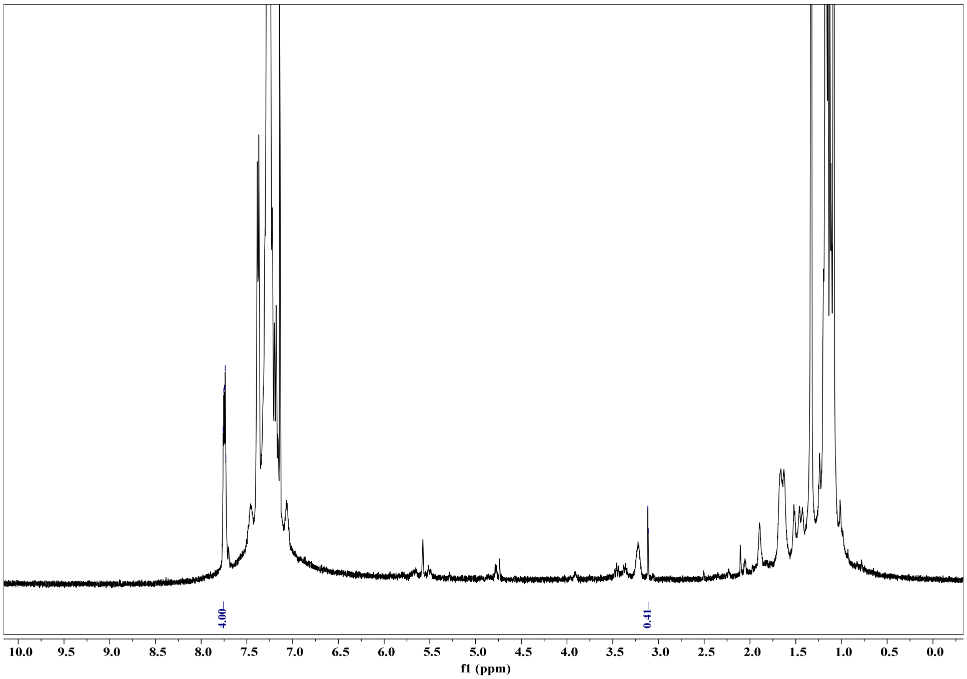

Figure S10. ^{1}H NMR spectrum (400 MHz, $CDCl_3$) of the crude reaction mixture of $[Me_3NN]$Cu-N=CPh_2 (**3a**) with ${}^tBuOO{}^tBu$ and cyclohexane.

Reaction of $[Cl_2NN]Cu-N=CPh_2$ (**3b**) with $^tBuOO^tBu$ and cyclohexane

In a nitrogen filled glovebox, $[Cl_2NN]Cu-N=CPh_2$ (**3b**) (100 mg, 0.159 mmol, 1 equiv.) was dissolved in cyclohexane (85.9 μL, 5 equiv.) in a thick walled 5 mL pressure vessel and $^tBuOO^tBu$ (146.0 μL, 0.795 mmol, 5 equiv.) was added. The mixture was stirred for 18 h at 90 °C, cooled to room temperature and quenched by opening to air. Naphthalene (20 mg, 0.159 mmol, 1 equiv.) was added to the quenched reaction mixture as an internal standard. The yield was determined by ^1H NMR in $CDCl_3$ against the internal standard to be 58% by considering the peak at δ 3.11 ppm (tertiary 1H).

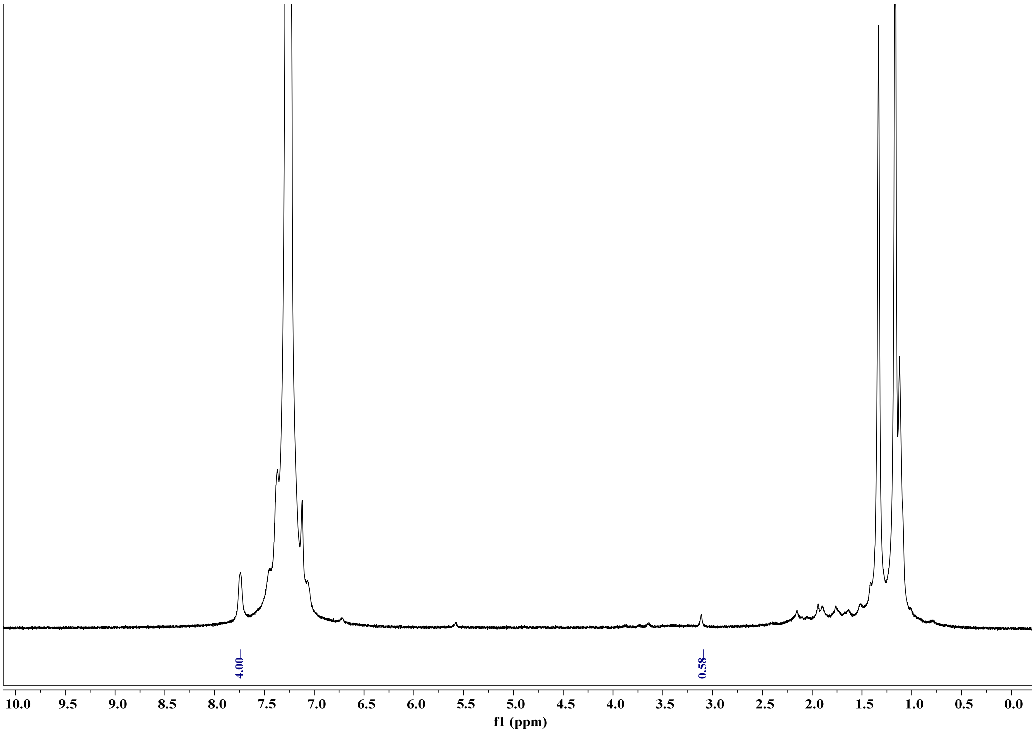

Figure S11. ^1H NMR spectrum (400 MHz, $CDCl_3$) of the crude reaction mixture of $[Cl_2NN]Cu-N=CPh_2$ (**3b**) with $^tBuOO^tBu$ and cyclohexane.

N-N coupling of [Cu^{II}]-N=CPh$_2$ to form benzophenone azine – Quantification of benzophenone azine byproduct

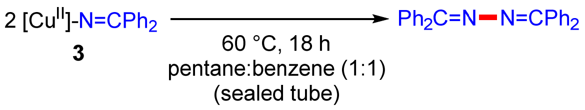

Scheme S8. N-N coupling of [Cu^{II}]-N=CPh$_2$ (**3**) to form benzophenone azine.

N-N coupling of [Me$_3$NN]Cu-N=CPh$_2$ (**3a**) to form benzophenone azine

In a nitrogen filled glovebox, a sealed pressure vessel was charged with [Me$_3$NN]Cu-N=CPh$_2$ (**3a**) (68 mg, 0.117 mmol) and a 1:1 mixture of pentane and benzene (1.40 mL). The mixture was stirred at 60 °C for 18 h, cooled to room temperature and quenched by opening to air. Benzophenone azine was isolated with column chromatography on silica with 5% EtOAc in hexanes in 66% yield. ^{1}H NMR and ^{13}C{^{1}H} NMR data of benzophenone azine match literature reported values.[7]

^{1}H NMR (400 MHz, CDCl$_3$): δ 7.48 (m, 4H), 7.39 (m, 6H), 7.33 (m, 6H), 7.29 (m, 4H). ^{13}C{^{1}H} NMR (100.47 MHz, CDCl$_3$): δ 159.03, 138.33, 135.68, 129.77, 129.45, 128.80, 128.77, 128.15, 128.00.

Coupling of [Cl$_2$NN]Cu-N=CPh$_2$ (**3b**) to form benzophenone azine

In a nitrogen filled glovebox, a pressure vessel was charged with [Cl$_2$NN]Cu-N=CPh$_2$ (**3b**) (71 mg, 0.112 mmol) and a 1:1 mixture of pentane and benzene (1.40 mL). The mixture was stirred at 60 °C for 18 h, cooled to room temperature and quenched by opening to air. Benzophenone azine was isolated with column chromatography on silica with 5% EtOAc in hexanes in 90% yield.

Synthesis of Benzophenone Imine Derivatives

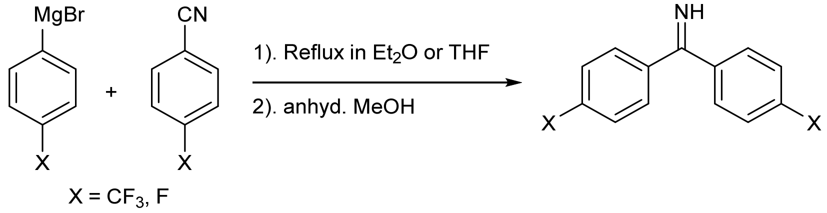

Scheme S9. Synthesis of benzophenone imine derivatives.

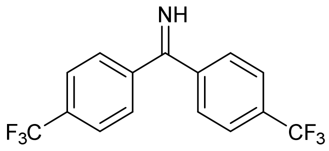

bis[4-(trifluoromethyl)phenyl]methanimine. The synthesis was carried out according to a literature reported procedure[8] with several modifications. In a nitrogen filled glovebox, 4-bromobenzotrifluoride (1.86 mL, 13.3 mmol, 1 equiv.) was dissolved in diethyl ether (8 mL). Half of this solution (*ca.* 4 mL) was transferred into a 100 mL pressure vessel containing magnesium turnings (0.320 g, 13.3 mmol, 1 equiv.). Upon addition of few crystals of iodine, the reaction started and the mixture was stirred at room temperature for 20 min. The remaining 4-bromobenzotrifluoride solution was added to this mixture and stirring was continued while refluxing outside the glovebox for 1 h. The reaction was cooled to room temperature and taken into the glovebox. A solution of 4-(trifluoromethyl)benzonitrile (2.29 g, 13.3 mmol, 1 equiv.) in toluene (2 mL) was added slowly at room temperature and refluxed for 20 h. Inside the glovebox, reaction mixture was cooled to -35 °C and dry MeOH (4 mL) was added slowly. The mixture was stirred for 30 minutes and the solid formed was filtered off over a pad of Celite. The filtrate was concentrated to dryness. The light brown solid was recrystallized with pentane to get pure bis[4-(trifluoromethyl)phenyl]methanimine as a light orange solid with 60% yield in multiple crops. ^{1}H NMR and ^{13}C{^{1}H} NMR spectra are consistent with data reported in literature.[8]

^{1}H NMR (400 MHz, $CDCl_3$): δ 10.08 (s, 1H), 7.84 (d, 2H), 7.71 (dd, J = 7.96, 15.45 Hz, 4H), 7.52 (d, J = 7.89 Hz, 2H). ^{13}C{^{1}H} NMR (100.47 MHz, $CDCl_3$): δ 179.94, 143.29, 141.24, 132.58, 129.50, 127.97, 125.68, 123.89 (q, $^{1}J_{C\text{-}F}$ = 272.3 Hz). ^{19}F NMR (376 MHz, $CDCl_3$): δ -63.25 (s, 6F).

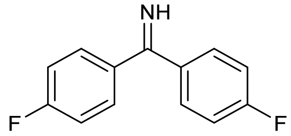

Bis(4-fluorophenyl)methanimine. The synthesis was carried out according to a literature reported procedure[9] with minor

modifications. In a nitrogen filled glovebox, 4-fluorobenzonitrile (1.20 g, 10.0 mmol, 1 equiv.) in THF (10 mL) and 4-fluorophenyl magnesium bromide (10.0 mL, 1.0 M, 10.0 mmol, 1 equiv.) was mixed together in a 100 mL pressure vessel. The mixture was stirred at 80 °C for 12 h. Reaction was cooled to room temperature and anhydrous MeOH (4 mL) was slowly added and stirred for 45 min. The off-white solid crashed out was filtered-off over a pad of Celite. The filtrate was concentrated under vacuum and the remainder was purified with flash column chromatography with 1.5% triethyl amine in hexanes and ethyl acetate as the eluent. The titled compound was isolated as a yellow oil in 65% yield with 80% purity. ^{1}H NMR, ^{13}C{^{1}H}, and ^{19}F NMR spectra are consistent with data reported in literature.[10]

^{1}H NMR (400 MHz, $CDCl_3$): δ 9.64 (bs, 1H), 7.50 (m, 4H), 7.10 (t, J = 8.5 Hz, 4H). ^{13}C{^{1}H} NMR (100.47 MHz, $CDCl_3$): δ 176.17, 165.53 (d, $^{1}J_{C\text{-}F}$ = 254.36 Hz), 132.57, 132.66, 115.69 (d, $^{2}J_{C\text{-}F}$ = 21.9 Hz). ^{19}F NMR (376 MHz, $CDCl_3$): δ -109.96 (m, 2F).

3. Catalytic C-H amination

Optimization of the amination protocol

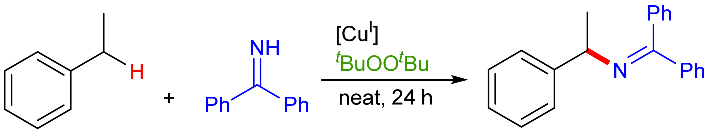

5a

Scheme S10. Representative reaction for optimization of C-H amination reaction.

In a nitrogen filled glovebox, benzophenone imine (68.5 µL, 0.408 mmol, 1 equiv.) and β-diketiminato catalyst [Cu^{I}] (5 mol%) (see Table S3) was dissolved in ethylbenzene (0.50 mL, 4.08 mmol, 10 equiv.) in a thick walled 5 mL pressure vessel to give a bright red color solution. To the solution, ^{t}BuOO^{t}Bu (90.0 µL, 0.490 mmol, 1.2 equiv.) was added and the pressure vessel was immediately sealed, heated at 90 °C. After 24 h, the reaction was allowed to cool to room temperature and quenched by exposure to air. Naphthalene (52 mg, 0.408 mmol, 1 equiv.) was added to the quenched reaction mixture as an internal standard. The yield was determined by ^{1}H NMR spectroscopy considering the peak at δ 4.58 ppm (1H) with naphthalene (1 equiv. based on the imine) as a standard.

Table S1. Catalyst screening for C-H amination of ethylbenzene with benzophenone imine.

Entry	Catalyst (X, R^1, R^2)		Catalyst loading (mol%)	Ethylbenzene equiv.	Yield (%)
**1**	$[Me_3NN]Cu$ (CH_3, CH_3, CH_3)	**1a**	5	50	34
**2**	$[Cl_2NN]Cu$ (CH_3, Cl, H)	**1b**	5	50	60
**3**	$[^iPr_2NN]Cu$ (CH_3, ^iPr, H)	**1c**	5	50	30
**4**	$[Cl_2NN_{F6}]Cu$ (CF_3, Cl, H)	**1d**	5	50	42
**5**	$[Cl_2NN]Cu$ (CH_3, Cl, H)	**1b**	5	100	62
**6**	$[Cl_2NN]Cu$ (CH_3, Cl, H)	**1b**	5	10	30
**7**	$[Cl_2NN]Cu$ (CH_3, Cl, H)	**1b**	5	1	Trace
**8**	$[Cl_2NN]Cu$ (CH_3, Cl, H)	**1b**	10	50	41
**9**	$[Cl_2NN]Cu$ (CH_3, Cl, H)	**1b**	2.5	50	55
**10**	$[Cl_2NN]Cu$ (CH_3, Cl, H)	**1b**	1	10	44
**11**	$[Cl_2NN]Cu$ (CH_3, Cl, H)	**1b**	1	10	52*

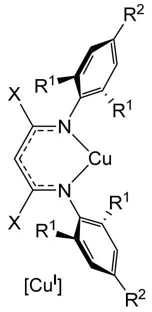

Reaction conditions: 1 equiv. benzophenone imine, 1.2 equiv. $^tBuOO^tBu$, 90 °C, 24 h. *With 2 equiv. of $^tBuOO^tBu$.

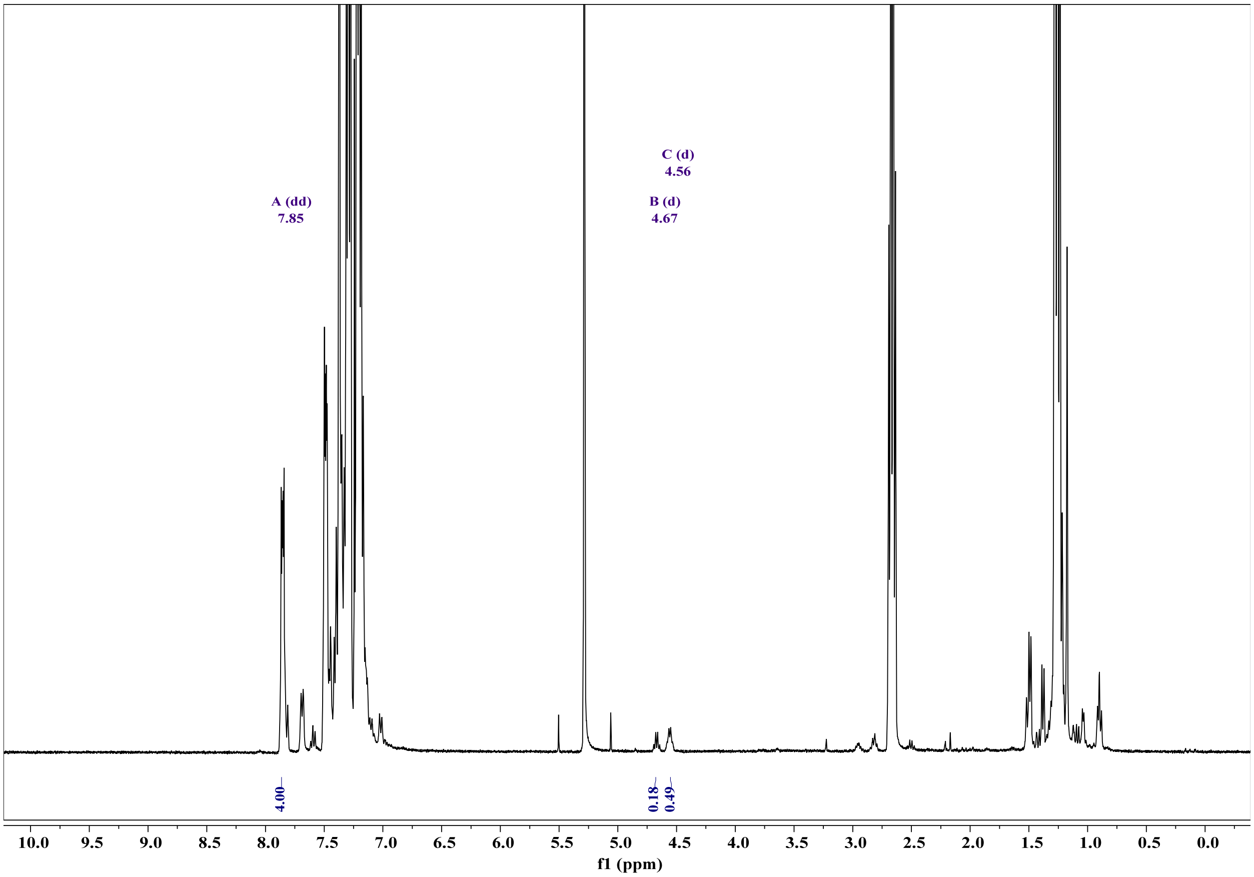

Figure S12. ^1H NMR spectrum (400 MHz, $CDCl_3$) of the crude reaction mixture of ethylbenzene with benzophenone imine under optimized conditions.

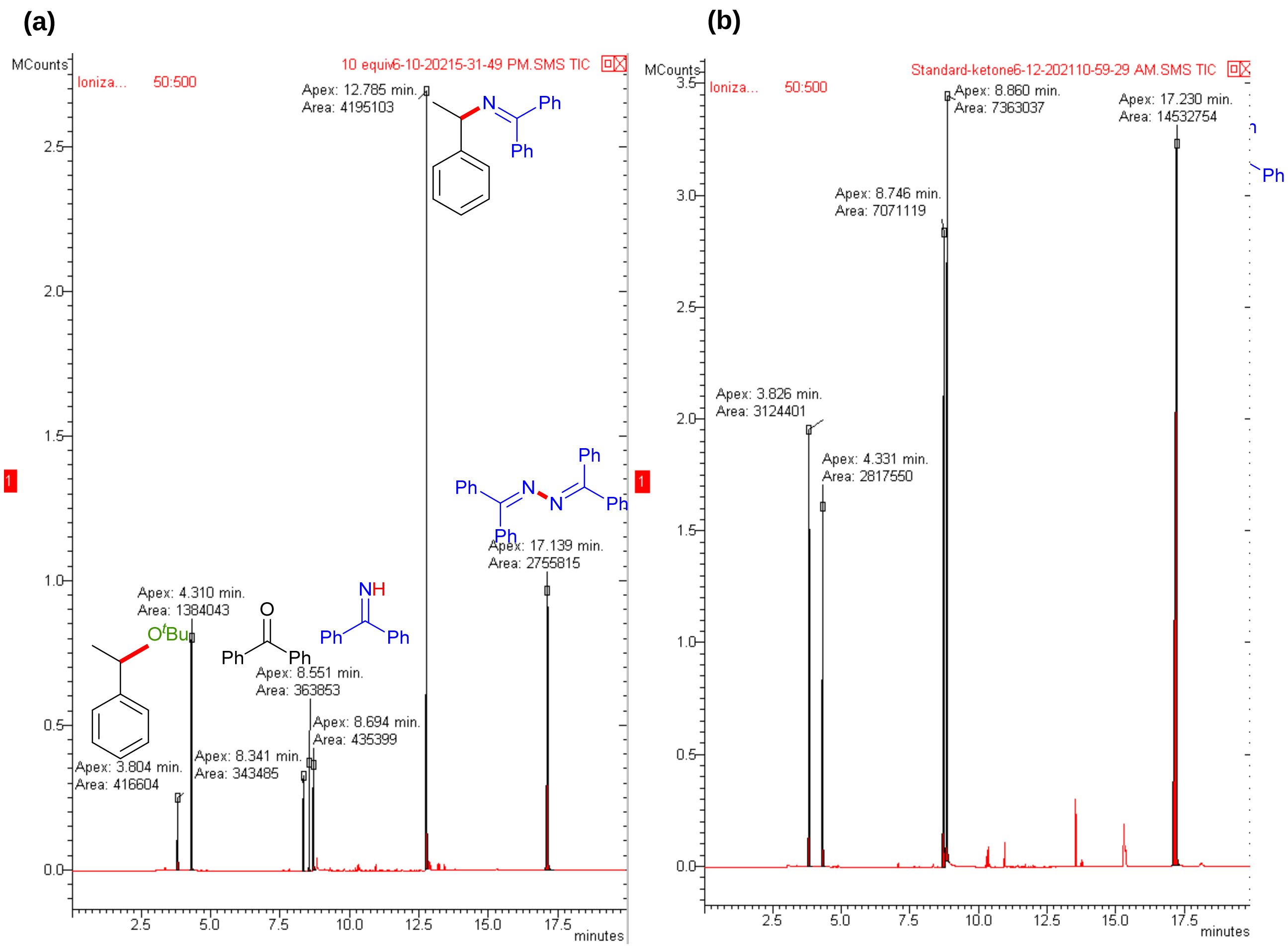

Figure S13. GC-MS* spectra of (a) Crude reaction mixture of ethylbenzene under optimized conditions. (b) Standard samples of reactants and products.

Catalytic C-H amination with benzophenone imine derivatives

<u>Representative procedure for catalytic C-H amination of ethylbenzene with benzophenone imine derivatives</u>

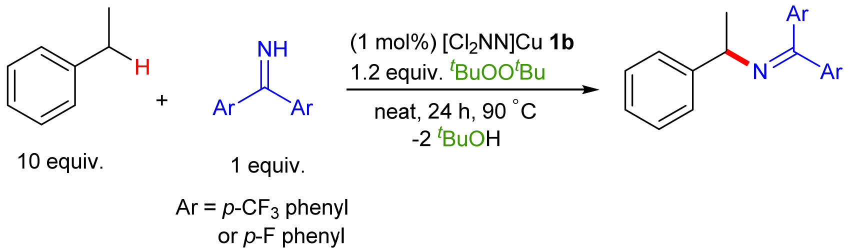

Scheme S11. Catalytic C-H amination reaction of ethylbenzene with benzophenone imine derivatives.

In a nitrogen filled glovebox, a stock solution of $[Cl_2NN]Cu$ was prepared by dissolving $[Cl_2NN]Cu$ (20 mg, 0.040 mmol) in benzene (1.00 mL) and 99.5 μL (2 mg, 0.004 mmol) of this

solution was transferred into a thick walled 5 mL pressure vessel. To this solution, benzophenone imine derivative (0.408 mmol, 1 equiv.), ethylbenzene (0.50 mL, 2.04 mmol, 10 equiv.) and ^{t}BuOO^{t}Bu (90.0 µL, 0.49 mmol, 1.2 equiv.) were added and the pressure vessel was immediately sealed, heated at 90 °C. After 24 h the reaction mixture was cooled down to room temperature and exposed to air to quench the reaction. Naphthalene (52 mg, 0.408 mmol, 1 equiv.) was added to the quenched reaction mixture as an internal standard and yield was determined with ^{1}H NMR. Amination products were isolated via prep TLC with 3% triethyl amine, 5% ethyl acetate in hexane. TLC plates were basified with a solution of 5% triethyl amine in hexane prior to use.

Table 2 entry 5a-CF$_3$. *N*-(1-phenylethyl)-1,1-bis(4-(trifluoromethyl)phenyl)methanimine. See representative reaction procedure on page S17. With bis[(4-trifluoromethyl)phenyl]methanimine (129 mg) yield of the title compound was determined to be 51% by ^{1}H NMR spectroscopy considering the peak at δ 4.50 ppm (1H). The title compound was isolated as a pale-yellow oil. ^{1}H NMR (400 MHz, CDCl$_3$): δ 7.75 (m, 4H), 7.59 (d, J = 8.3 Hz, 2H), 7.32 (d, J = 4.4 Hz, 3H), 7.27 (s, 1H), 7.24 (m, 2H), 4.46 (q, J = 6.4 Hz, 1H), 1.49 (d, J = 6.4 Hz, 3H). ^{13}C{^{1}H} NMR (100.47 MHz, CDCl$_3$): δ 163.43, 145.40, 142.45, 140.05, 132.08 (q, $^2J_{C\text{-}F}$ = 33.15 Hz), 131.20 (q, $^2J_{C\text{-}F}$ = 32.1 Hz), 128.83, 128.68, 128.24, 127.11, 126.65, 125.94 (q, $^3J_{C\text{-}F}$ = 3.8 Hz), 125.28 (q, $^3J_{C\text{-}F}$ = 3.8 Hz), 124.12 (q, $^1J_{C\text{-}F}$ = 272.3 Hz), 124.02 (q, $^1J_{C\text{-}F}$ = 273.3 Hz), 62.17, 25.24. ^{19}F NMR (376 MHz, CDCl$_3$): δ -63.28 (s, 3F), -63.35 (s, 3F). HRMS (CI) *m/z* calcd for $C_{23}H_{17}F_6N$ (M+H)$^+$ 422.1338, found 422.1342.

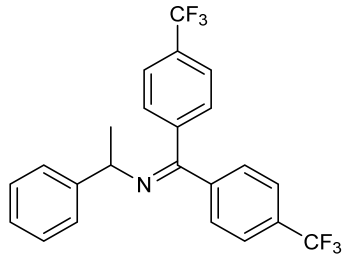

Table 2 entry 5a-F. *N*-(1-phenylethyl)-1,1-bis(4-fluorophenyl)-methanimine. See representative reaction procedure on page S17. With bis(4-fluorophenyl)methanimine (72.2 µL) yield of the title compound was determined to be 36% by ^{1}H NMR spectroscopy considering the peak at δ 4.51 ppm (1H). The title compound was isolated as a pale yellow oil. ^{1}H NMR (400 MHz, CDCl$_3$): δ 7.64 (m, 2H), 7.31 (m, 4H), 7.23 (m, 1H), 7.16 (m, 2H), 7.09 (m, 2H), 7.01 (m, 2H), 4.48 (q, J = 6.5 Hz, 1H), 1.47 (d, J = 6.4 Hz, 3H). ^{13}C{^{1}H} NMR (100.47 MHz, CDCl$_3$): δ 164.20 (d, $^1J_{C\text{-}F}$ = 250.2

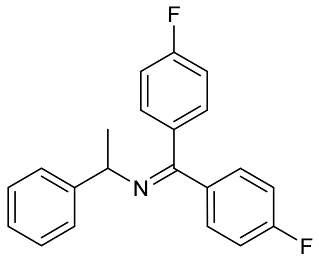

Hz), 164.02, 162.76 (d, $^{1}J_{C\text{-}F}$ = 248.2 Hz), 146.03, 136.27 (d, $^{4}J_{C\text{-}F}$ = 2.9 Hz), 132.70 (d, $^{4}J_{C\text{-}F}$ = 3.8 Hz), 130.61 (d, $^{3}J_{C\text{-}F}$ = 8.5 Hz), 129.68 (d, $^{3}J_{C\text{-}F}$ = 8.0 Hz), 128.55, 126.84, 126.70, 115.84 (d, $^{2}J_{C\text{-}F}$ = 22.1 Hz), 115.14 (d, $^{2}J_{C\text{-}F}$ = 21.1 Hz), 61.68, 25.27. ^{19}F NMR (376 MHz, $CDCl_3$): δ -111.24 (m, 1F), -112.48 (m, 1F). HRMS (CI) *m/z* calcd for $C_{21}H_{17}F_2N$ $(M+H)^+$ 322.1402, found 322.1406.

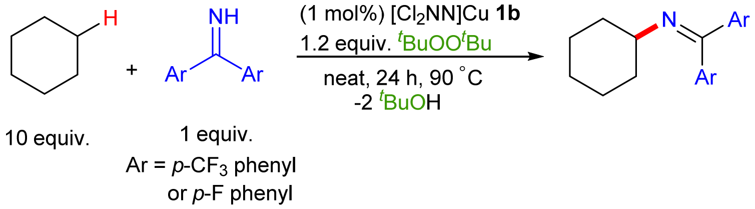

Scheme S12. Catalytic C-H amination reaction of cyclohexane with benzophenone imine derivatives.

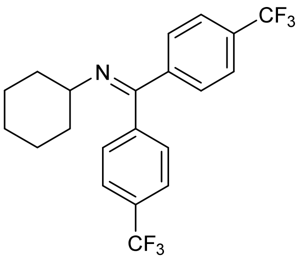

Table 2 entry 5b-CF$_3$. *N*-cyclohexyl-1,1-bis(4-(trifluoromethyl)phenyl)methanimine. See representative reaction procedure on page S17. To 113.0 μL (2.3 mg, 0.005 mmol) of the [Cl_2NN]Cu stock solution in a thick walled 5 mL pressure vessel, bis[(4-trifluoromethyl)phenyl]methanimine (147 mg, 0.463 mmol), cyclohexane (0.50 mL, 4.64 mmol) and ^{t}BuOO^{t}Bu (102.3 μL, 0.557 mmol) were added. Naphthalene (59 mg, 0.463 mmol, 1 equiv.) was added to the quenched reaction mixture as an internal standard. Yield of the title compound was determined to be 56% by ^{1}H NMR considering the peak at δ 3.16 ppm (1H). The titled compound was isolated *via* fast column chromatography with 10% dichloromethane in hexanes as a yellow solid. ^{1}H NMR (400 MHz, $CDCl_3$): δ 7.75 (d, *J* = 8.0 Hz, 2H), 7.67 (d, 2H), 7.57 (d, *J* = 8.3 Hz, 2H), 7.28 (d, *J* = 7.9 Hz, 2H), 3.16 (tt, *J* = 9.4, 4.8 Hz, 1H), 1.76 (m, 2H), 1.60 (m, 5H), 1.22 (m, 3H). ^{13}C{^{1}H} NMR (100.47 MHz, $CDCl_3$): δ 162.87, 142.85, 140.44, 131.85 (q, $^{2}J_{C\text{-}F}$ = 33.2 Hz), 130.93 (q, $^{2}J_{C\text{-}F}$ = 33.2 Hz), 128.66, 128.25, 125.90 (q, $^{3}J_{C\text{-}F}$ = 3.8 Hz), 125.24 (q, $^{3}J_{C\text{-}F}$ = 3.8 Hz), 124.16 (q, $^{1}J_{C\text{-}F}$ = 272.3 Hz), 124.04 (q, $^{1}J_{C\text{-}F}$ = 272.3 Hz), 61.96, 33.93 (2C), 25.72, 24.29 (2C). ^{19}F NMR (376 MHz, $CDCl_3$): δ -63.23 (s, 3F), -63.31 (s, 3F). HRMS (CI) *m/z* calcd for $C_{21}H_{19}F_6N$ $(M+H)^+$ 400.1494, found 400.1497.

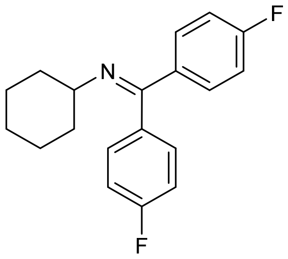

Table 2 entry 5b-F. *N*-cyclohexyl-1,1-bis(4-fluorophenyl)methanimine. See representative reaction procedure

on page S17. To 113.0 µL (2.3 mg, 0.005 mmol) of the Cl_2NNCu stock solution in a thick walled 5 mL pressure vessel, bis(4-fluorophenyl)methanimine (82.0 µL, 0.463 mmol), cyclohexane (0.50 mL, 4.63 mmol) and ${}^tBuOO^tBu$ (102.3 µL, 0.557 mmol) were added. Naphthalene (59.4 mg, 0.464 mmol, 1 equiv.) was added to the quenched reaction mixture as an internal standard. The yield of the title compound was determined to be 39% by ^1H NMR considering the peak at δ 3.17 ppm (1H). The title compound was isolated as a pale-yellow oil. ^1H NMR (400 MHz, $CDCl_3$): δ 7.55 (m, 2H), 7.14 (m, 4H), 6.99 (m, 2H), 3.15 (m, 1H), 1.75 (m, 2H), 1.58 (m, 6H), 1.23 (m, 2H). $^{13}C\{^1H\}$ NMR (100.47 MHz, $CDCl_3$): δ 164.03 (d, $^1J_{C\text{-}F}$ = 250.2 Hz), 163.53, 162.62 (d, $^1J_{C\text{-}F}$ = 248.2 Hz), 136.63 (d, $^4J_{C\text{-}F}$ = 3.0 Hz), 133.09 (d, $^4J_{C\text{-}F}$ = 3.0 Hz), 130.40 (d, $^3J_{C\text{-}F}$ = 9.0 Hz), 129.65 (d, $^3J_{C\text{-}F}$ = 8.0 Hz), 115.78 (d, $^2J_{C\text{-}F}$ = 21.1 Hz), 115.09 (d, $^2J_{C\text{-}F}$ = 21.1 Hz), 61.61, 34.07 (2C), 25.79, 24.51 (2C). ^{19}F NMR (376 MHz, $CDCl_3$): δ -111.76 (m, 1F), -112.89 (m, 1F). HRMS (CI) *m/z* calcd for $C_{19}H_{19}F_2N$ $(M+H)^+$ 300.1558, found 300.1559.

Catalytic C-H amination with benzophenone imine

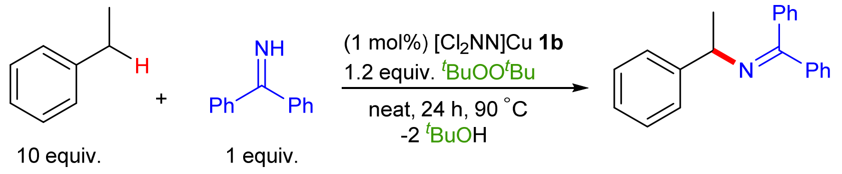

Scheme S13. Representative C-H amination reaction of ethylbenzene.

Representative procedure for C-H amination of ethylbenzene.

In a nitrogen filled glovebox, a stock solution of $[Cl_2NN]Cu$ was prepared by dissolving $\{[Cl_2NN]Cu\}_2(\mu\text{-benzene})$ (20 mg, 0.02 mmol) in benzene (1.00 mL) and 99.5 µL (2 mg, 1% based on imine) of this solution was transferred into a thick walled 5 mL pressure vessel. To this solution, benzophenone imine (68.5 µL, 0.408 mmol, 1 equiv.), ethylbenzene (0.50 mL, 4.08 mmol, 10 equiv.) and ${}^tBuOO^tBu$ (90.0 µL, 0.489 mmol, 1.2 equiv.) were added and the pressure vessel was immediately sealed, heated at 90 °C. After 24 h, the reaction mixture was cooled down to room temperature and exposed to air to quench the reaction.

Purification of amination products was carried out following three general procedures.

General purification procedure A – Isolation of ketimine products R-N=CAr_2

Products were isolated via fast column chromatography with 5% EtOAc in hexanes on silica.*

**A high flow rate (50 mL/min) should be used to ensure product separation from the benzophenone azine byproduct and to minimize ketimine hydrolysis to primary amines.*

General purification procedure B – Isolation of ketimine products R-N=CAr_2

The reaction mixture was transferred into a 4 dram vial with dichloromethane. One eighth of the reaction mixture was used to run a preparatory TLC with 3% triethyl amine, 5% ethyl acetate in hexane. TLC plates were basified with a solution of 5% triethyl amine in hexane prior to use.

General purification procedure C – Isolation of deprotected amines as HCl acid salts R-$NH_3^+Cl^-$

Following a reported procedure,[11] the reaction mixture was passed through a pipette of silica (*ca.* 1 cm) with ethyl acetate and volatiles were removed *in vacuo.* The residue was suspended in MeOH (6 mL) and 1M HCl solution (2-3 mL). The mixture was stirred for 12 h at 60 °C. MeOH was evaporated under reduced pressure and the aqueous mixture was extracted with hexane (5 mL × 4). The aqueous layer was evaporated *in vacuo*. Resulting residue was resuspended in distilled water (3 mL) and basified with K_2CO_3 (1 g). The aqueous solution was extracted with Et_2O (5 mL × 2) and combined organic layers were dried over Na_2SO_4. Solvent was removed under reduced pressure at room temperature and the resulting amine was dissolved in pentane (4 mL). Upon addition of etheral HCl solution (2 M, 1 mL) a white precipitate formed and it was filtered off to isolate the amination products as hydrochloric acid salts.

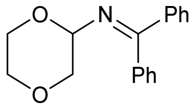

Table 3 entry 6a. *N*-(1,4-dioxan-2-yl)-1,1-diphenylmethanimine. See representative reaction procedure on page S19. To 143.0 µL of [Cl_2NN]Cu stock solution (2.9 mg, 1 mol% based on imine), benzophenone imine (98.0 µL, 0.584 mmol) 1,4-dioxane (0.50 mL, 5.84 mmol), ^{t}BuOO^{t}Bu (128.8 µL, 0.701 mmol) were added. The title compound was isolated as a white solid in 51% yield following the general purification procedure A. The compound was recrystallized from dichloromethane to get X-ray quality crystals. ^{1}H NMR (400 MHz, $CDCl_3$): δ 7.67 (m, 2H), 7.47 (m, 2H), 7.40 (m, 1H), 7.32 (m, 2H), 7.20, (m, 2H), 4.73 (dd, J = 7.4, 3.7 Hz, 1H), 3.97 (m, 1H), 3.67 (m, 6H). $^{13}C\{^1H\}$ NMR (100.47 MHz, $CDCl_3$): δ 171.20, 138.93, 136.24, 131.05, 129.16, 129.05, 128.80, 128.18,

127.61, 87.44, 69.68, 65.96, 64.90. HRMS (EI) *m/z* calcd for $C_{17}H_{17}NO_2$ $(M)^+$ 267.1259, found 267.1256. X-ray crystal structure shown in Figure S21.

Table 3 entry 6b. *N*-(tetrahydrofuran-2-yl)-1,1-diphenylmethanimine.

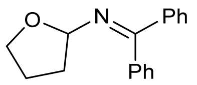

See representative reaction procedure on page S19. To 120.6 μL of $[Cl_2NN]Cu$ stock solution (2.4 mg, 1 mol% based on imine), benzophenone imine (82.7 μL, 0.493 mmol) tetrahydrofuran (0.40 mL, 4.93 mmol), ^{t}BuOO^{t}Bu (108.8 μL, 0.592 mmol) were added. The title compound was isolated as a colorless solid in 68% yield following the general purification procedure A. ^{1}H NMR (400 MHz, $CDCl_3$): δ 7.64 (m, 2H), 7.45 (m, 3H), 7.37 (m, 1H), 7.32 (m, 2H), 7.19 (m, 2H), 5.18 (dd, J = 6.3, 3.8 Hz, 1H), 4.22 (dt, J = 8.0, 6.9 Hz, 1H), 3.90 (m, 1H), 2.15 (m, 1H), 1.97 (m, 1H), 1.84 (m, 2H). $^{13}C\{^1H\}$ NMR (100.47 MHz, $CDCl_3$): δ 167.04, 139.57, 136.82, 130.41, 128.94, 128.69, 128.55, 128.12, 128.06, 91.87, 68.06, 34.23, 25.5. *N*-(tetrahydrofuran-2-yl)benzophenone imine was previously reported by Hartwig, J. F. *et al. ACS Cent. Sci.* 2016, **2**, 647-652.

Table 3 entry 6c. *N*-(1,3-dihydroisobenzofuran-1-yl)-1,1-diphenylmethanimine.

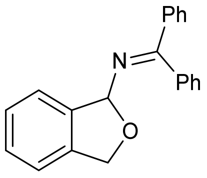

See representative reaction procedure on page S19. To 111.8 μL of $[Cl_2NN]Cu$ stock solution (2.2 mg, 1 mol% based on imine), benzophenone imine (76.6 μL, 0.457 mmol) 1,3-dihydrobenzofuran (0.50 mL, 4.57 mmol), ^{t}BuOO^{t}Bu (100.8 μL, 0.548 mmol) were added. The title compound was isolated as a off-white solid in 46% yield following the general purification procedure A. ^{1}H NMR (400 MHz, $CDCl_3$): δ 7.66 (m, 2H), 7.50 (m, 3H), 7.39 (m, 3H), 7.29 (m, 6H), 7.12 (d, J = 7.4 Hz,1H), 6.43 (s, 1H), 5.46 (d, J = 12.2 Hz, 1H), 5.12 (d, J = 12.2 Hz, 1H). $^{13}C\{^1H\}$ NMR (100.47 MHz, $CDCl_3$): δ 169.67, 140.27, 140.13, 139.51, 136.49, 130.75, 130.17, 129.33, 128.93, 128.76, 128.42, 128.37, 128.10, 127.52, 122.20, 121.31, 121.21, 95.24, 72.93. HRMS (CI) *m/z* calcd for $C_{21}H_{17}NO$ $(M+H)^+$ 300.1383, found 300.1384.

Table 3 entry 6d. *N*-(1,4,7,10-tetraoxacyclododecan-2-yl)-1,1-diphenylmethanimine. See

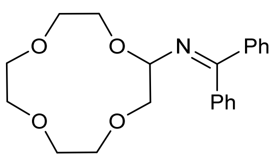

representative reaction procedure on page S19. To 75.6 μL of $[Cl_2NN]Cu$ stock solution (1.5 mg, 1 mol% based on imine), benzophenone imine (51.8 μL, 0.309 mmol) 12-crown-4 (0.50 mL, 3.09 mmol), ^{t}BuOO^{t}Bu (68.0 μL, 0.371 mmol) were added. The title compound was isolated as a colorless oil in 38% yield via column chromatography with neutral

alumina. The column was washed gradually with 5%, 25% and 80% diethylether in hexanes under low flow rate (18 mL/min) to remove left over 12-crown-4 starting material. The desired product elutes upon changing the second solvent from diethylether to ethyl acetate. ^{1}H NMR (400 MHz, $CDCl_3$): δ 7.66 (m, 2H), 7.44 (m, 4H), 7.33 (m, 2H), 7.16 (m, 2H), 5.02 (dd, J = 8.5 Hz, 2.7 Hz, 1H), 3.83 (m, 1H), 3.67 (m, 8H), 3.52 (m, 1H), 3.45 (m, 4H). ^{13}C{^{1}H} NMR (100.47 MHz, $CDCl_3$): δ 170.24, 138.98, 136.57, 130.85, 128.92, 128.88, 128.84 , 128.20, 127.50, 92.17, 73.01, 70.73, 70.68, 70.57, 70.06, 69.56, 68.55. HRMS (EI) *m/z* calcd for $C_{21}H_{25}NO_4$ $(M+Na)^+$ 378.1676, found 378.1675.

Table 3 entry 6e. *N*-(1,3,3-trimethyl-2-oxabicyclo[2.2.2]octan-5-yl)-1,1-diphenylmethanimine.

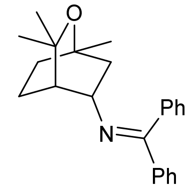

See representative reaction procedure on page S19. To 73.0 μL of $[Cl_2NN]Cu$ stock solution (1.5 mg, 1 mol% based on imine), benzophenone imine (50.0 μL, 0.299 mmol) eucalyptol (0.50 mL, 2.99 mmol), ^{t}BuOO^{t}Bu (65.8 μL, 0.358 mmol) were added. The title compound was isolated as a off-white solid in 32% yield following the general purification procedure A. The compound was recrystallized from EtOAc to get X-ray quality crystals. ^{1}H NMR (400 MHz, $CDCl_3$): δ 7.65 (m, 2H), 7.43 (m, 3H), 7.34 (m, 3H), 7.13 (m, 2H), 4.02 (m, 1H), 2.58 (m, 1H), 1.70 (m, 4H), 1.49 (dd, J = 13.4, 3.9 Hz, 1H), 1.31 (m, 1H), 1.25 (s, 3H), 1.07 (s, 3H), 0.91 (s, 3H). ^{13}C{^{1}H} NMR (100.47 MHz, $CDCl_3$): δ 166.51, 140.25, 138.30, 129.92, 128.54, 128.36 , 128.17, 127.68, 73.75, 70.86, 54.99, 40.97, 40.36, 32.01, 29.10, 28.16, 27.43, 16.01. HRMS (CI) *m/z* calcd for $C_{23}H_{27}NO$ $(M+H)^+$ 334.2165, found 334.2163. X-ray crystal structure shown in Figure S22.

Table 3 entry 6f. *N*-(1-(pyridin-2-yl)ethyl)-1,1-diphenylmethanimine.

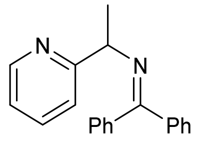

See representative reaction procedure in page S19. To 107 μL of $[Cl_2NN]Cu$ stock solution (2.1 mg, 1 mol% based on imine), benzophenone imine (73.2 μL, 0.437 mmol) 2-ethylpyridine (0.50 mL, 4.37 mmol), ^{t}BuOO^{t}Bu (96.4 μL, 0.525 mmol) were added. The title compound was isolated as a yellow oil in 19% yield following the general purification procedure A. ^{1}H NMR (400 MHz, $CDCl_3$): δ 8.50 (m, 1H), 7.72 (m, 2H), 7.67 (m, 2H), 7.38 (m, 7H), 7.15 (m, 2H), 4.71 (q, J = 6.6 Hz, 1H), 1.51 (d, J = 6.6 Hz, 3H). ^{13}C{^{1}H} NMR (100.47 MHz, $CDCl_3$): δ 167.41, 165.05, 148.98, 140.11, 136.80, 136.67, 130.16, 128.74, 128.70, 128.57, 128.19, 127.76, 121.79, 121.34, 63.29, 23.90. HRMS (CI) *m/z* calcd for $C_{20}H_{18}N_2$ $(M+H)^+$ 287.1543, found 287.1546.

Table 3 entry 6g. *N*-(1-(thiophen-2-yl)ethyl)-1,1-diphenylmethanimine. See representative reaction procedure on page S19. To 108.0 µL of [Cl_2NN]Cu stock solution (2.2 mg, 1 mol% based on imine), benzophenone imine (74.0 µL, 0.441 mmol) 2-ethylthiophene (0.50 mL, 4.41 mmol), ^{t}BuOO^{t}Bu (97.2 µL, 0.529 mmol) were added. The title compound was isolated as a yellow oil in 29% yield following the general purification procedure B. ^{1}H NMR (400 MHz, $CDCl_3$): δ 7.68 (m, 2H), 7.46 (m, 3H), 7.35 (m, 3H), 7.19 (m, 3H), 6.94 (dd, J = 5.1, 3.5 Hz, 1H), 6.81 (m, 1H), 4.82 (q, J = 6.4 Hz, 1H), 1.55 (d, J = 6.4 Hz, 3H). ^{13}C{^{1}H} NMR (100.47 MHz, $CDCl_3$): δ 167.01, 150.24, 139.83, 136.69, 130.22, 128.82, 128.67, 128.61, 128.18, 127.74, 126.55, 123.73, 122.14, 57.62, 25.42. HRMS (CI) *m/z* calcd for $C_{19}H_{18}NS$ $(M+H)^+$ 292.1154, found 292.1157.

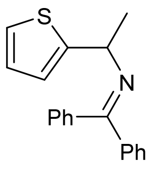

Table 3 entry 6h. 1-phenylethan-1-amine hydrochloride. See representative reaction procedure in page S19. The title compound was isolated as a colorless solid in 42% yield following general purification procedure C. ^{1}H NMR (400 MHz, D_2O): δ 7.48 (m, 5H), 4.54 (q, J = 7.0 Hz, 1H), 1.64 (d, J = 6.8 Hz, 3H). ^{13}C{^{1}H} NMR (100.47 MHz, D_2O): δ 137.62, 129.13, 129.04, 126.39, 50.89, 19.17. 1-phenylethan-1-amine hydrochloride was previously reported by Zhang, X. *et al. Angew. Chem. Int. Ed.* 2014, **53**, 8467–8470.

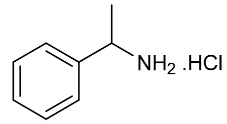

Table 3 entry 6i. 1-(4-chlorophenyl)ethan-1-amine hydrochloride. See representative reaction procedure on page S19. To 91.0 µL of [Cl_2NN]Cu stock solution (1.8 mg, 1 mol% based on imine), benzophenone imine (62.2 µL, 0.372 mmol) 4-chloroethylbenzene (0.50 mL, 3.72 mmol), ^{t}BuOO^{t}Bu (82.0 µL, 0.446 mmol) were added. The title compound was isolated as a colorless solid in 40% yield following general purification procedure C. The sticky solid was washed with diethyl ether to get the final crystalline product. ^{1}H NMR (400 MHz, D_2O): δ 7.50 (d, J = 8.0 Hz, 2H), 7.44 (d, J = 8.3 Hz, 2H), 4.54 (q, J = 6.8 Hz, 1H), 1.64 (d, J = 6.8 Hz, 3H). ^{13}C{^{1}H} NMR (100.47 MHz, D_2O): δ 136.15, 134.20, 129.05, 128.06, 50.26, 18.96. 1-(4-chlorophenyl)ethan-1-amine hydrochloride. was previously reported by Kramer, S. *et al. Org. Lett.* 2019, **21**, 65-69.

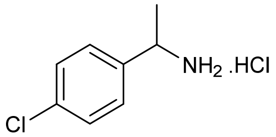

Table 3 entry 6j. 1-(naphthalen-2-yl)ethan-1-amine hydrochloride. See representative reaction procedure on page S19.

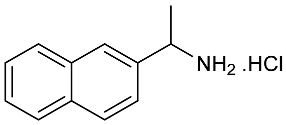

To 77.8 μL of [Cl_2NN]Cu stock solution (1.6 mg, 1 mol% based on imine), benzophenone imine (53.2 μL, 0.318 mmol) 2-ethylnaphthalene (0.50 mL, 3.18 mmol), ^{t}BuOO^{t}Bu (70.0 μL, 0.381 mmol) were added. The title compound was isolated as a colorless solid in 42% yield following general purification procedure C. ^{1}H NMR (400 MHz, D_2O): δ 8.05 (d, J = 8.5 Hz, 1H), 8.00 (m, 2H), 7.63 (m, 3H), 4.74 (q, J = 7.0 Hz, IH), 1.77 (d, J = 7.0 Hz, 3H). ^{13}C{^{1}H} NMR (100.47 MHz, D_2O): δ 135.20, 132.95, 132.81, 129.04, 128.00, 127.66, 127.02, 126.94, 125.77, 123.86, 51.07, 19.25. 1-(Naphthalen-2-yl)ethan-1-amine hydrochloride was previously reported by Zhang, X. *et al. Angew. Chem. Int. Ed.* 2014, **53**, 8467–8470.

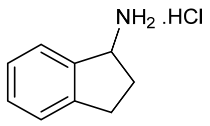

Table 3 entry 6k. 2,3-dihydro-1H-inden-1-amine hydrochloride. See representative reaction procedure on page S19. To 100.0 μL of [Cl_2NN]Cu stock solution (2.0 mg, 1 mol% based on imine), benzophenone imine (68.5 μL, 0.408 mmol) indane (0.50 mL, 4.08 mmol), ^{t}BuOO^{t}Bu (90.0 μL, 0.490 mmol) were added. The title compound was isolated as a colorless solid in 51% yield following general purification procedure C. ^{1}H NMR (400 MHz, D_2O): δ 7.41 (d, J = 7.5 Hz, 1H), 7.50 (m, 2H), 7.35 (m, 1H), 4.86 (dd, J = 7.9, 4.9 Hz, 1H), 3.14 (ddd, J = 16.8, 8.4, 6.1 Hz, 1H), 2.99 (ddd, J = 16.6, 9.0, 5.5 Hz, 1H), 2.60 (m, 1H), 2.12 (m, 1H). ^{13}C{^{1}H} NMR (100.47 MHz, D_2O): δ 144.34, 137.89, 129.54, 127.00, 125.26, 124.26, 55.57, 29.94, 29.52. 2,3-dihydro-1H-inden-1-amine hydrochloride was previously reported by Mecinovic *et al. J. Green Chem.* 2018, **20**, 4418–4433.

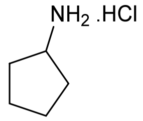

Table 3 entry 6l. cyclopentylamine hydrochloride. See representative reaction procedure on page S19. To 131.0 μL of [Cl_2NN]Cu stock solution (2.6 mg, 1 mol% based on imine), bis[(4-trifluoromethyl)phenyl]methanimine (170 mg, 0.535 mmol) cyclopentane (0.50 mL, 5.35 mmol), ^{t}BuOO^{t}Bu (118.0 μL, 0.643 mmol) were added. The title compound was isolated as colorless solid in 48% yield following general purification procedure C. ^{1}H NMR (400 MHz, D_2O): δ 3.65 (m, 1H), 2.05 (m, 2H), 1.75 (m, 2H), 1.64 (m, 4H). ^{13}C{^{1}H} NMR (100.47 MHz, D_2O): δ 52.08, 30.48, 23.46. Cyclopentylamine hydrochloride was previously reported by Zwierzak, A. *et al. Synthesis*, 1985, **2**, 202-204.

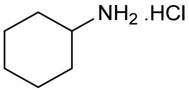

Table 3 entry 6m. cyclohexylamine hydrochloride. See representative reaction procedure on page S19. To 113.2 μL of [Cl_2NN]Cu stock solution

(2.3 mg, 1 mol% based on imine), bis[(4-trifluoromethyl)phenyl]methanimine (147 mg, 0.463 mmol) cyclohexane (0.50 mL, 4.63 mmol), ^{t}BuOO^{t}Bu (102.0 µL, 0.555 mmol) were added. The title compound was isolated as a colorless solid in 57% yield following general purification procedure C. ^{1}H NMR (400 MHz, D_2O): δ 3.17 (m, 1H), 2.01 (m, 2H), 1.81 (m, 2H), 1.68 (m, 1H), 1.36 (m, 4H), 1.20 (m, 1H). ^{13}C{^{1}H} NMR (100.47 MHz, D_2O): δ 50.28, 30.23, 24.19, 23.70. Cyclohexylamine hydrochloride was previously reported by Zwierzak, A. *et al. Synthesis,* 1985, **2**, 202-204.

Table 3 entry 6n. cyclooctylamine hydrochloride. See representative reaction procedure on page S19. To 91.0 µL of [Cl_2NN]Cu stock solution (1.8 mg, 1 mol% based on imine), bis[(4-trifluoromethyl)phenyl]methanimine (118 mg, 0.372 mmol) cyclooctane (0.50 mL, 3.72 mmol), ^{t}BuOO^{t}Bu (81.9 µL, 0.446 mmol) were added. The title compound was isolated as a colorless solid in 65% yield following general purification procedure C. ^{1}H NMR (400 MHz, D_2O): δ 3.44 (m, 1H), 1.90 (m, 2H), 1.74 (m, 4H), 1.56 (m, 8H). ^{13}C{^{1}H} NMR (100.47 MHz, D_2O): δ 51.93, 30.20, 25.94, 24.99, 22.88. Cyclooctylamine hydrochloride was previously reported by Issacs, L. *et al. Angew. Chem. Int. Ed.* 2014, **53**, 988-993.

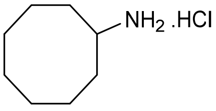

Table 3 entry 6o. 2-aminonorbornane hydrochloride. See representative reaction procedure on page S19. To 63.6 µL of [Cl_2NN]Cu stock solution (1.3 mg, 1 mol% based on imine), bis[(4-trifluoromethyl)phenyl]methanimine (82 mg, 0.260 mmol) norbornane (0.250 g, 2.60 mmol), ^{t}BuOO^{t}Bu (57.3 µL, 0.312 mmol) were added. The title compound was isolated as a colorless solid in 38% yield following general procedure C. ^{1}H NMR (400 MHz, D_2O): δ 3.21 (m, 1H), 2.37 (d, J = 4.9 Hz, 2H), 1.80 (m, 1H), 1.60 (m, 1H), 1.51 (m, 2H), 1.43 (m, 1H), 1.32 (m, 1H), 1.18 (m, 2H). ^{13}C{^{1}H} NMR (100.47 MHz, D_2O): δ 53.86, 40.24, 36.56, 35.79, 34.10, 26.91, 25.93. 2-aminonorbornane hydrochloride was reported by Scammells, P. J. *et al. Bioorgan. Med. Chem.,* 2002, **10**, 1115-1.

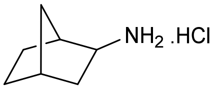

4. EPR Spectra of Cu(II) Intermediates

EPR spectra were collected on a JEOL continuous wave spectrometer JES-FA200 equipped with an X-band Gunn oscillator bridge, a cylindrical mode cavity, and a liquid nitrogen cryostat. EPR measurements were performed in sealed quartz tubes. All spectra were obtained on freshly prepared solutions (1 - 3 mM in toluene) of at least 2 independently synthesized batches and were checked carefully for reproducibility. Background spectra were obtained on clean solvents at the same measurement conditions. Spectral simulation was performed using the program QCMP 136 by Prof. Dr. Frank Neese from the Quantum Chemistry Program Exchange as used by Neese *et al.* in *J. Am. Chem. Soc.* **1996**, *118*, 8692-8699. The fittings were performed by the "chi by eye" approach and always taking all available spectra at different temperatures into account.

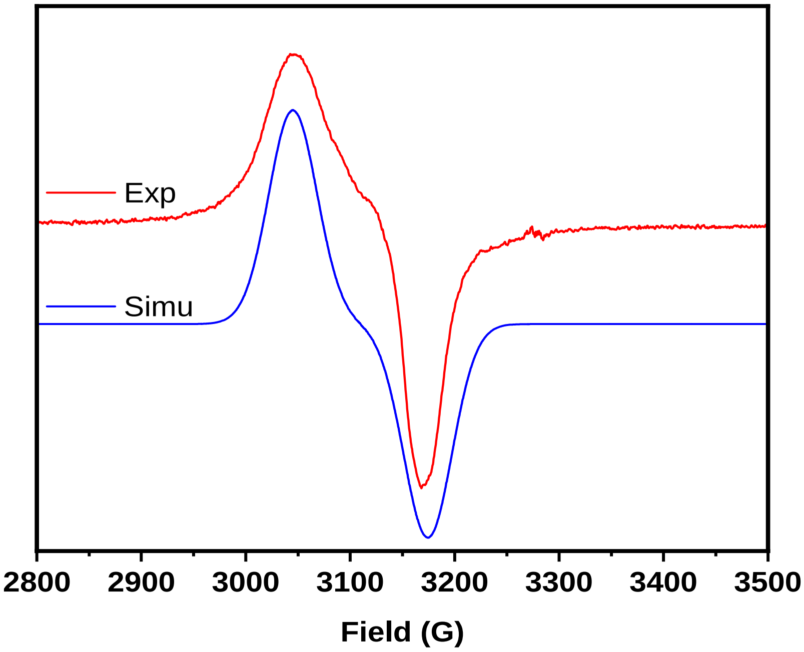

Figure S14. X-band EPR spectrum and simulation for [Me_3NN]Cu-O^{t}Bu (**2a**) (toluene, 298 K, 9.1791 GHz, Power = 1 mW, ModWidth = 40 mT, time constant = 0.01 s). Simulation was performed using a Cu g_{iso} = 2.1168 with A_{iso}(Cu) = 60.0 MHz Asio(2N) = 5.0. Lorentzian lineshape with linewidth of W_{iso} = 18.00 mT.

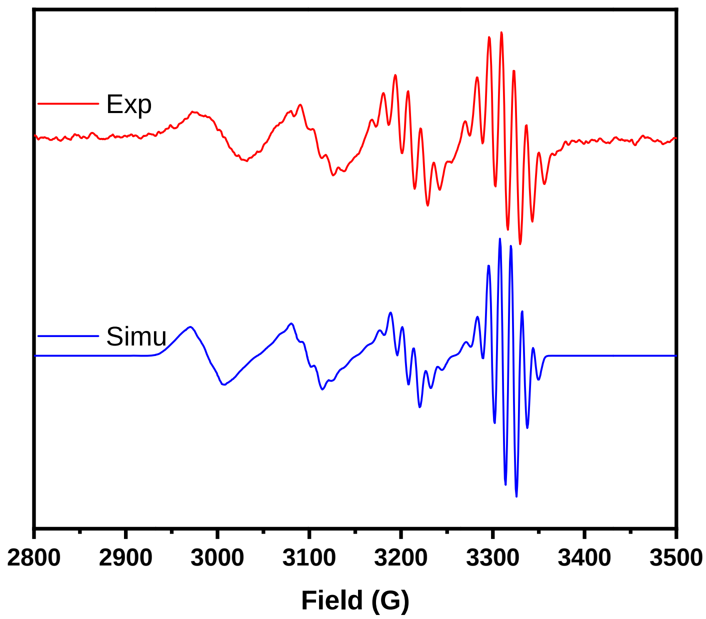

Figure S15. X-band EPR spectrum and simulation for [Me_3NN]Cu-N=CPh_2 (**3a**) (pentane and toluene, 298 K, 9.2032 GHz, Power = 1 mW, ModWidth = 1 mT, time constant = 0.01 s). Simulation was performed using a 1Cu, 3N model: g_{iso} = 2.0810 with A_{iso}(Cu) = 298.0 MHz and A_{iso}(3N) = 35.0 MHz. Lorentzian lineshape, with linewidth of W_{iso} = 6.00 mT.

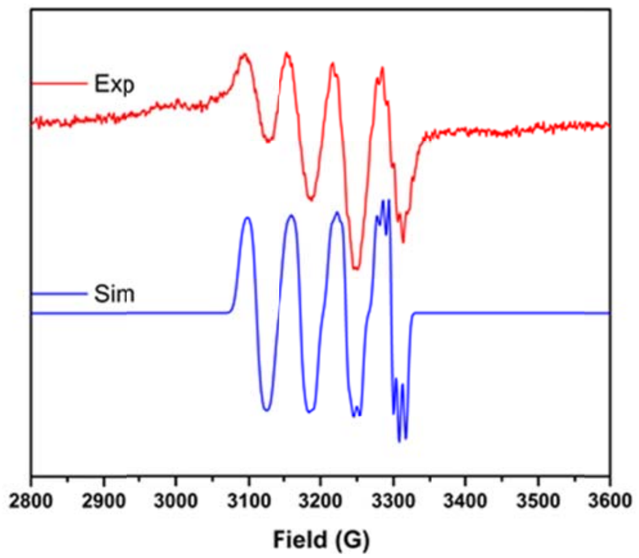

Figure S16. X-band EPR spectrum and simulation for [Cl_2NN]Cu-N=CPh_2 (**3b**) (pentane and toluene, 298 K, 8.9827 GHz, Power = 1 mW, ModWidth = 1 mT, time constant = 0.01 s). Simulation was performed using a 1Cu, 3N model: g_{iso} = 2.1030 with A_{iso}(Cu) = 205.0 MHz and A_{iso}(3N) = 27.0 MHz Lorentzian line shape, with linewidth of W_{iso} = 6.70 mT.

5. Crystallographic Data.

Single crystals of compounds [Me_3NN]Cu-O^{t}Bu (**2a**: CCDC 1940417), [Me_3NN]Cu-N=CPh_2 (**3a**: CCDC 1945374), [Me_3NN]Cu(NH=CPh_2) • THF (**4a•THF**: CCDC 1940418), [Cl_2NN]Cu(NH=CPh_2) (**4b**: CCDC 1945375), *N*-(1,4-dioxan-2-yl)-1,1-diphenylmethanimine (**6a**: CCDC 1940420), and *N*-(1,3,3-trimethyl-2-oxabicyclo[2.2.2]octan-5-yl)-1,1-diphenylmethanimine (**6e**: CCDC 2035780) were mounted under mineral oil on Mitegen micromounts and immediately placed in a cold nitrogen stream at 100(2) K prior to data collection. Data for compounds **2a**, **3a**, **4b**, and *N*-(1,4-dioxan-2-yl)-1,1-diphenylmethanimine were collected on a Bruker DUO equipped with an APEXII CCD detector and Mo fine-focus sealed source. Data for compound (**4a•THF**) and *N*-(1,3,3-trimethyl-2-oxabicyclo[2.2.2]octan-5-yl)-1,1-diphenylmethanimine were collected on a Bruker D8 Quest equipped with a Photon100 CMOS detector and a Mo ImS source. Hemispheres of data were collected (0.5°ω-scans; $2\theta_{max}$ = 56°; monochromatic Mo Kα radiation, λ = 0.7107 Å) and integrated with the Bruker SAINT program. Structure solutions were performed using the SHELXTL/PC suite.[12] Intensities were corrected for Lorentz and polarization effects and an empirical absorption correction was applied using Blessing's method as incorporated into the program SADABS.[13] Non-hydrogen atoms were refined with anisotropic thermal parameters and hydrogen atoms were included in idealized positions unless otherwise noted. Structures were rendered with POV-Ray in Mercury using 50% probability ellipsoids unless otherwise mentioned. Further comments on disorder models:

[Me_3NN]Cu-O^{t}Bu (2a): The *t*-butyl group is disordered over two orientations. The like O-C distances and C-C distances were restrained to be similar (esd 0.01 Å). Similar displacement amplitudes were imposed on disordered sites overlapping by less than the sum of van der Waals radii.

[Me_3NN]Cu(NH=CPh_2) •THF (4a•THF): The amine H atom was located in the difference map and its position was allowed to freely refine. The amine H atom U's were assigned as 1.5 times the U_{eq} of the N3. This H atom refined to good intermolecular hydrogen bonding position with the THF solvate molecule.

[Cl_2NN]Cu(NH=CPh_2) (4b): The amine H atom was located in the difference map and its position was allowed to freely refine. The amine H atom U's were assigned as 1.5 times the U_{eq} of the N3.

***N*-(1,3,3-trimethyl-2-oxabicyclo[2.2.2]octan-5-yl)-1,1-diphenylmethanimine (6e):** This crystal was refined as a two component twin. The relative occupancies of the two components refined to approximately 50:50. The twin law by rows relating domain two to domain one is (-1 0 0), (0 -1 0), (0.38 0 1). The absolute configuration of the chiral centers could not be determined by the X-ray structure.

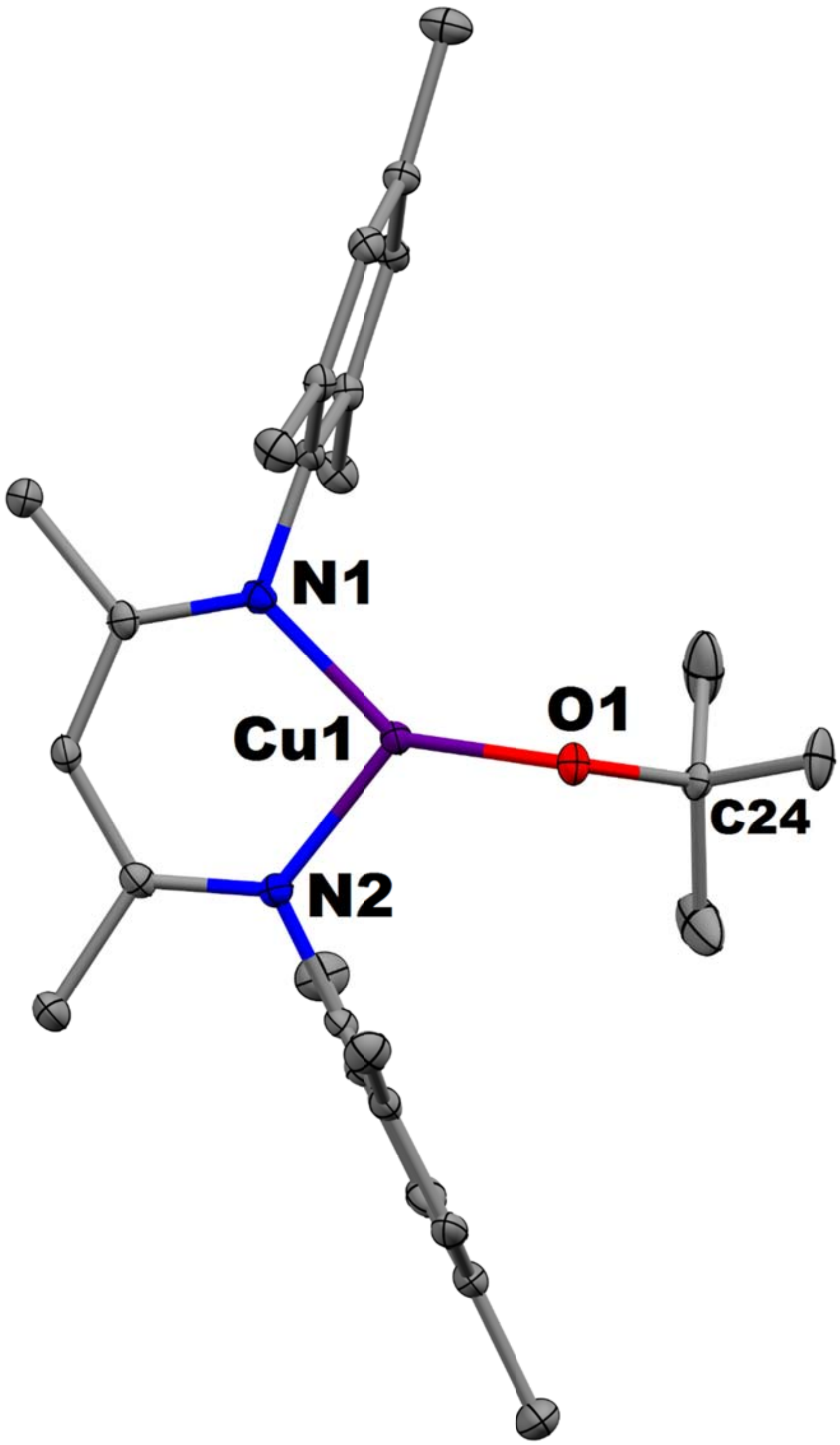

Figure S17. X-ray crystal structure of [Me_3NN]Cu-O^{t}Bu (**2a**: CCDC 1940417) at 100 K. Only the primary location for all disordered sites is shown. All carbon bound hydrogen atoms have been omitted for clarity. Cu1-O1 1.7860(16) Cu1-N1 1.884(2) Cu1-N2 1.894(2) O1-Cu1-N1 140.89(8) O1-Cu1-N2 121.82(8).

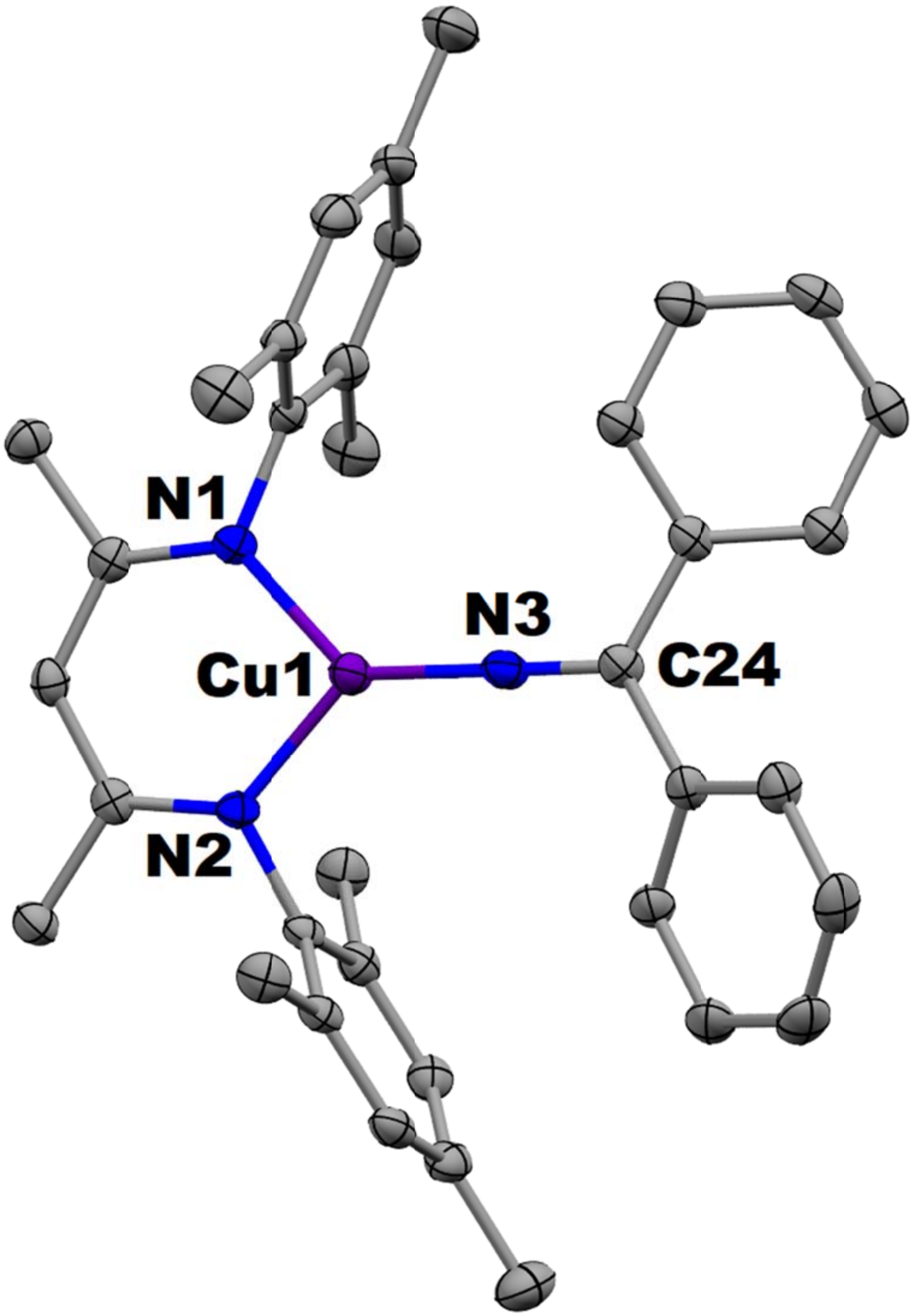

Figure S18. X-ray crystal structure of [Me_3NN]Cu-N=CPh_2 (**3a**: CCDC 1945374) at 100 K. All carbon bound hydrogen atoms have been omitted for clarity. Selected bond distances (Å) and angles (°): Cu1-N3 1.700(2) Cu1-N2 1.861(2) Cu1-N1 1.855(2) N3-C24 1.274(3) N3-Cu1-N1 132.69(10) N3-Cu1-N2 132.78(10), N3-Cu1-C24 178.9(2).

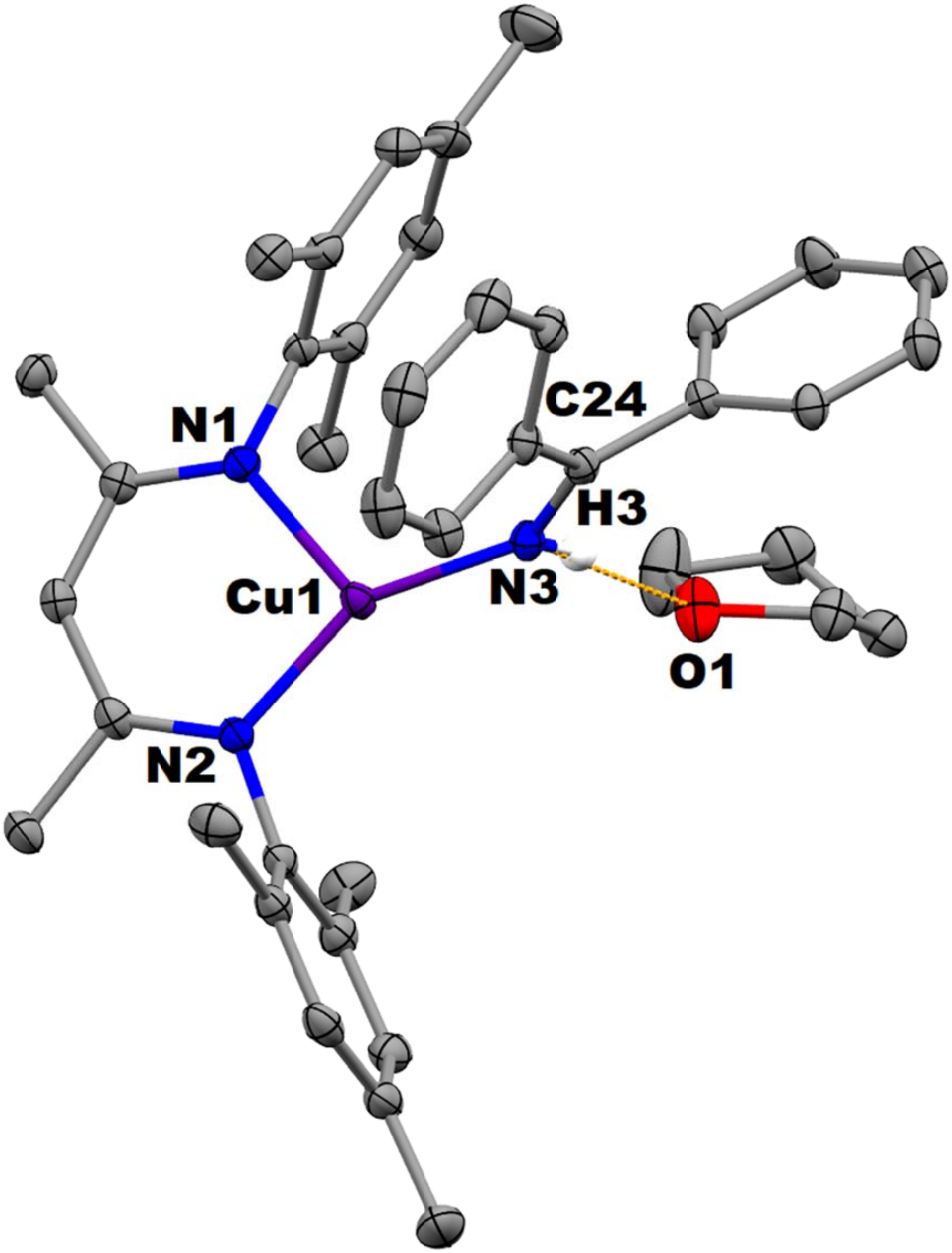

Figure S19. X-ray crystal structure of [Me_3NN]Cu(NH=CPh_2)•THF (**4a•THF**: CCDC 1940418) at 100 K. All carbon bound hydrogen atoms have been omitted for clarity. Selected bond distances (Å) and angles (°): Cu1-N1 1.9874 (14) Cu1-N2 1.1.9115 (14) Cu1-N3 (1.8940 (14) N1-C24 1.2922 (20) N2-Cu1-N3 151.02(6) N1-Cu1-N2 97.53(6) N1-Cu1-N3 111.32(6) Cu1-N3-C24 132.68(12).

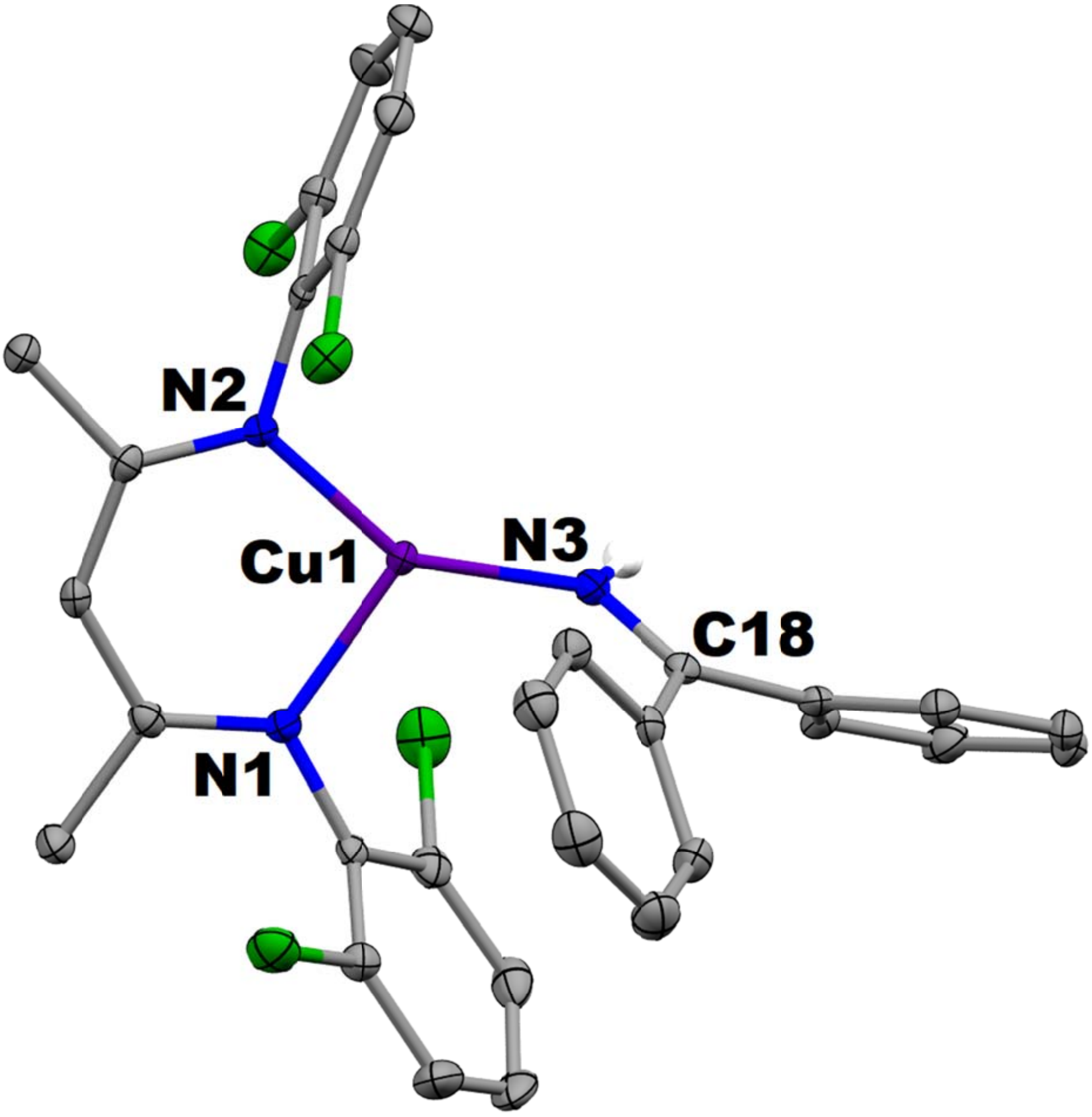

Figure S20. X-ray crystal structure of $[Cl_2NN]Cu(NH{=}CPh_2)$ (**4b**: CCDC 1945375) at 100 K. All carbon bound hydrogen atoms have been omitted for clarity. Selected bond distances (Å) and angles (°): Cu1-N11.9901(13) Cu1-N2 1.9049(13) Cu1-N3 1.8937(14) N1-C18 1.293(2) N2-Cu1-N3 147.58(6) N1-Cu1-N2 97.98(5) N1-Cu1-N3 114.37(6) Cu1-N3-C18 130.25(12).

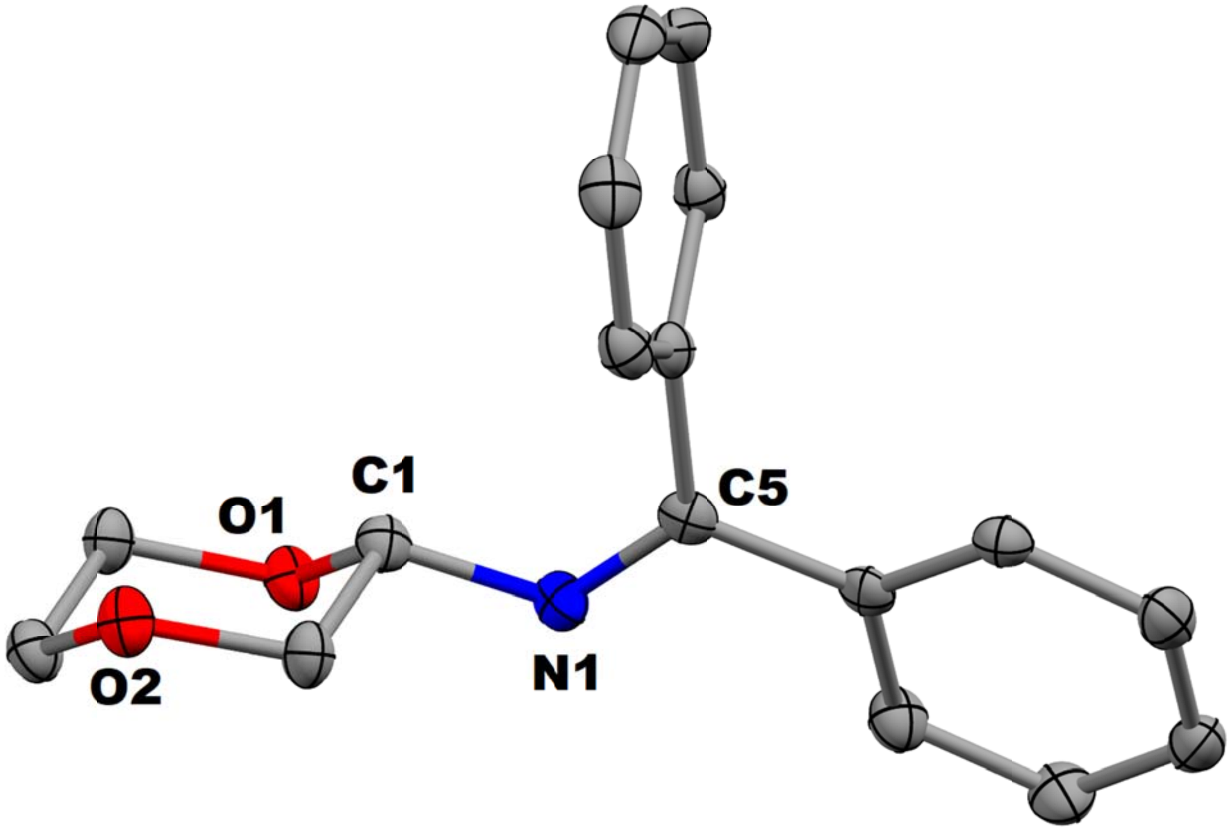

Figure S21. X-ray crystal structure of *N*-(1,4-dioxan-2-yl)-1,1-diphenylmethanimine (**6a**: CCDC 1940420) at 100 K. All carbon bound hydrogen atoms have been omitted.

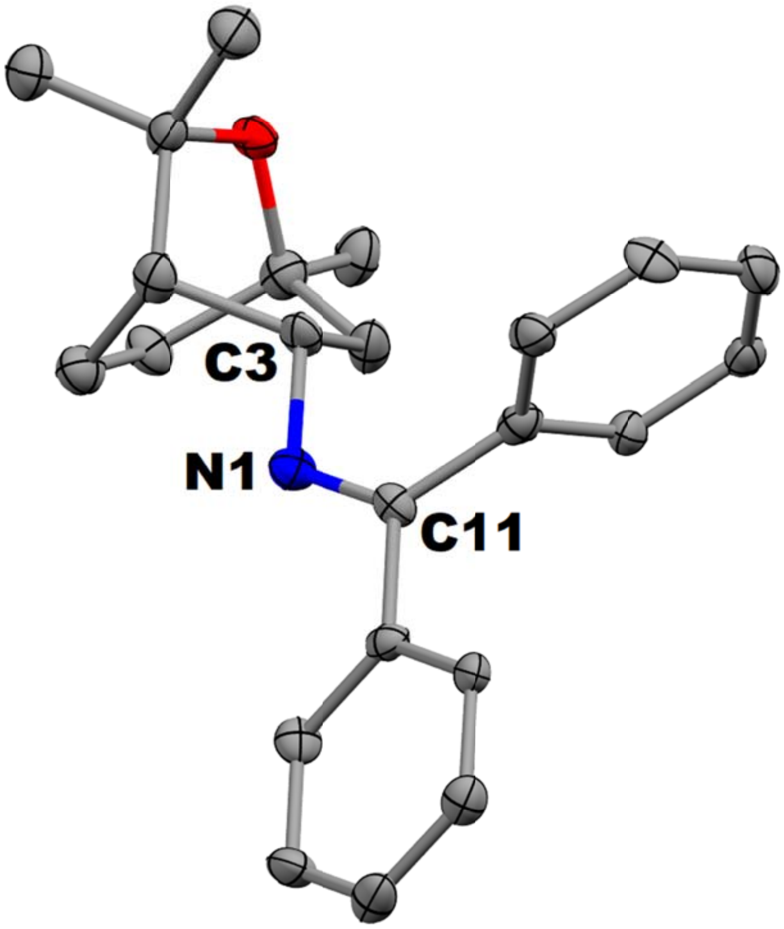

Figure S22. X-ray crystal structure of *N*-(1,3,3-trimethyl-2-oxabicyclo[2.2.2]octan-5-yl)-1,1-diphenylmethanimine (**6e**: CCDC 2035780) at 100 K. All carbon bound hydrogen atoms have been omitted.

6. References for Synthesis and Crystallographic Details

1. E. A. Haidasz, D. Meng, R. Amorati, A. Baschieri, K. U. Ingold, L. Valgimigli and D. A. Pratt, Acid is key to the radical-trapping antioxidant activity of nitroxides, *J. Am. Chem. Soc.*, 2016, **138**, 5290.
2. Y. M. Badiei, A. Dinescu ,X. Dai, R. M. Palomino, F. W. Heinemann, T. R. Cundari and T. H Warren, Copper–nitrene complexes in catalytic C-H amination, *Angew. Chem. Int. Ed.*, 2008, **47**, 9961-9964.
3. Y. M. Badiei and T. H. Warren, Electronic structure and electrophilic reactivity of discrete copper diphenyl carbenes, *Organometal. Chem.*, 2005, **690**, 5989-6000.
4. A. Bakhoda, O. E. Okoromoba, C. Green, M. R. Boroujeni, J. A. Bertke and T. H Warren, A three coordinated Cu(II) alkynyl complex in C-C bond formation: the sesquicentennial of the Glaser coupling, *J. Am, Chem. Soc.,* 2020, **142**, 18483-18490.
5. T. K Salvador, C. H Arnett, S. Kundu, N. Sapiezynski, J. A. Bertke, M. R. Boroujeni and T. H. Warren, Copper catalyzed sp^3 C-H etherification with acyl protected phenols, *J. Am. Chem. Soc.,* 2016, **138**, 16580-16583.
6. S. Wiese, Y. M, Badiei, R. T. Gephart, S.Mossin, M. S. Varonka, M. M. Melzer, K. Meyer, T. R. Cundari, and T. H. Warren, Catalytic C-H Amination with Unactivated Amines through Copper(II) Amides, *Angew. Chem. Int. Ed.*, 2010, **49**, 8850-8855.
7. A. Laouiti, M. M. Rammah, M. B. Rammah, J. Marrot, F. Couty and G. Evano, Copper-catalyzed oxidative alkynylation of diaryl imines with terminal alkynes: A facile synthesis of ynimines, *Org. Lett.,* 2012, **14**, 6-9
8. *US Pat.,* US20110071310A1, 2011.
9. R. He, Z. Huang, Q. Zheng, and C. Wang, Manganese-catalyzed dehydrogenative [4+2] annulation of N-H imines and alkynes by C-H/N-H activation, *Angew. Chem. Int. Ed.*, 2014, **53**, 4950-4953.
10. *US Pat.,* US2007055698A1, 2007.
11. S. Kramer, Syntehsis of α-substituted primary benzylamines through copper-catalyzed cross-dehydrogenative coupling, *Org. Lett.*, 2019, **21**, 65–69.
12. SHELXTL-PC, Vers. 5.10; 1998, Bruker-Analytical X-ray Services, Madison, WI; G. M. Sheldrick, SHELX-97, Universität Göttingen, Göttingen, Germany.

13. SADABS; G. M. Sheldrick, 1996, based on the method described in R. H. Blessing, *Acta Crystallogr. Sect. A*, 1995, **51**, 33.

7. Computational Methods and Results

Gaussian 16 was used to optimize the structures, calculate single point geometries, spin densities and reaction free energies. Visualization and structural analyses were done using Chemcraft 1.6. Two complexes were studied: [Me_3NN]Cu-N=CPh_2 (**3a**), [Cl_2NN]Cu-N=CPh_2 (**3b**),

All geometry optimizations were done using BP86 density functional in conjunction with 6-311+G(d) basis set. At the BP86/6−31+G(d) stationary points, single point energies were calculated using BP86 density functional with 6−311++G(d,p) basis set, in toluene using the implicit SMD solvent model and adding dispersion corrections with the keyword, empirical dispersion = GD3BJ.

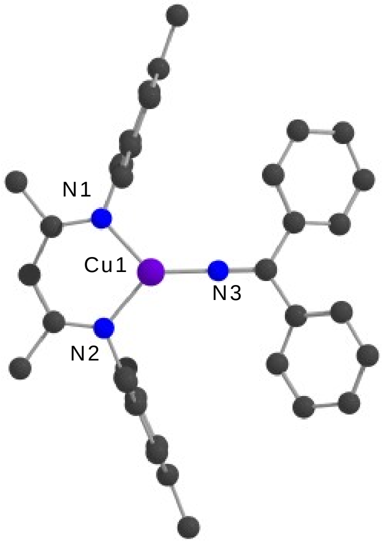

Figure S23. Geometry optimized structure of [Me_3NN]Cu-N=CPh_2 (**3a**) at BP86/6−311+G(d) level of theory with charge = 0, multiplicity = 2. Selected calculated bond distances (Å) and angles (°): Cu1-N1 1.9451, Cu1-N2 1.9451, Cu1-N3 1.7941, N1-Cu1-N3 131.37, N2-Cu1-N3 131.36, N1-Cu1-N2 97.27, Cu1-N3-C24 179.99.

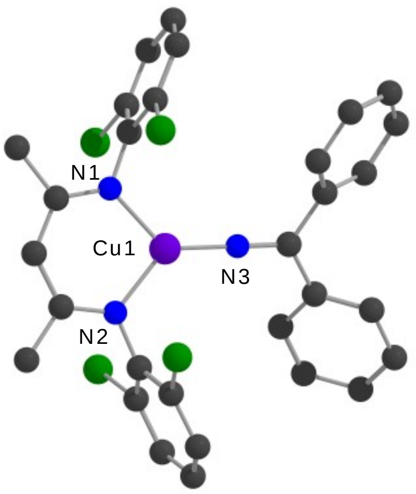

Figure S24. Geometry optimized structure of $[Cl_2NN]Cu\text{-}N{=}CPh_2$ (**3b**) at BP86/6−311+G(d) level of theory with charge = 0, multiplicity = 2. Selected calculated bond distances (Å) and angles (°): Cu1-N1 1.9470, Cu1-N2 1.9470, Cu1-N3 1.7906, N1-Cu1-N3 131.72, N2-Cu1-N3 131.72, N1-Cu1-N2 96.56, Cu1-N3-C24 179.99.

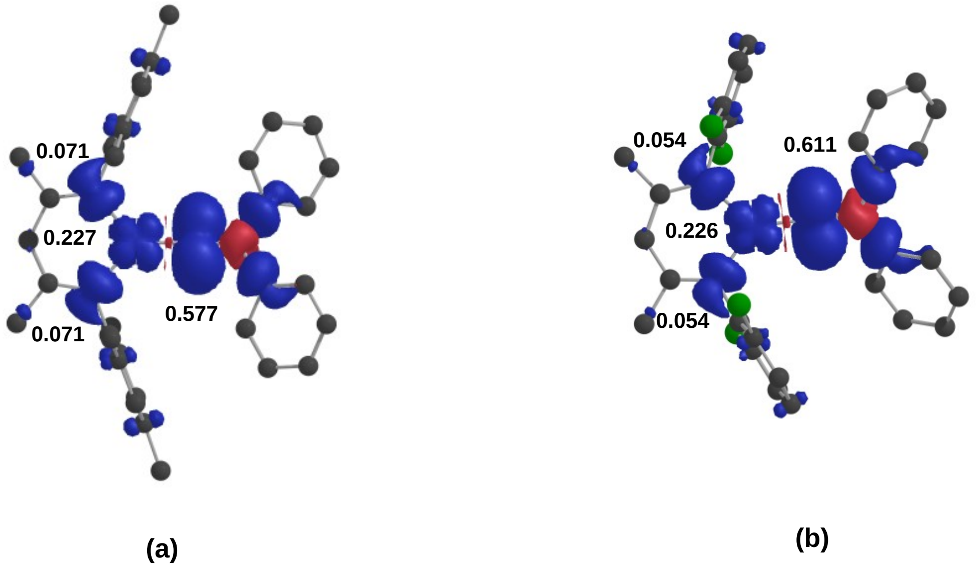

Figure S25. Spin density plot of (a) $[Me_3NN]Cu\text{-}N{=}CPh_2$ (**3a**) and (b) $[Cl_2NN]Cu\text{-}N{=}CPh_2$ (**3b**) with excess spin α (blue), 0.01 isospin value. Values shown corresponds to spin density on each atom.

8. NMR Spectra.

* *Residual proteo impurities in deuterated solvents.*

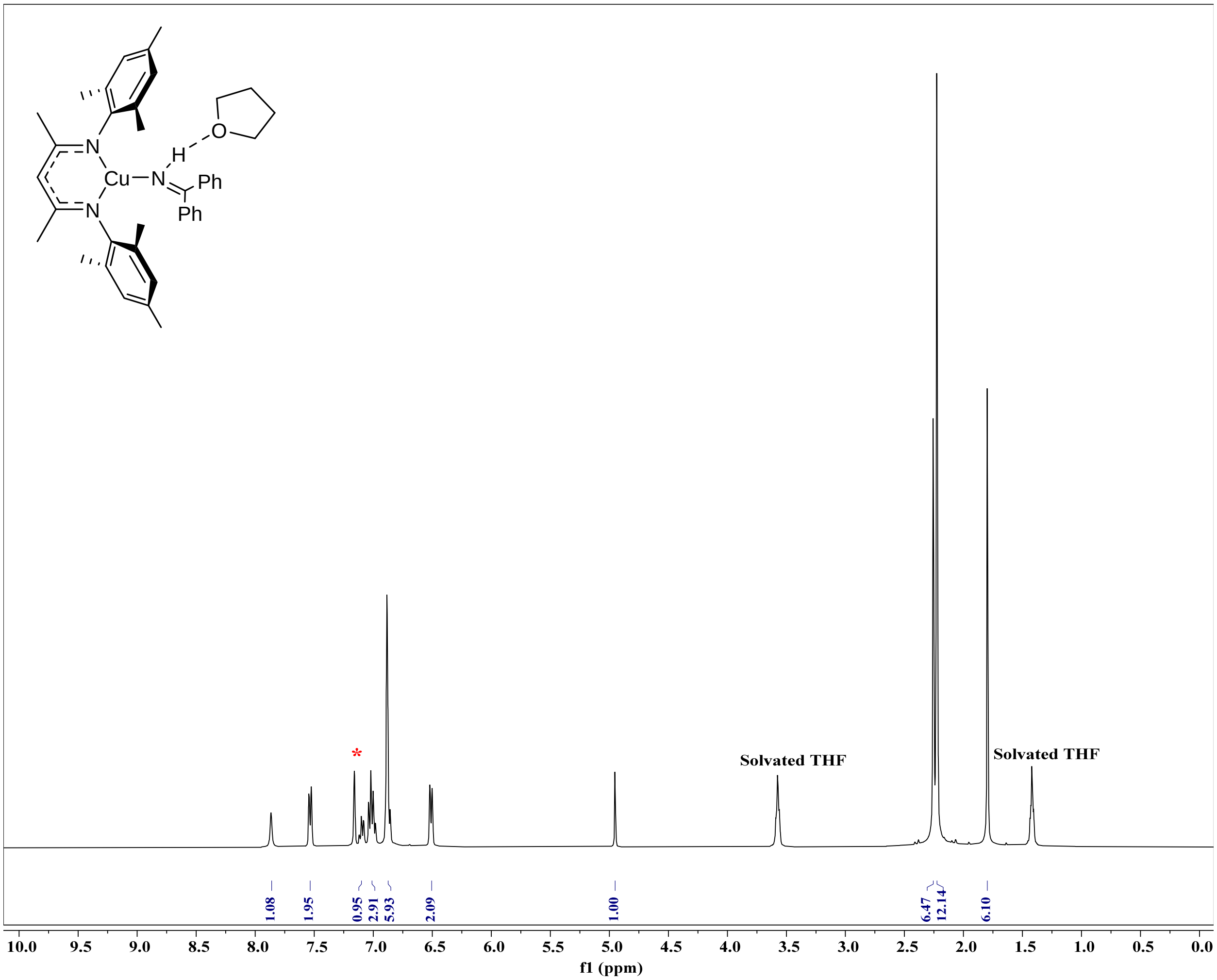

Figure S26. ^{1}H NMR spectrum (400 MHz, C_6D_6) of [Me_3NN]Cu(NH=CPh_2)•THF (**4a•THF**).

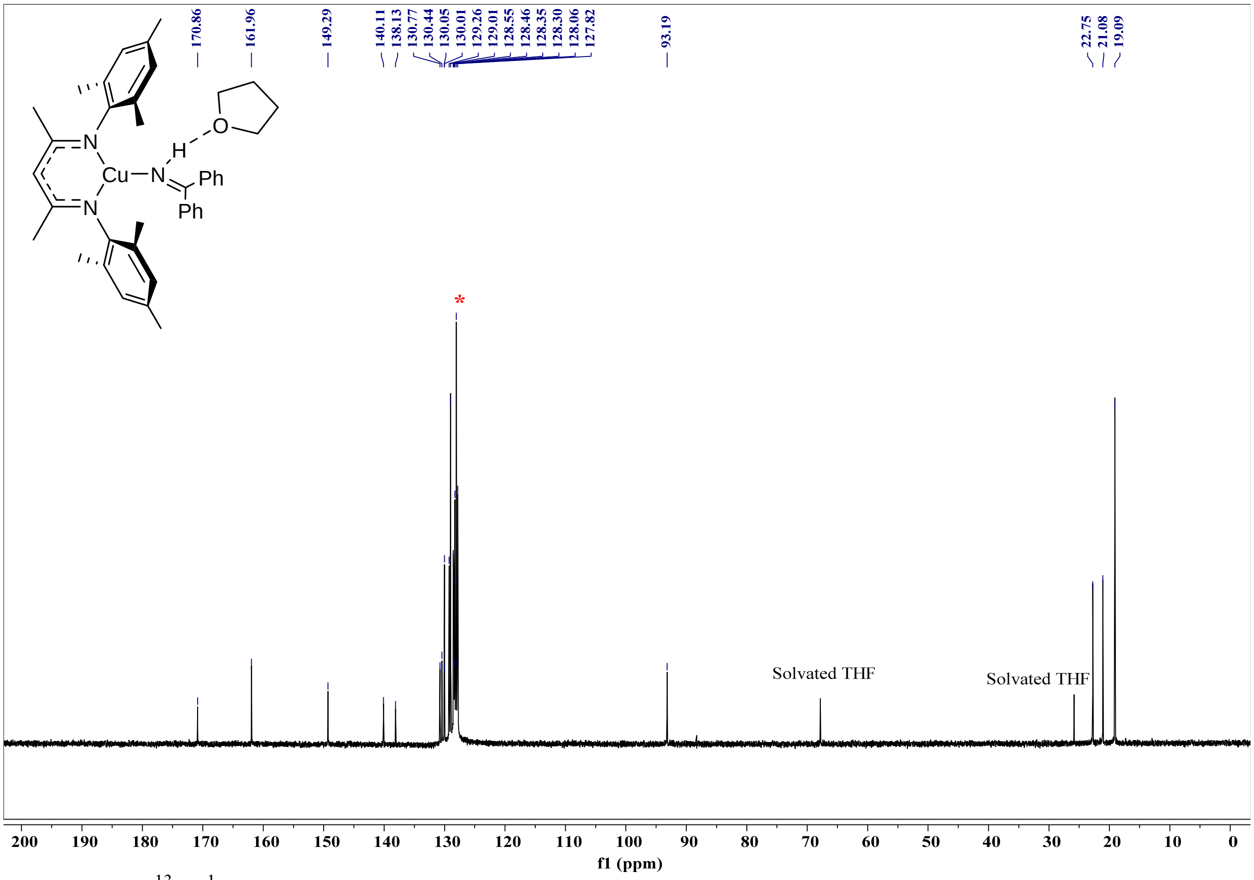

Figure S27. $^{13}C\{^{1}H\}$ NMR spectrum (100.47 MHz, C_6D_6) of $[Me_3NN]Cu\text{-}NH{=}CPh_2.THF$ (**4a•THF**).

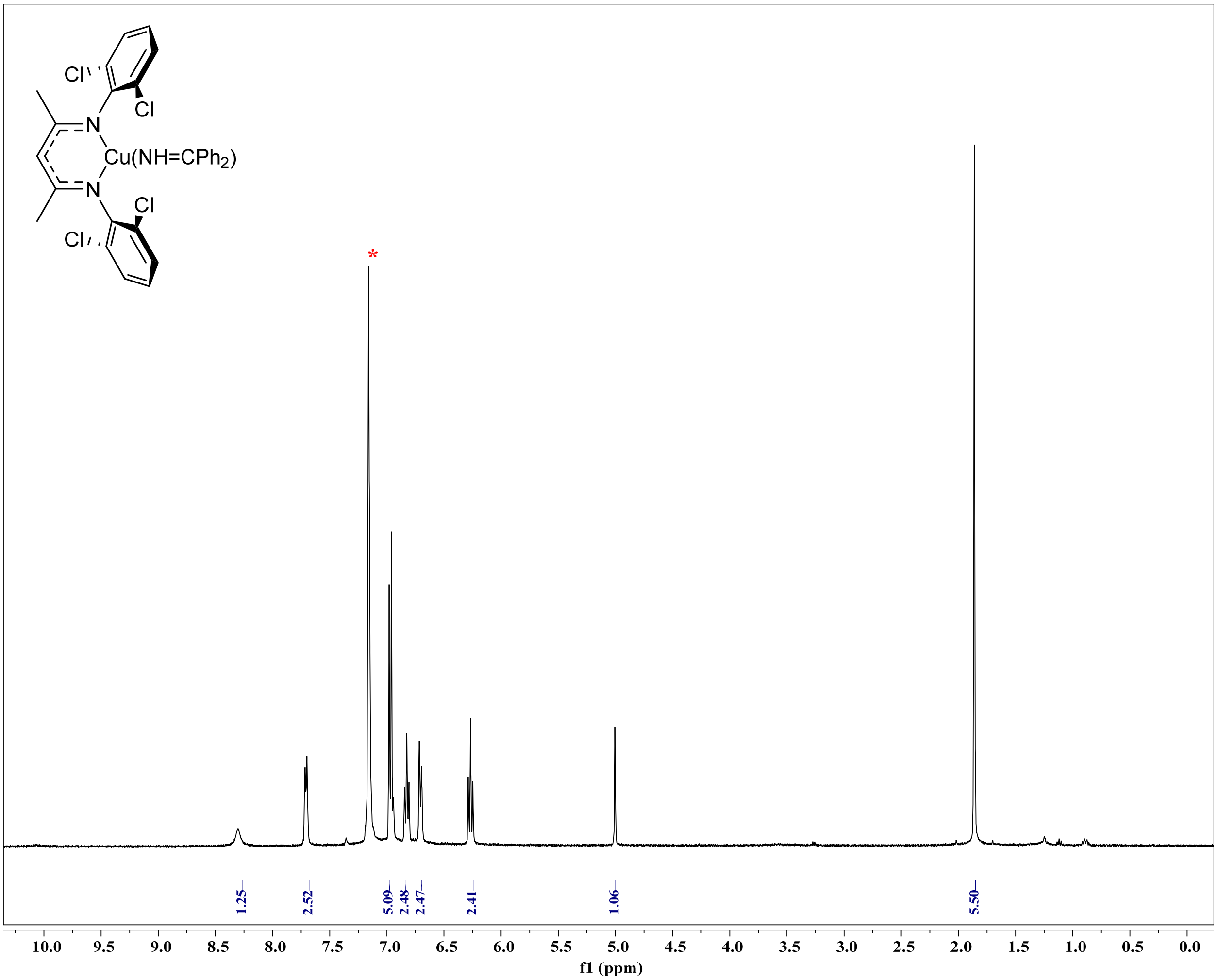

Figure S28. ^{1}H NMR spectrum (400 MHz, C_6D_6) of $[Cl_2NN]Cu\text{-}NH{=}CPh_2$ (**4b**).

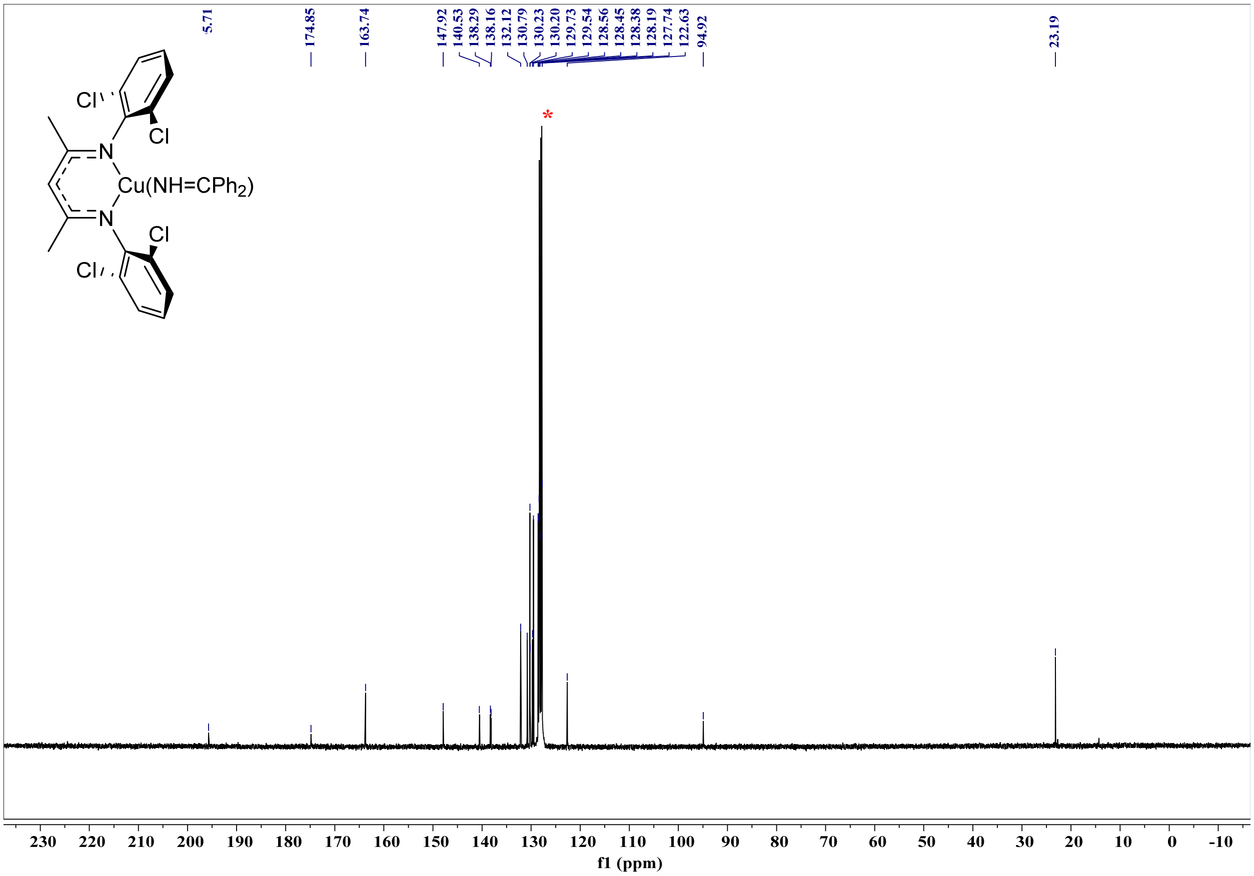

Figure S29. $^{13}C\{^{1}H\}$ NMR spectrum (100.47 MHz, C_6D_6) of $[Cl_2NN]Cu\text{-}NH{=}CPh_2$ (**4b**).

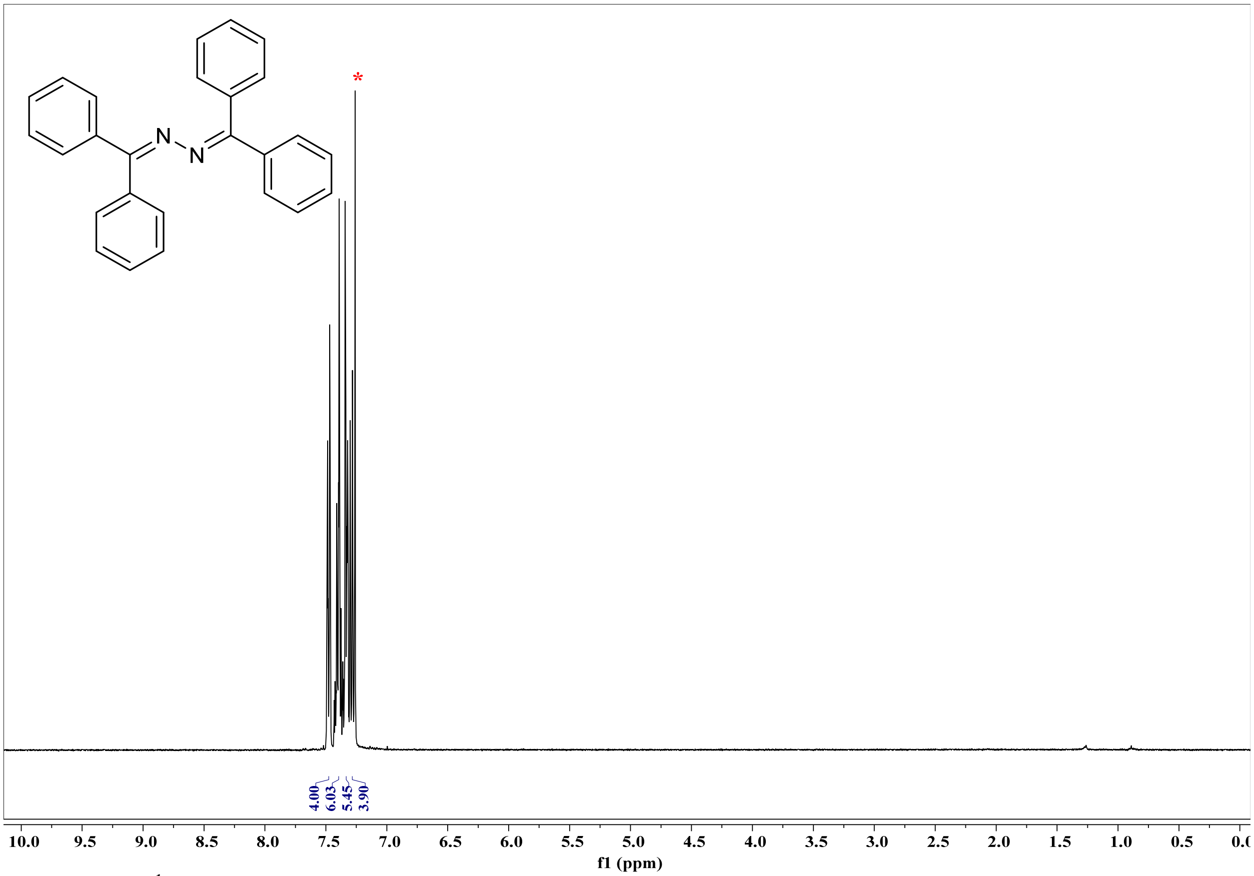

Figure S30. ^{1}H NMR spectrum (400 MHz, $CDCl_3$) of benzophenone azine.

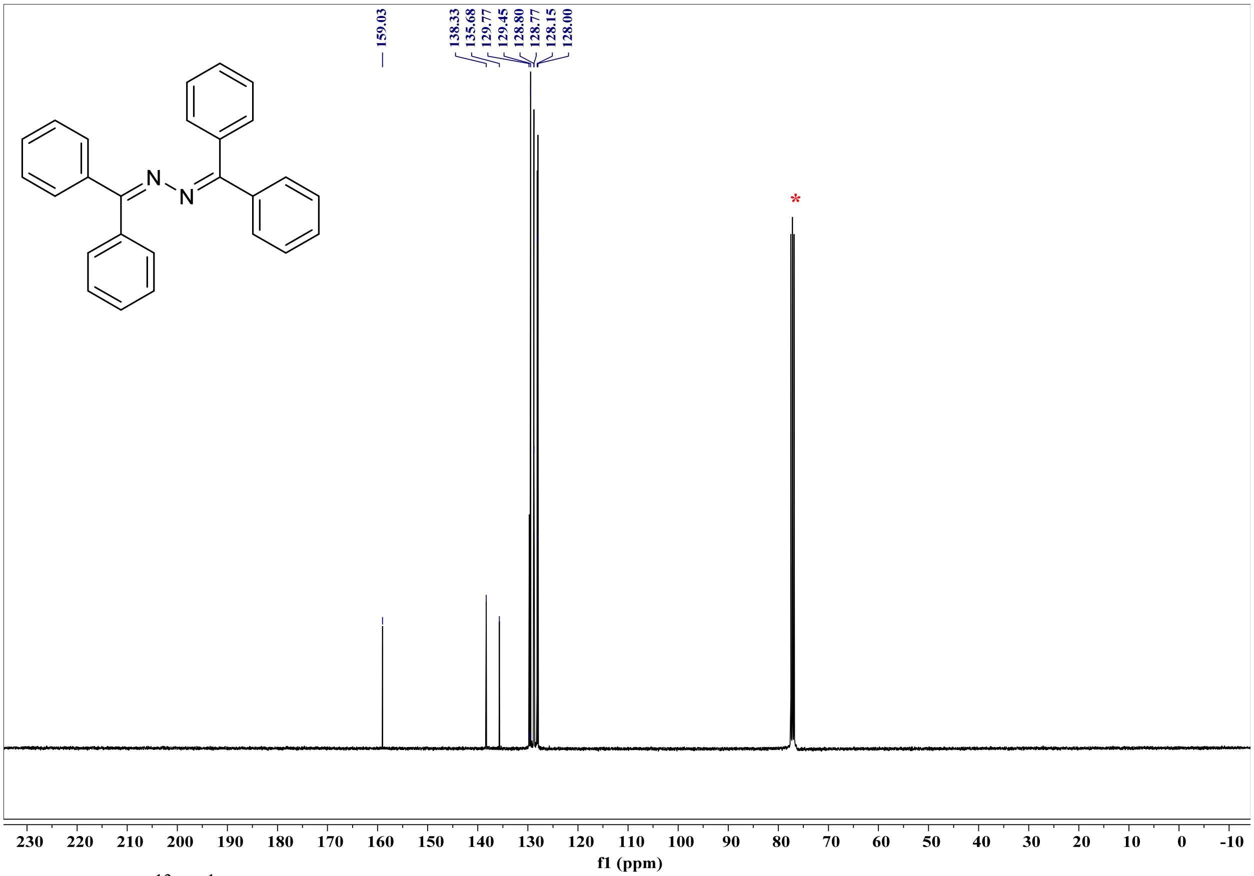

Figure S31. $^{13}C\{^{1}H\}$ NMR spectrum (100.47 MHz, $CDCl_3$) of benzophenone azine.

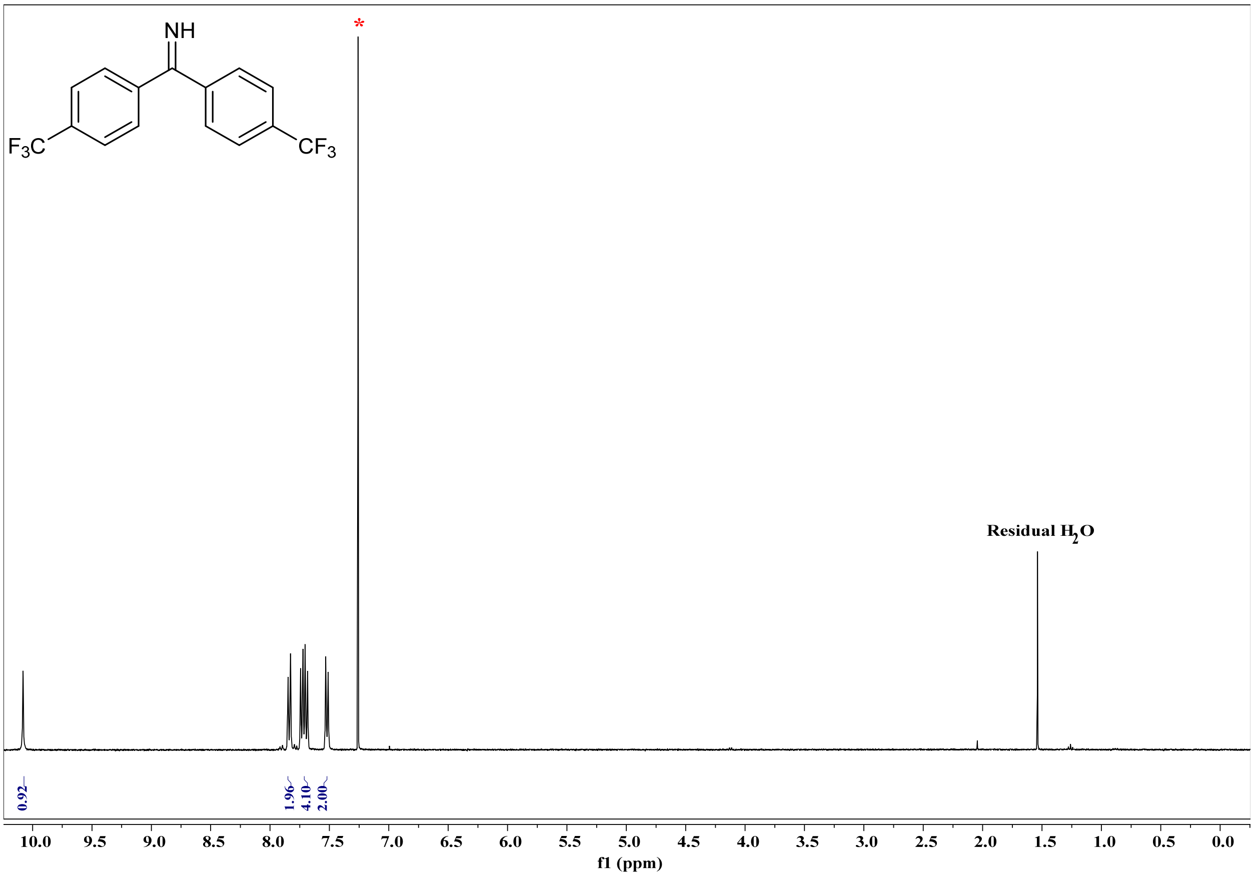

Figure S32. ^{1}H NMR spectrum (400 MHz, $CDCl_3$) of bis[4-(trifluoromethyl)phenyl]methanimine.

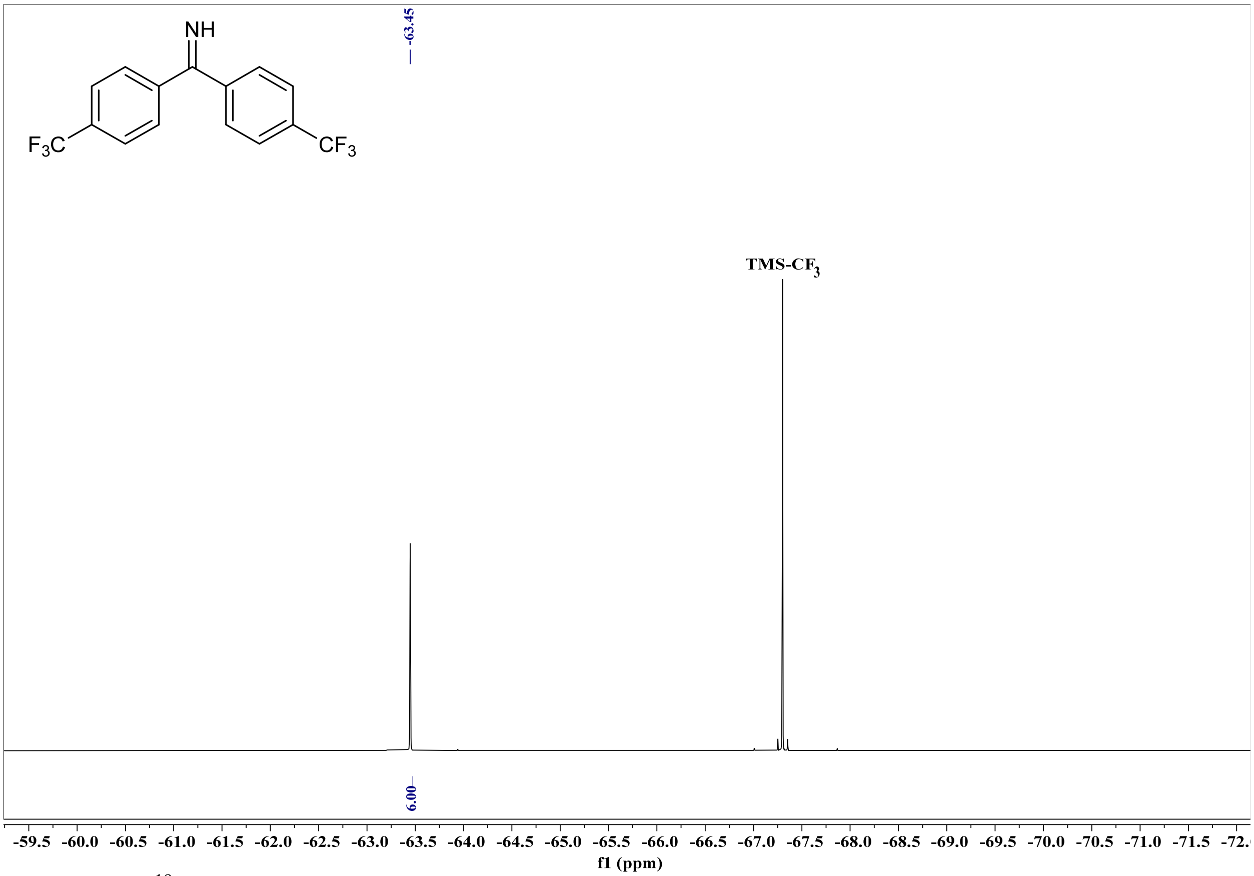

Figure S33. ^{19}F NMR spectrum (376 MHz, $CDCl_3$) of bis[4-(trifluoromethyl)phenyl]methanimine.

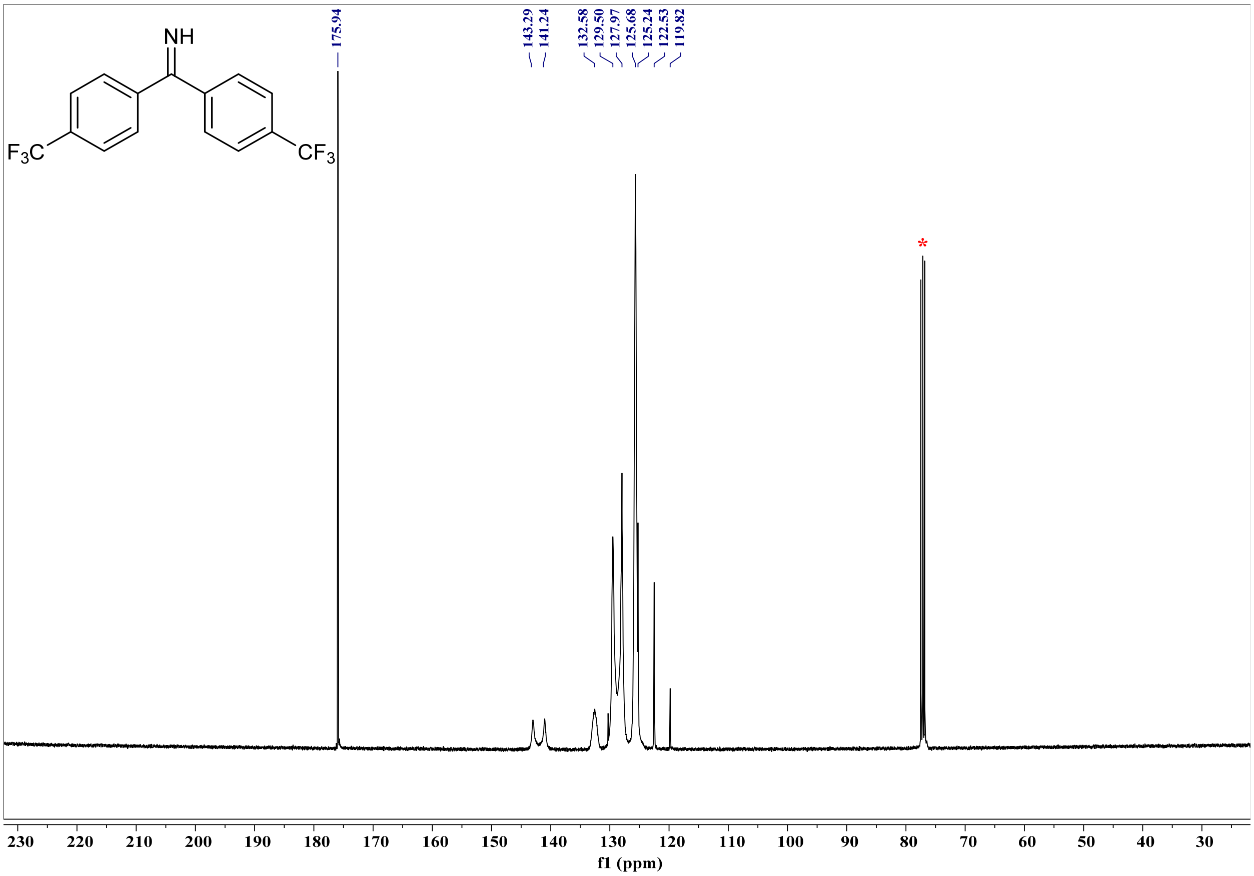

Figure S34. $^{13}C\{^{1}H\}$ NMR spectrum (100.47 MHz, $CDCl_3$) of bis[4-(trifluoromethyl)phenyl]methanimine.

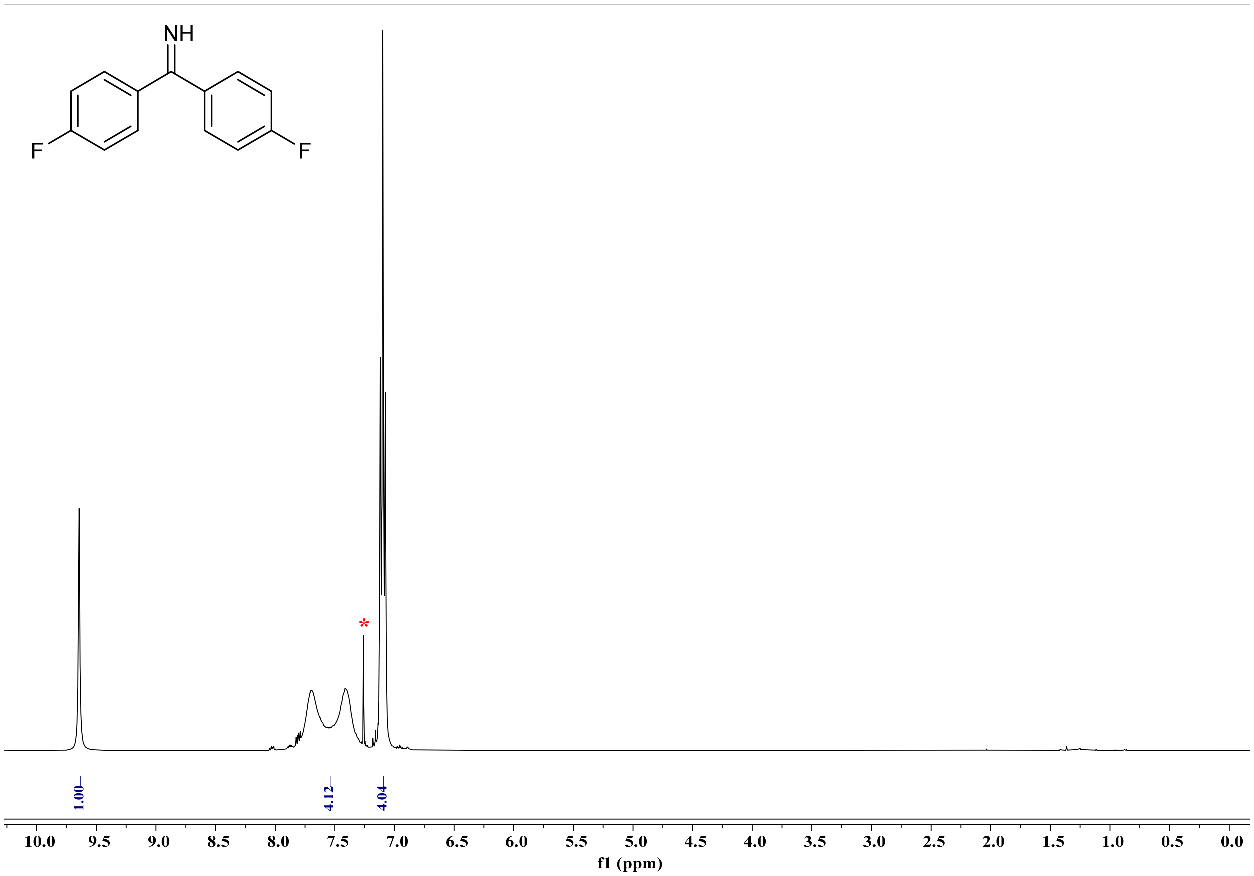

Figure S35. ^{1}H NMR spectrum (400 MHz, $CDCl_3$) of bis(4-fluorophenyl)methanimine.

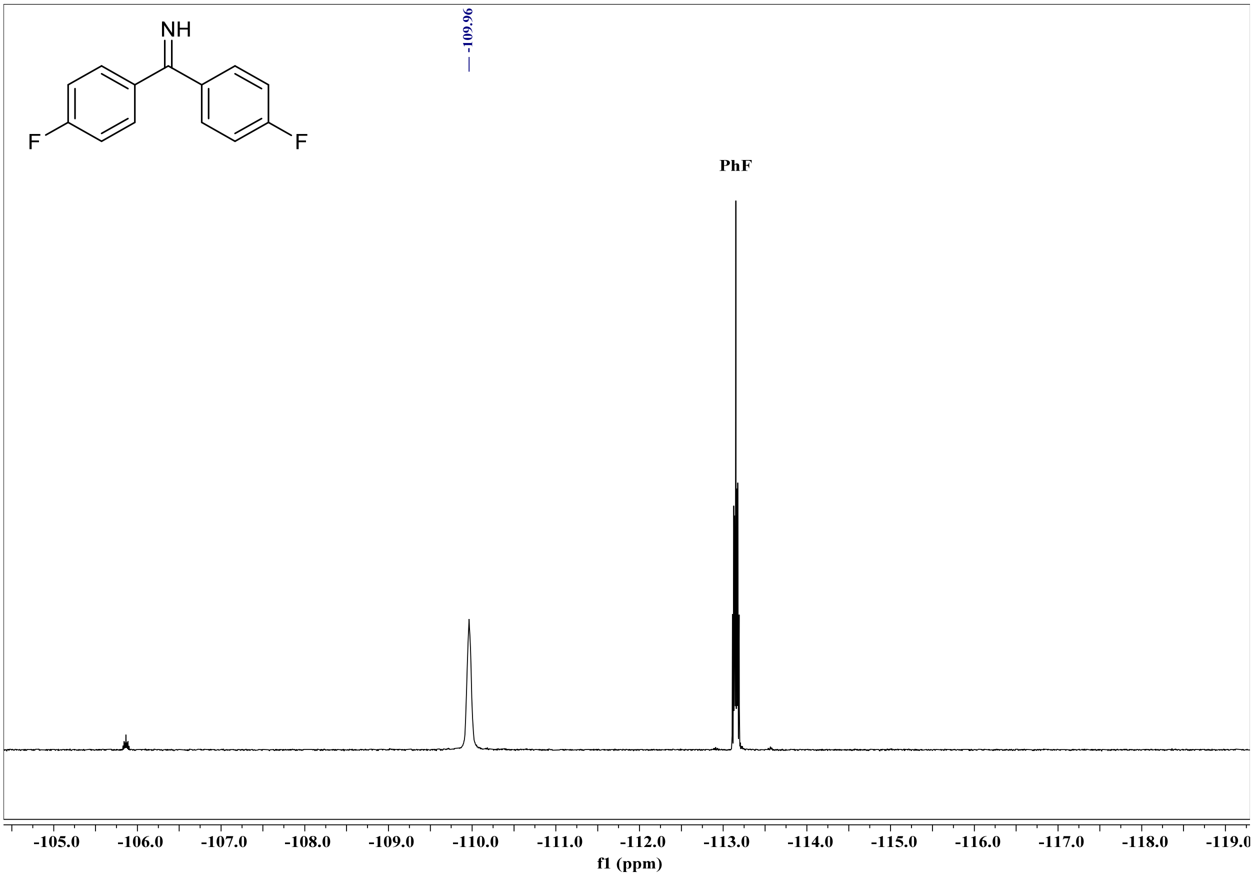

Figure S36. ^{19}F NMR spectrum (376 MHz, $CDCl_3$) of bis(4-fluorophenyl)methanimine.

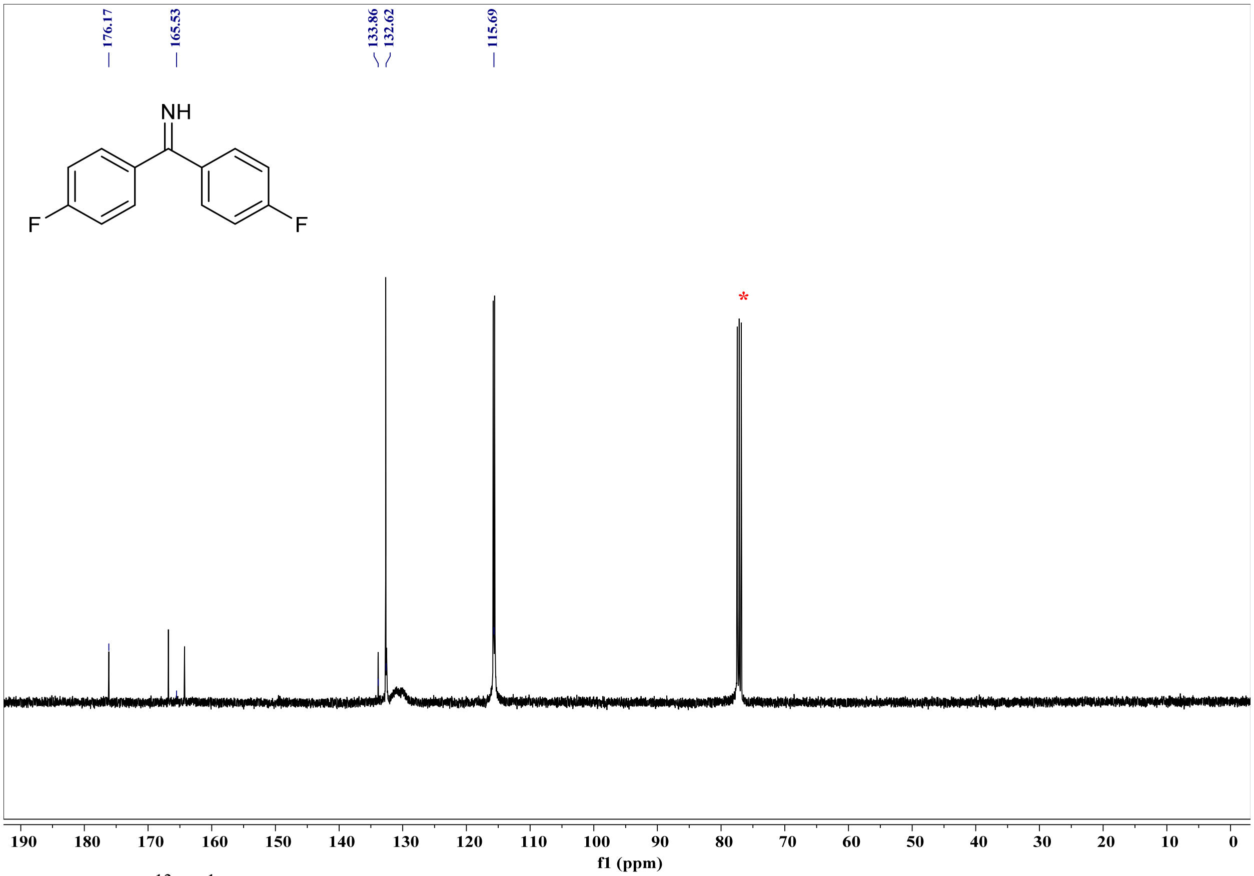

Figure S37. $^{13}C\{^{1}H\}$ NMR spectrum (100.47 MHz, $CDCl_3$) of bis(4-fluorophenyl)methanimine.

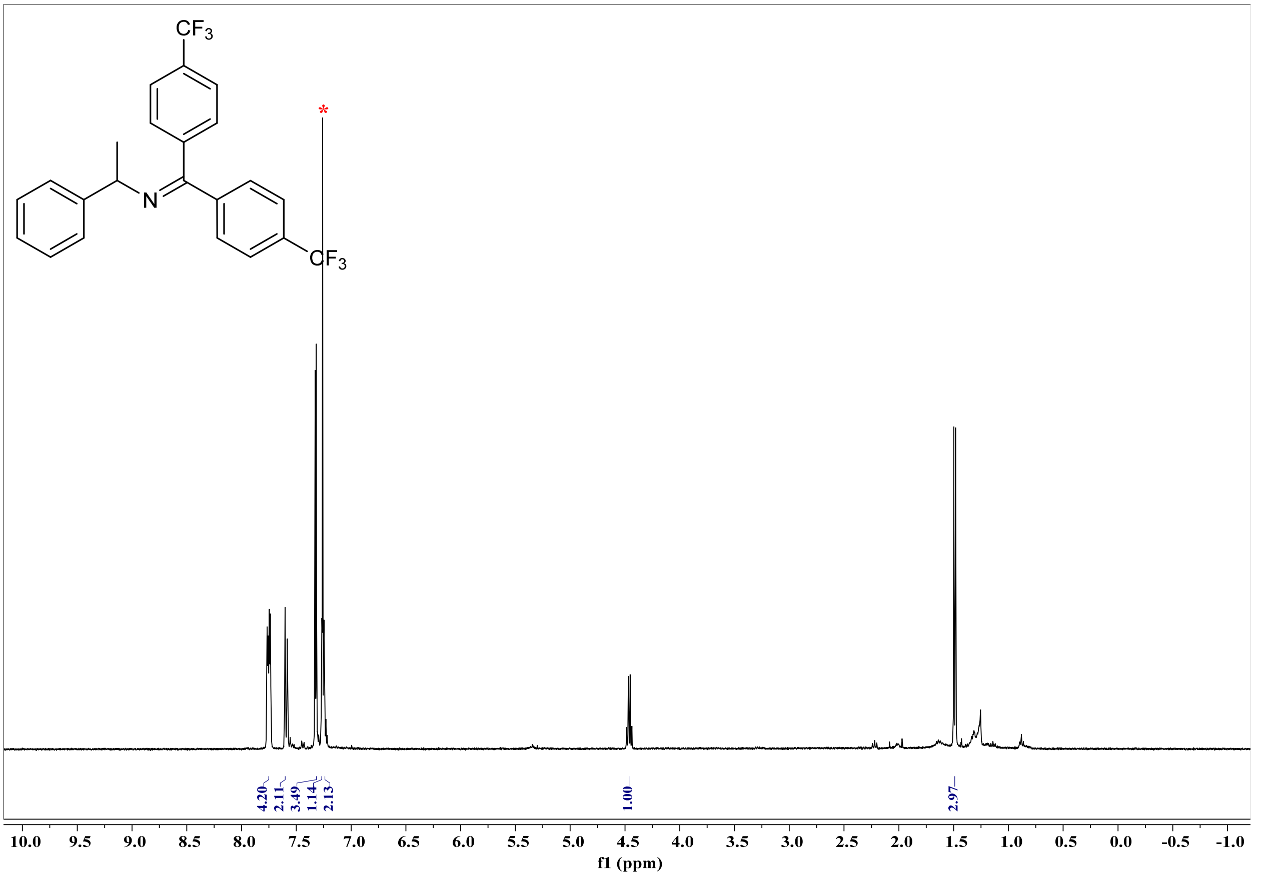

Figure S38. ^{1}H NMR spectrum (400 MHz, $CDCl_3$) of *N*-(1-phenylethyl)-1,1-bis(4-(trifluoromethyl)phenyl)methanimine (**5a-CF$_3$**).

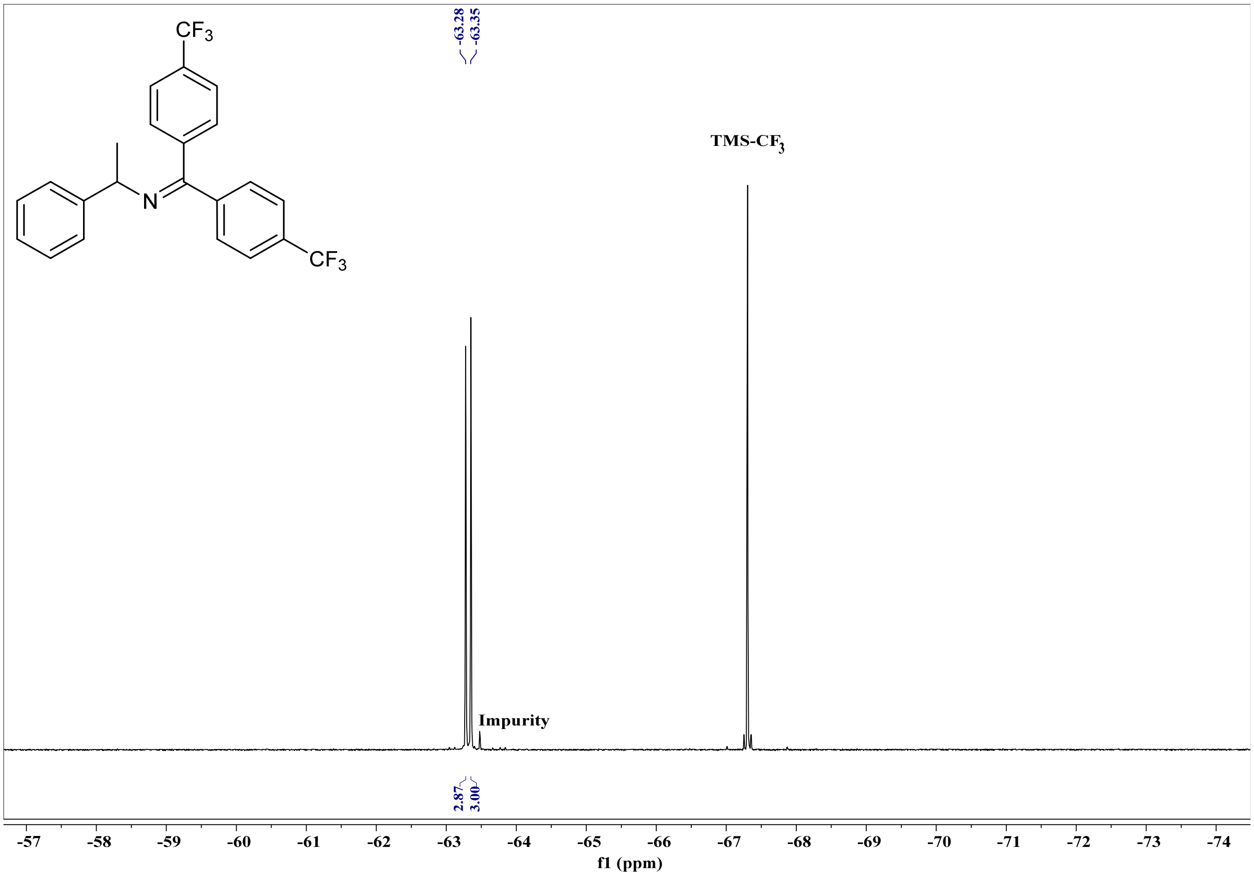

Figure S39. ^{19}F NMR spectrum (376 MHz, $CDCl_3$) of *N*-(1-phenylethyl)-1,1-bis(4-(trifluoromethyl)phenyl)methanimine (**5a-CF$_3$**).

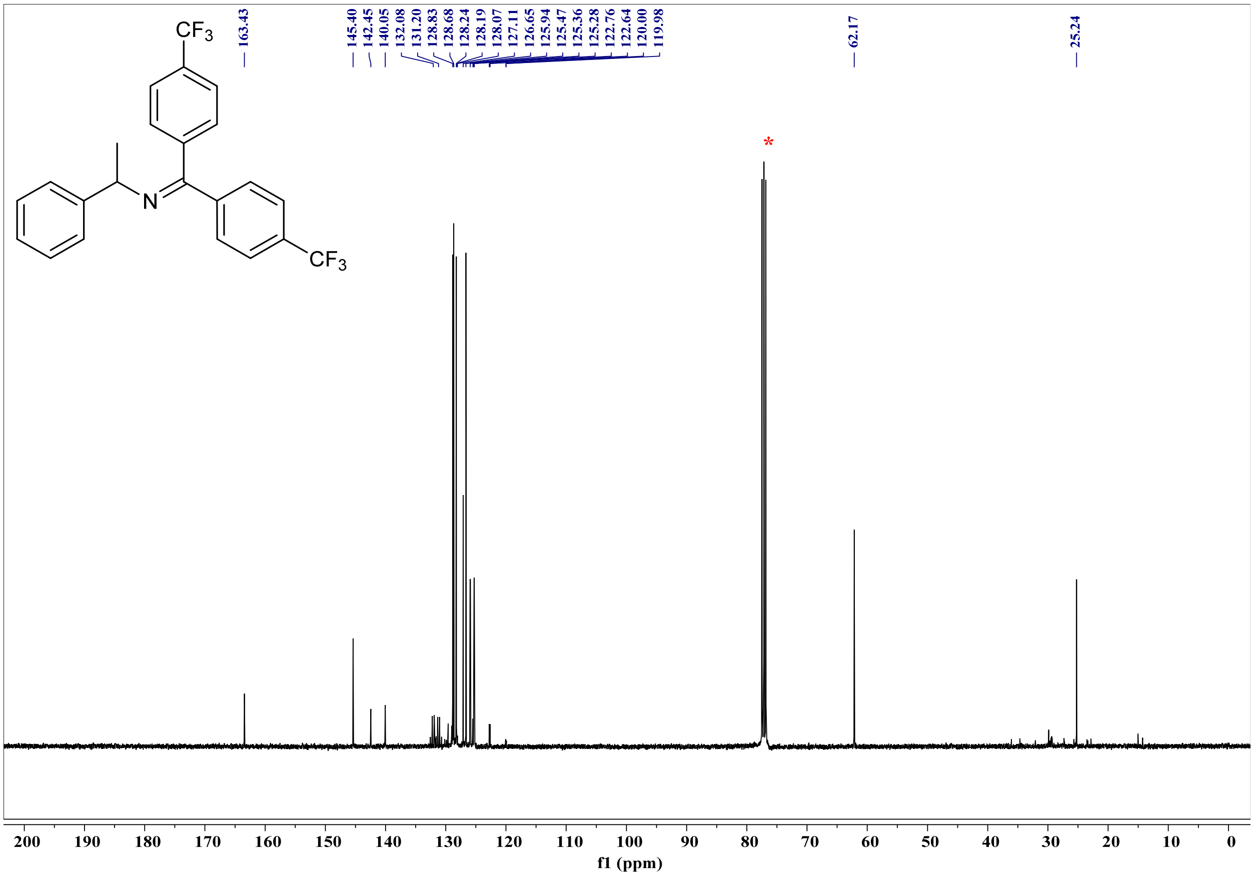

Figure S40. $^{13}C\{^1H\}$ NMR spectrum (100.47 MHz, $CDCl_3$) of *N*-(1-phenylethyl)-1,1-bis(4-(trifluoromethyl)phenyl)methanimine (**5a-CF$_3$**).

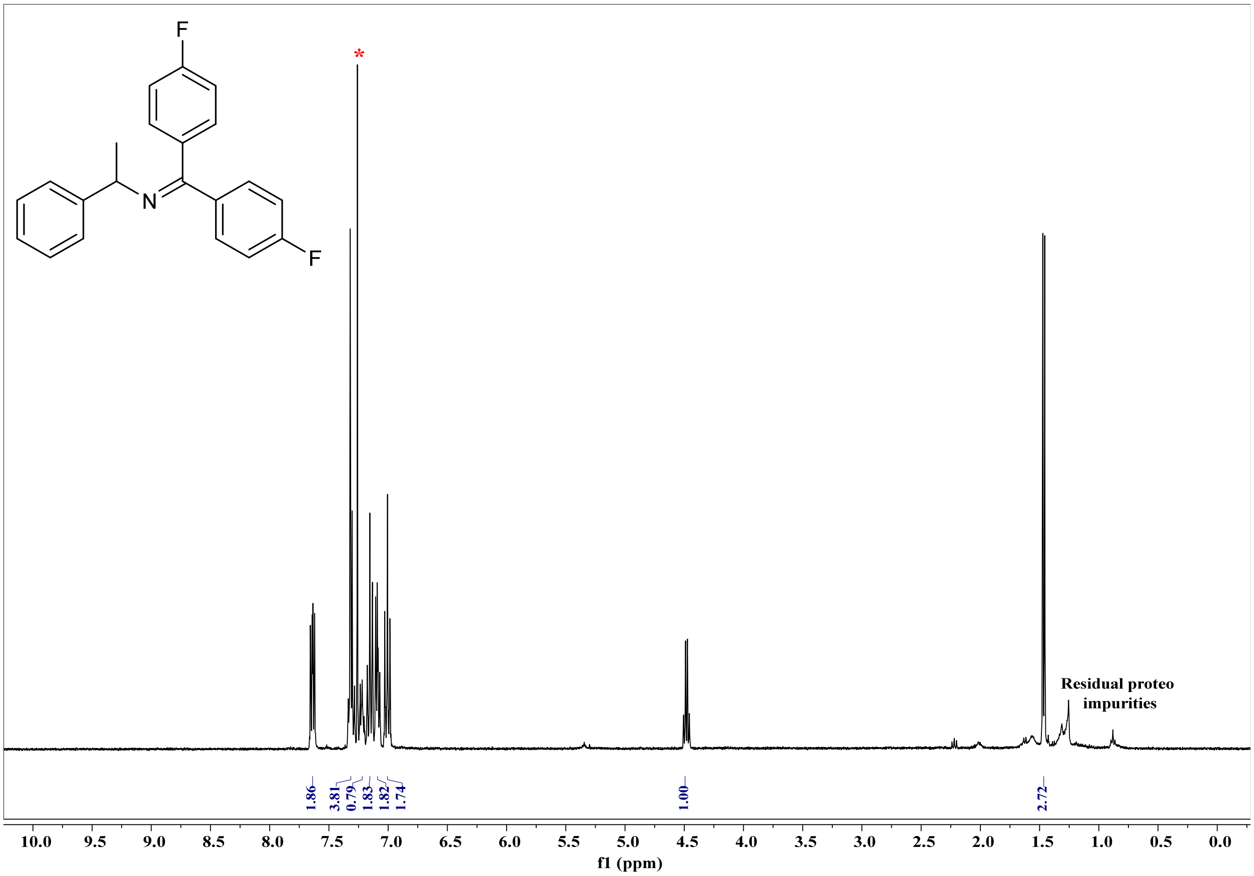

Figure S41. ^{1}H NMR spectrum (400 MHz, $CDCl_3$) of *N*-(1-phenylethyl)-1,1-bis(4-fluorophenyl)-methanimine (**5a-F**).

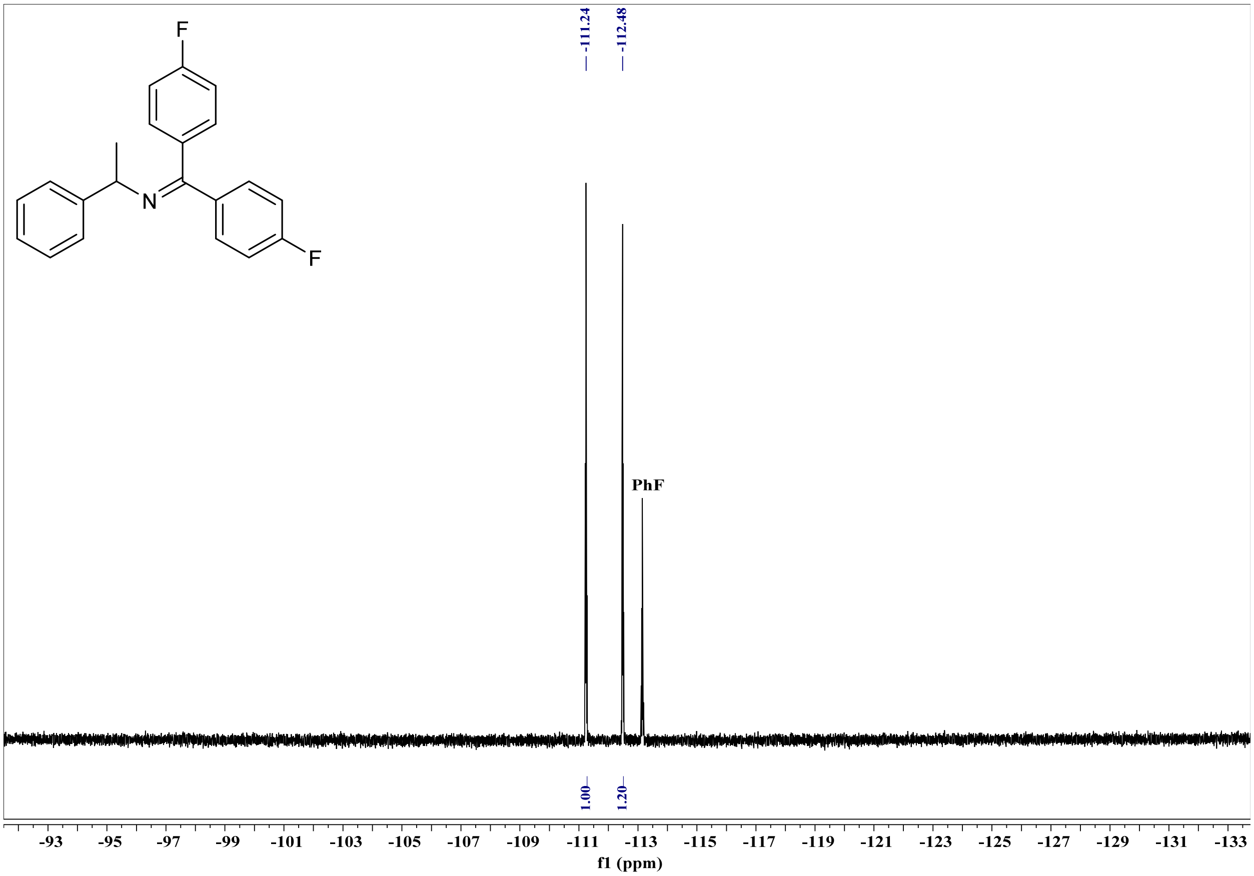

Figure S42. ^{19}F NMR spectrum (376 MHz, $CDCl_3$) of *N*-(1-phenylethyl)-1,1-bis(4-fluorophenyl)-methanimine (**5a-F**).

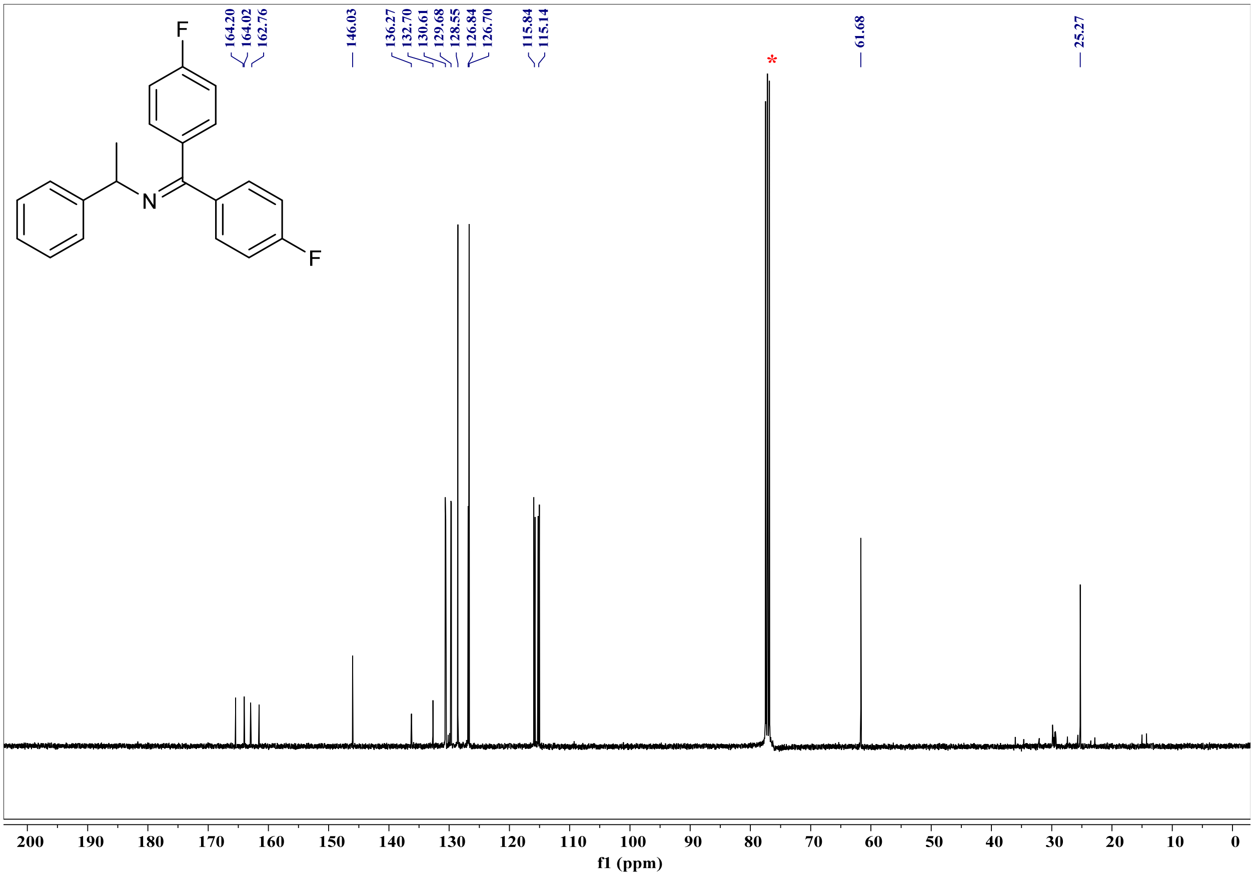

Figure S43. $^{13}C\{^1H\}$ NMR spectrum (100.47 MHz, $CDCl_3$) of *N*-(1-phenylethyl)-1,1-bis(4-fluorophenyl)-methanimine (**5a-F**).

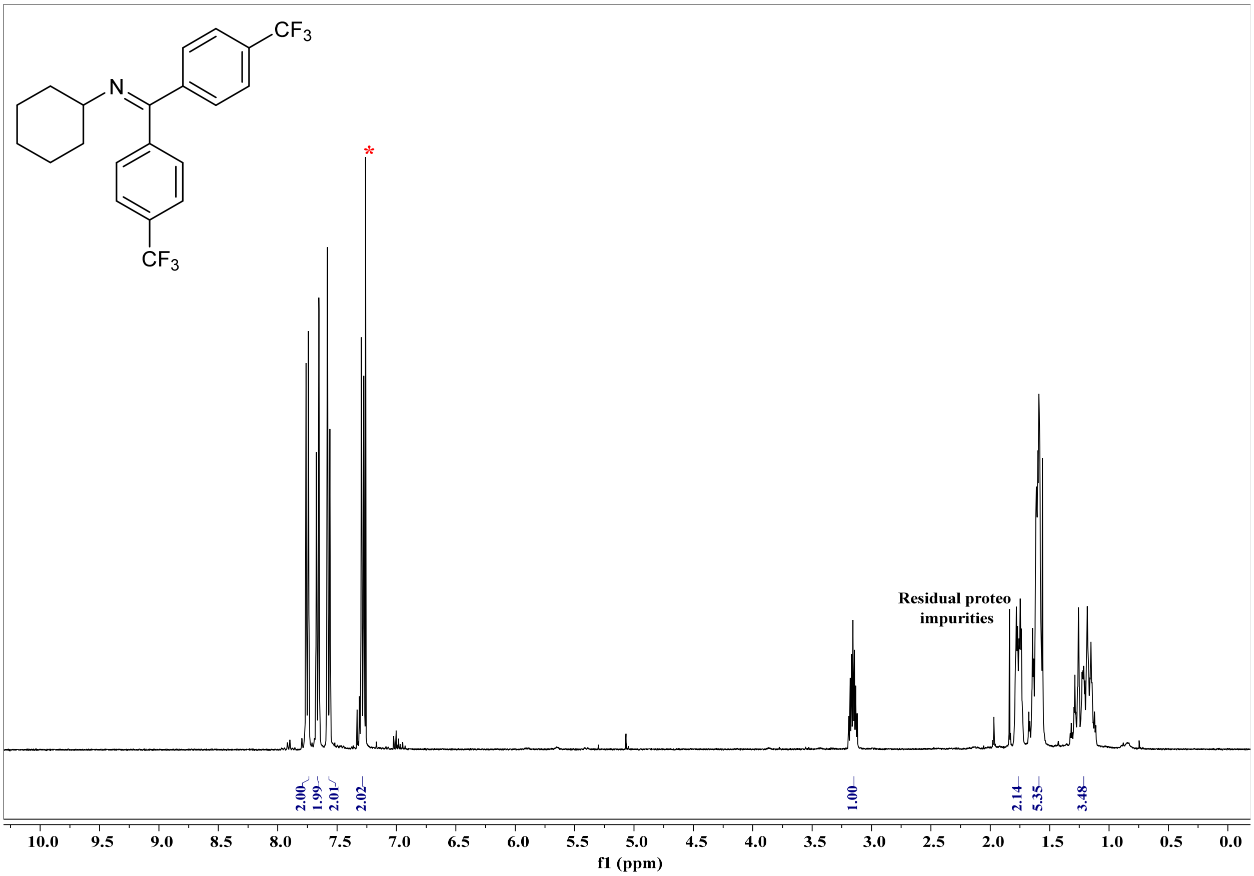

Figure S44. ^{1}H NMR spectrum (400 MHz, $CDCl_3$) of *N*-cyclohexyl-1,1-bis(4-(trifluoromethyl)phenyl)methanimine (**5b-CF$_3$**).

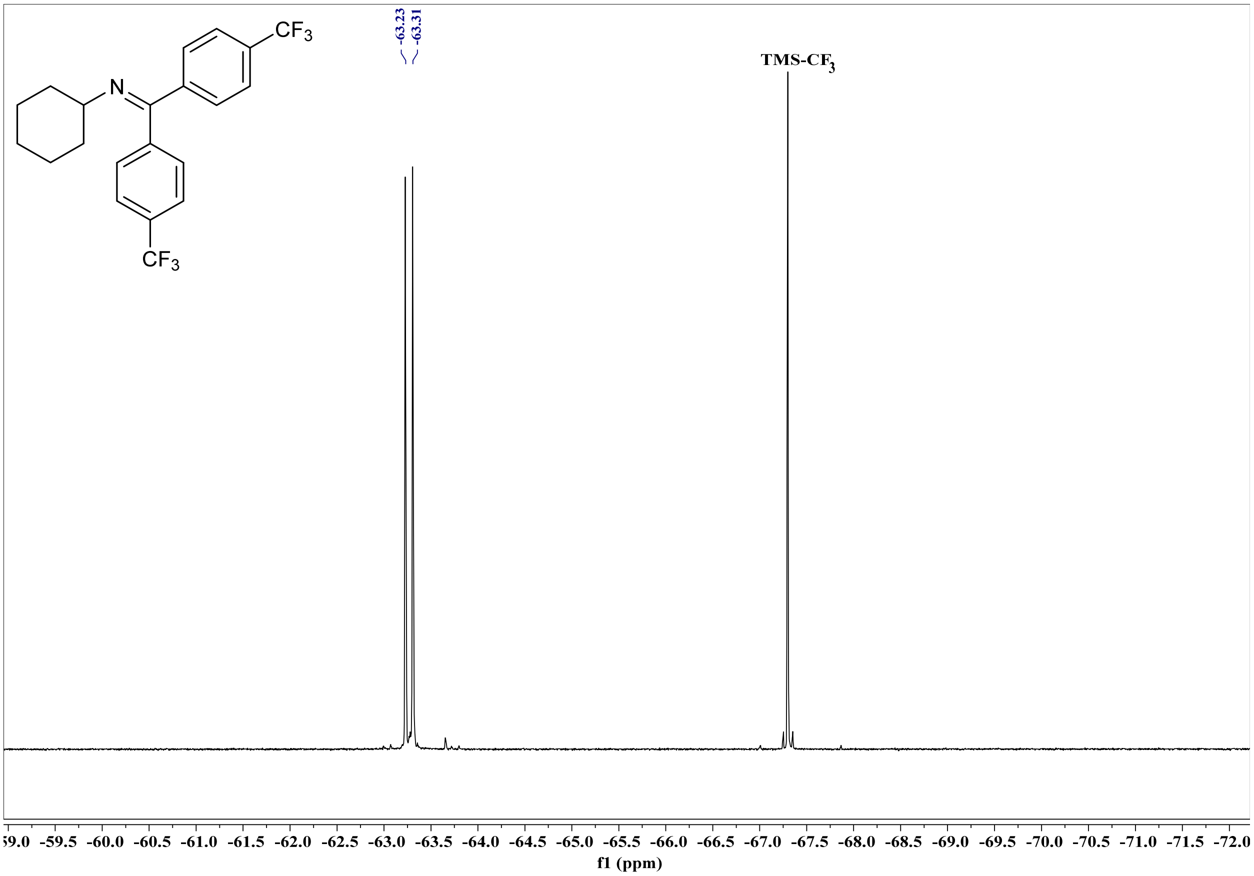

Figure S45. ^{19}F NMR spectrum (376 MHz, $CDCl_3$) of *N*-cyclohexyl-1,1-bis(4-(trifluoromethyl)phenyl)methanimine (**5b-CF_3**).

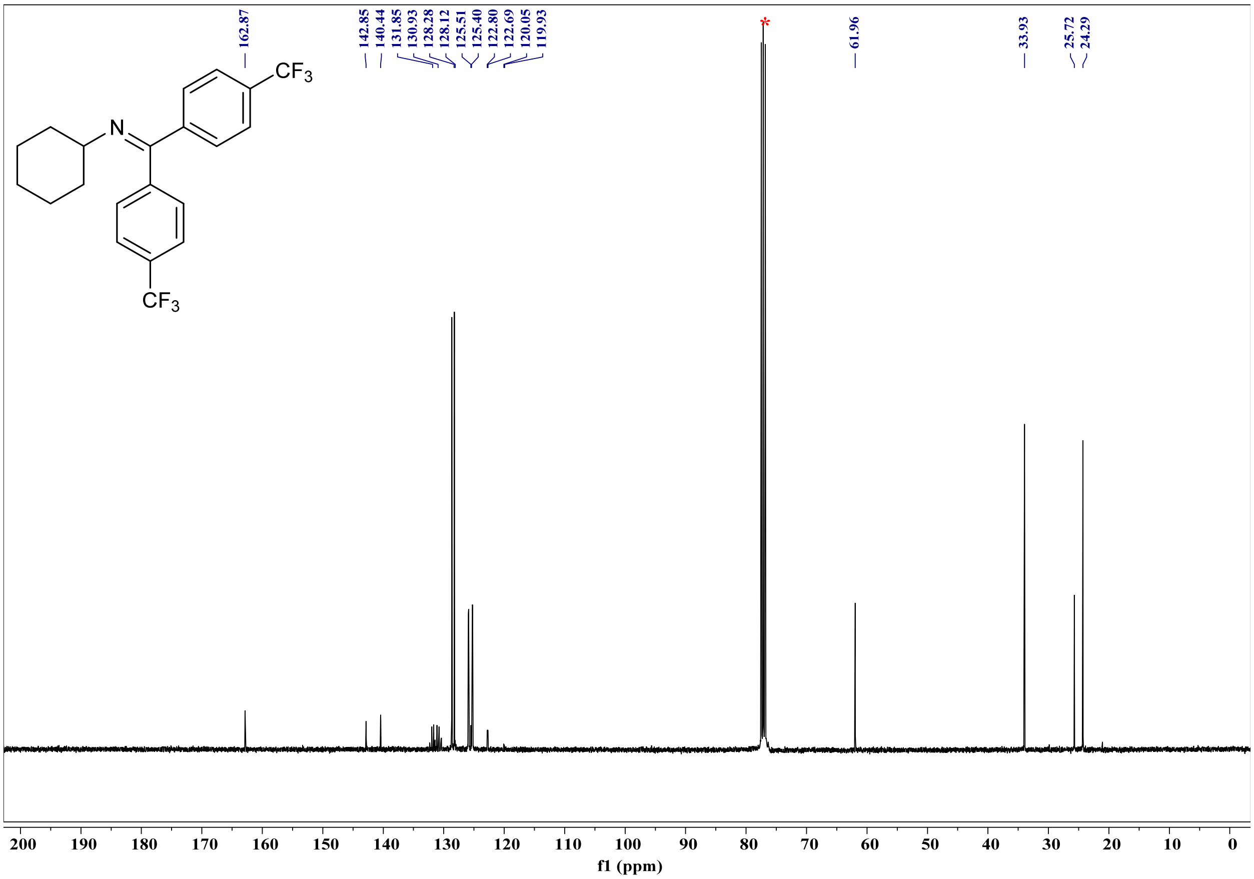

Figure S46. $^{13}C\{^{1}H\}$ NMR spectrum (100.47 MHz, $CDCl_3$) of *N*-cyclohexyl-1,1-bis(4-(trifluoromethyl)phenyl)methanimine (**5b-CF$_3$**).

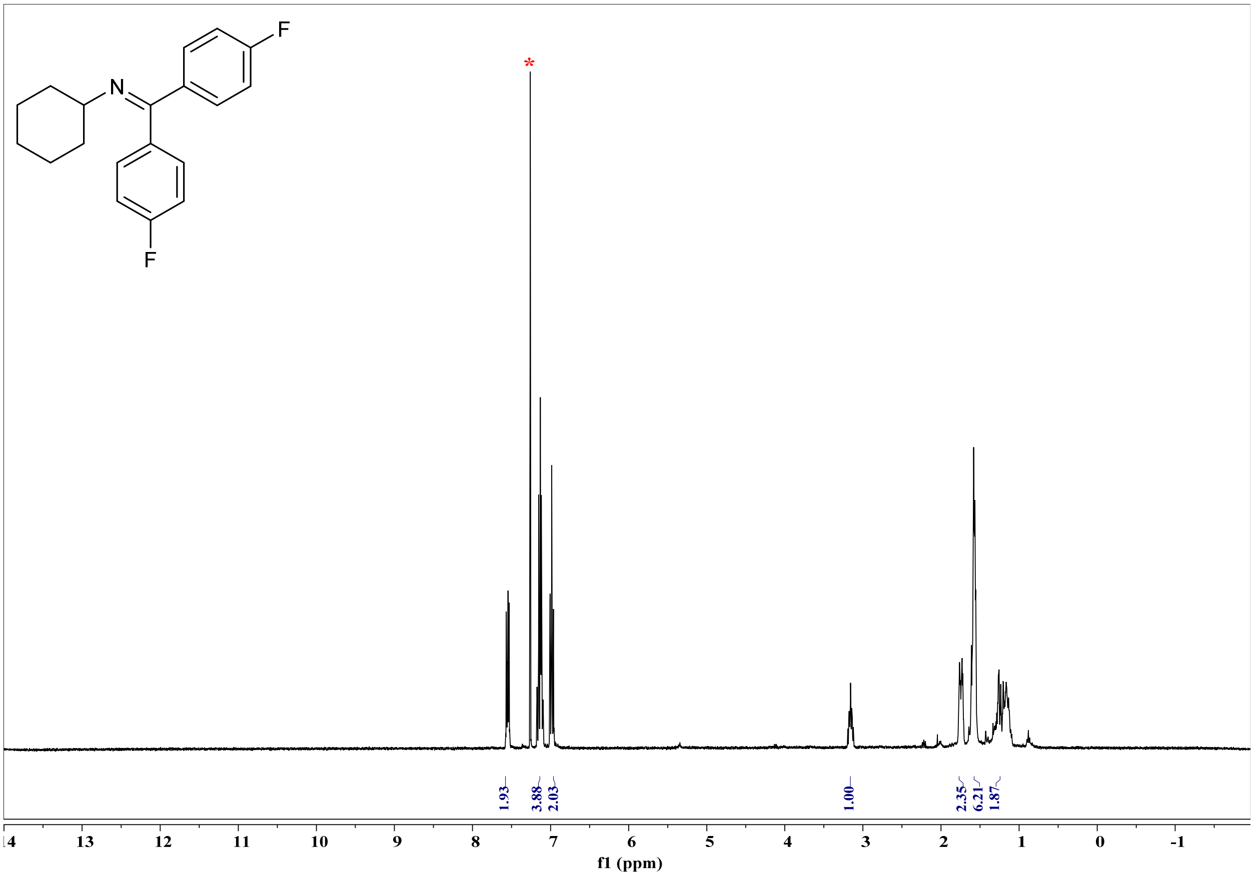

Figure S47. ^{1}H NMR spectrum (400 MHz, $CDCl_3$) of *N*-cyclohexyl-1,1-bis(4-fluorophenyl)methanimine (**5b-F**).

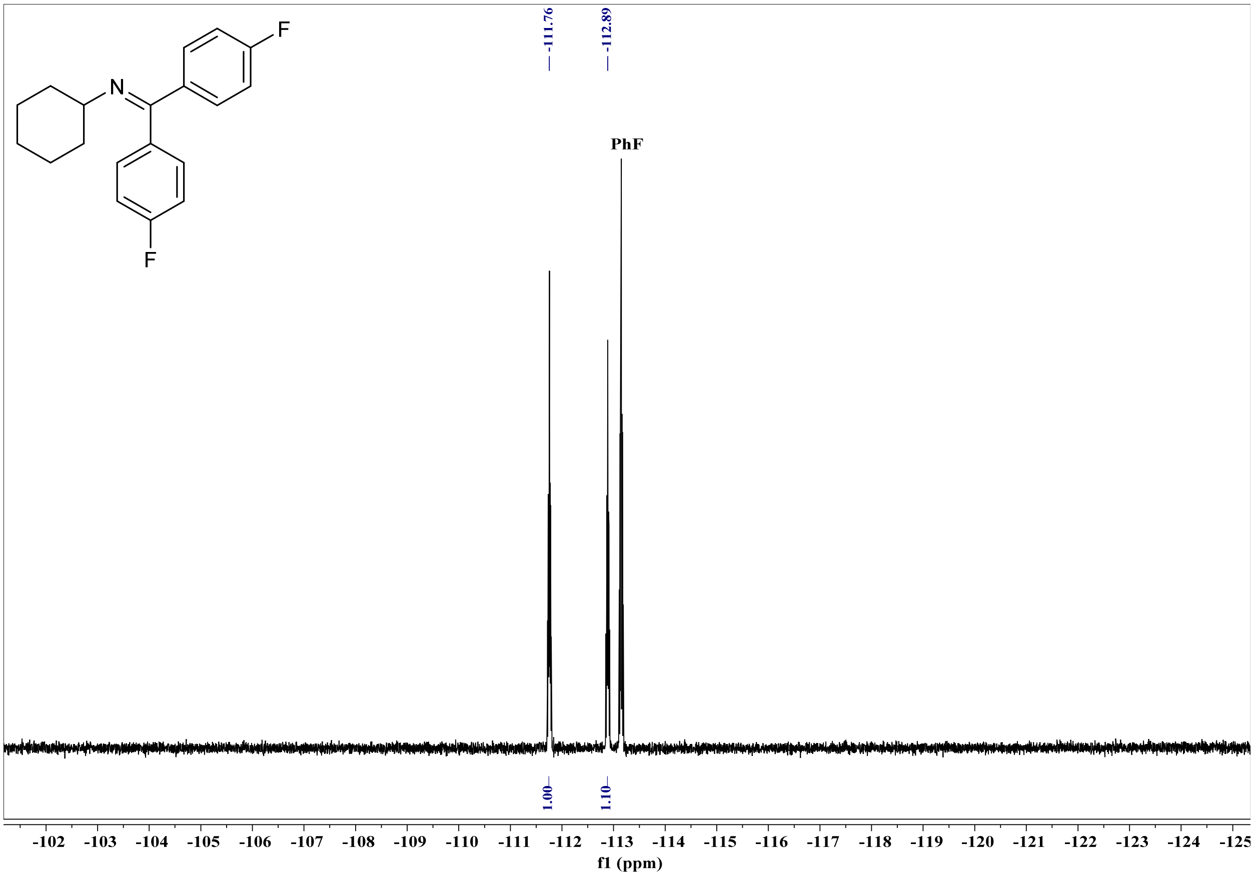

Figure S48. ^{19}F NMR spectrum (376 MHz, $CDCl_3$) of *N*-cyclohexyl-1,1-bis(4-fluorophenyl)methanimine (**5b-F**).

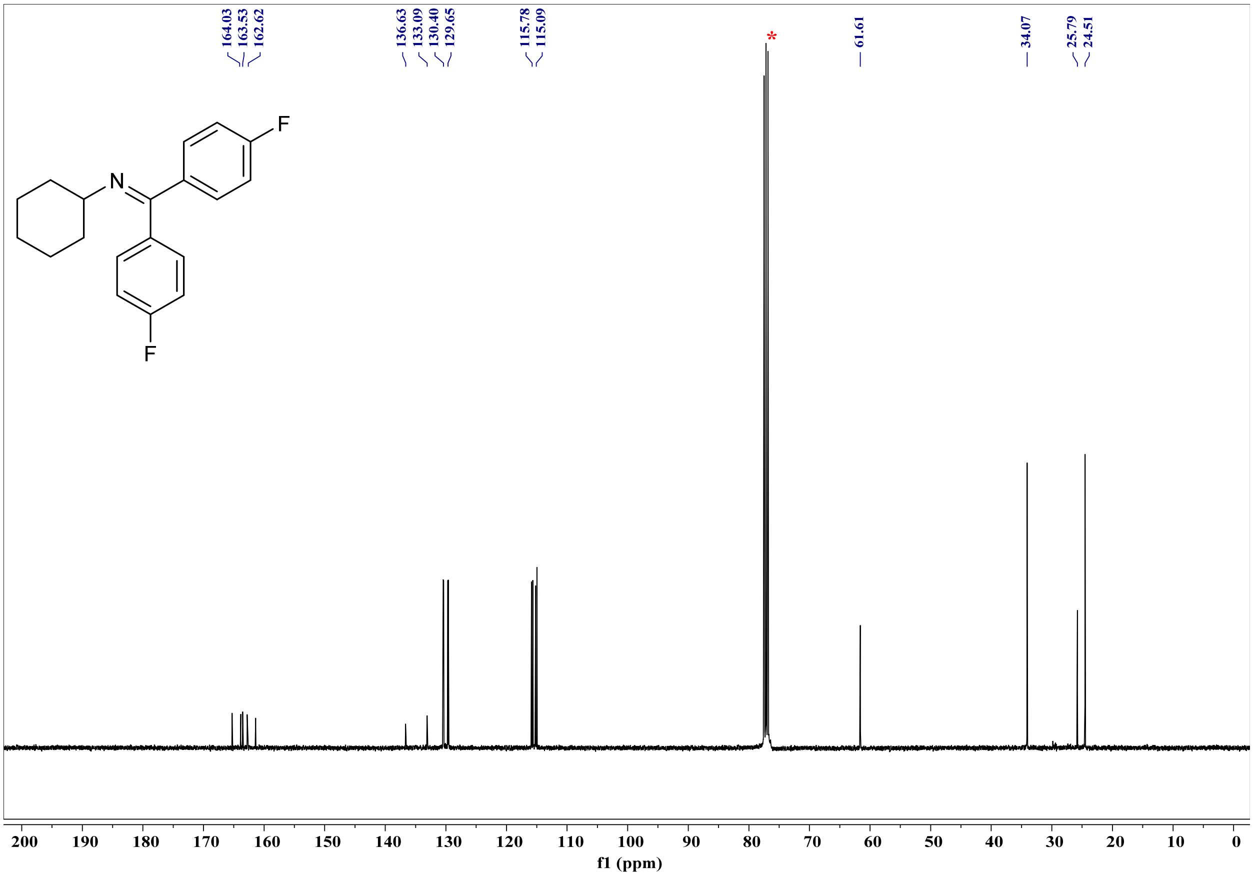

Figure S49. $^{13}C\{^{1}H\}$ NMR spectrum (100.47 MHz, $CDCl_3$) of *N*-cyclohexyl-1,1-bis(4-fluorophenyl)methanimine (**5b-F**).

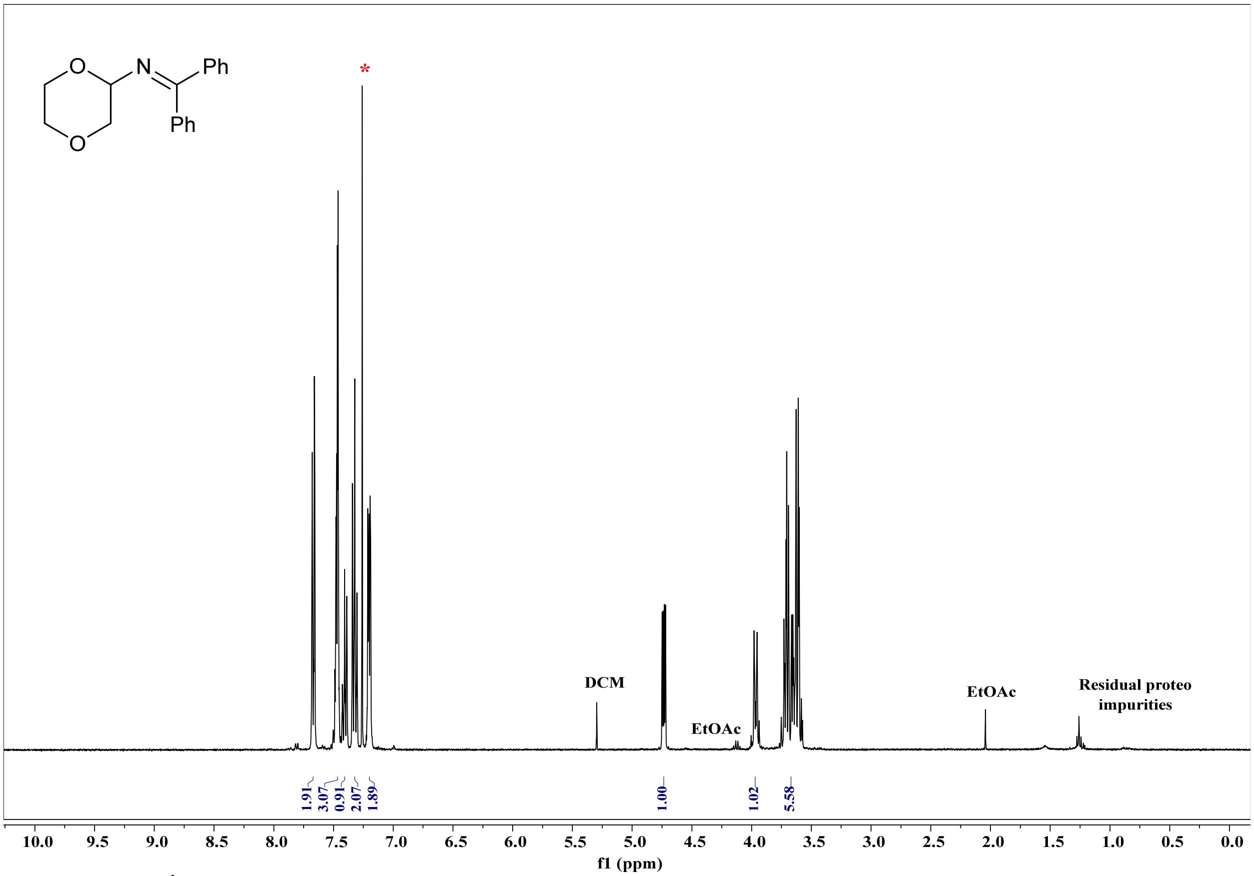

Figure S50. ^{1}H NMR spectrum (400 MHz, $CDCl_3$) of *N*-(1,4-dioxan-2-yl)-1,1-diphenylmethanimine (**6a**).

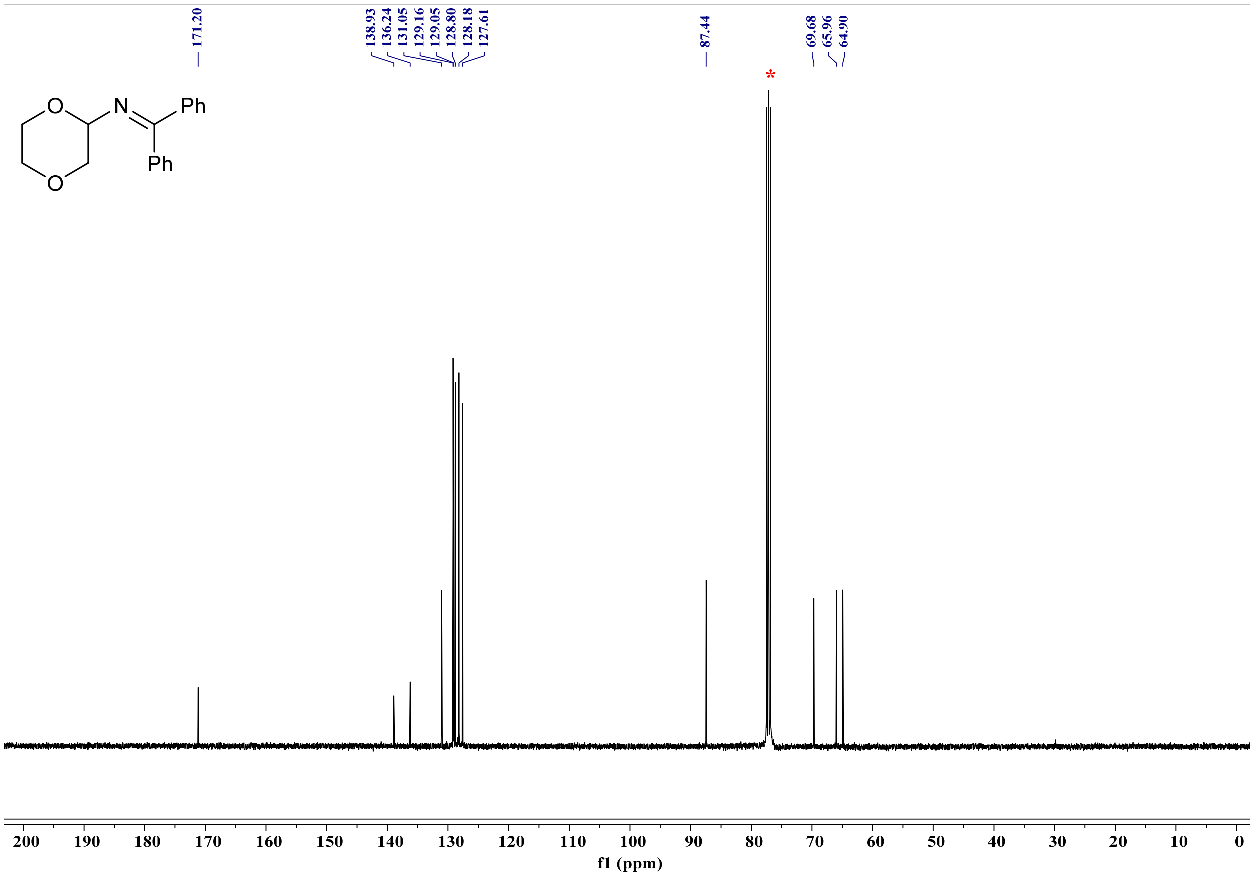

Figure S51. $^{13}C\{^{1}H\}$ NMR spectrum (100.47 MHz, $CDCl_3$) of *N*-(1,4-dioxan-2-yl)-1,1-diphenylmethanimine (**6a**).

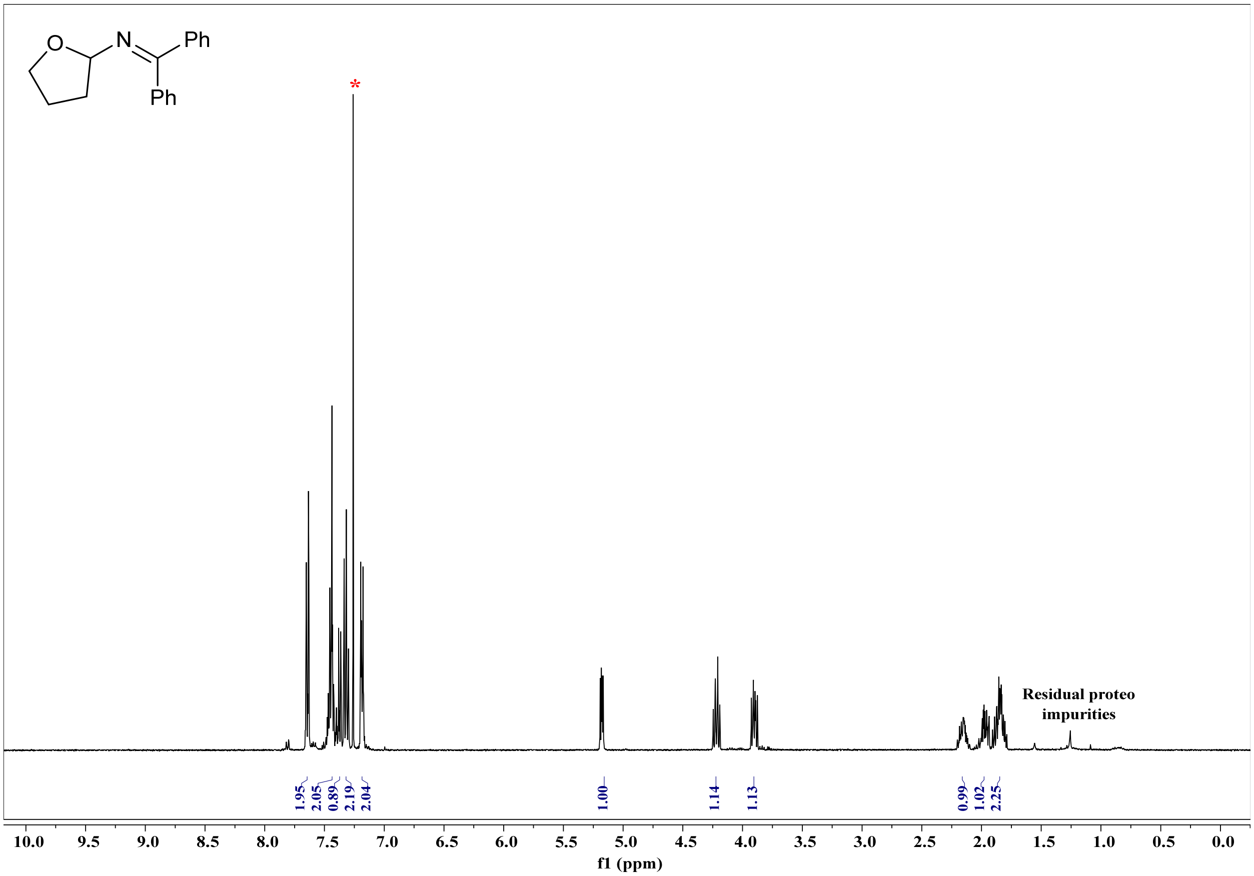

Figure S52. ^{1}H NMR spectrum (400 MHz, $CDCl_3$) of *N*-(tetrahydrofuran-2-yl)-1,1-diphenylmethanimine (**6b**).

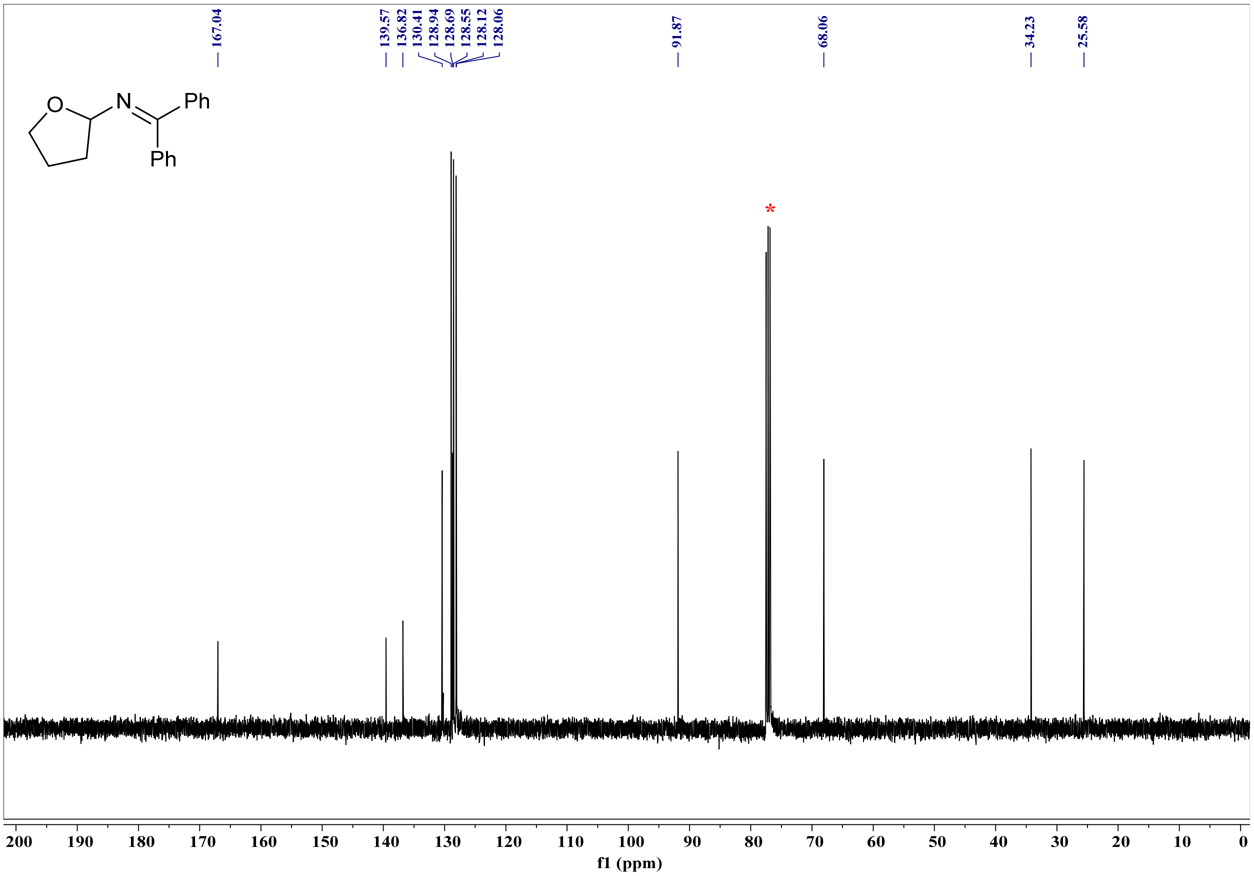

Figure S53. $^{13}C\{^{1}H\}$ NMR spectrum (100.47 MHz, $CDCl_3$) of *N*-(tetrahydrofuran-2-yl)-1,1-diphenylmethanimine (**6b**).

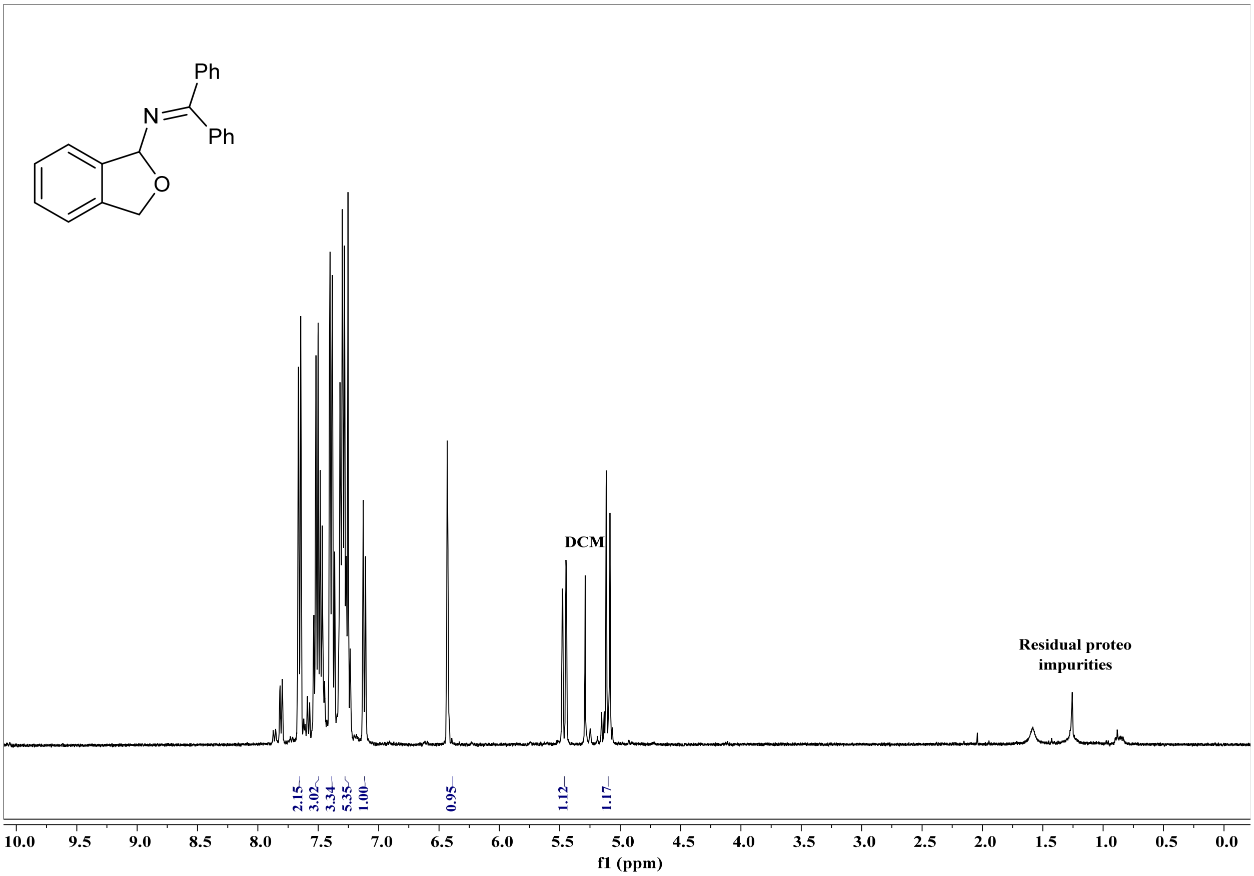

Figure S54. ^{1}H NMR spectrum (400 MHz, $CDCl_3$) of *N*-(1,3-dihydroisobenzofuran-1-yl)-1,1-diphenylmethanimine (**6c**).

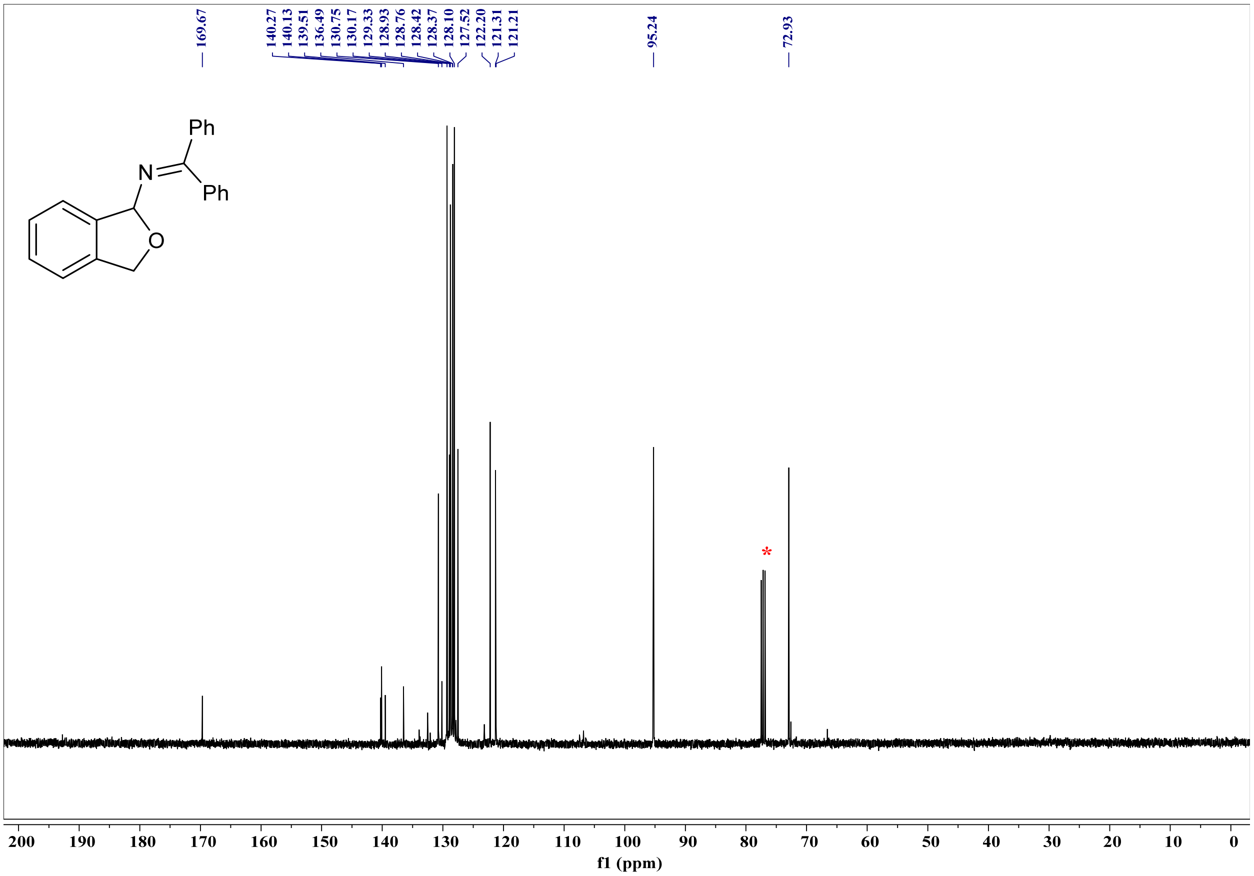

Figure S55. $^{13}C\{^1H\}$ NMR spectrum (100.47 MHz, $CDCl_3$) of *N*-(1,3-dihydroisobenzofuran-1-yl)-1,1-diphenylmethanimine (**6c**).

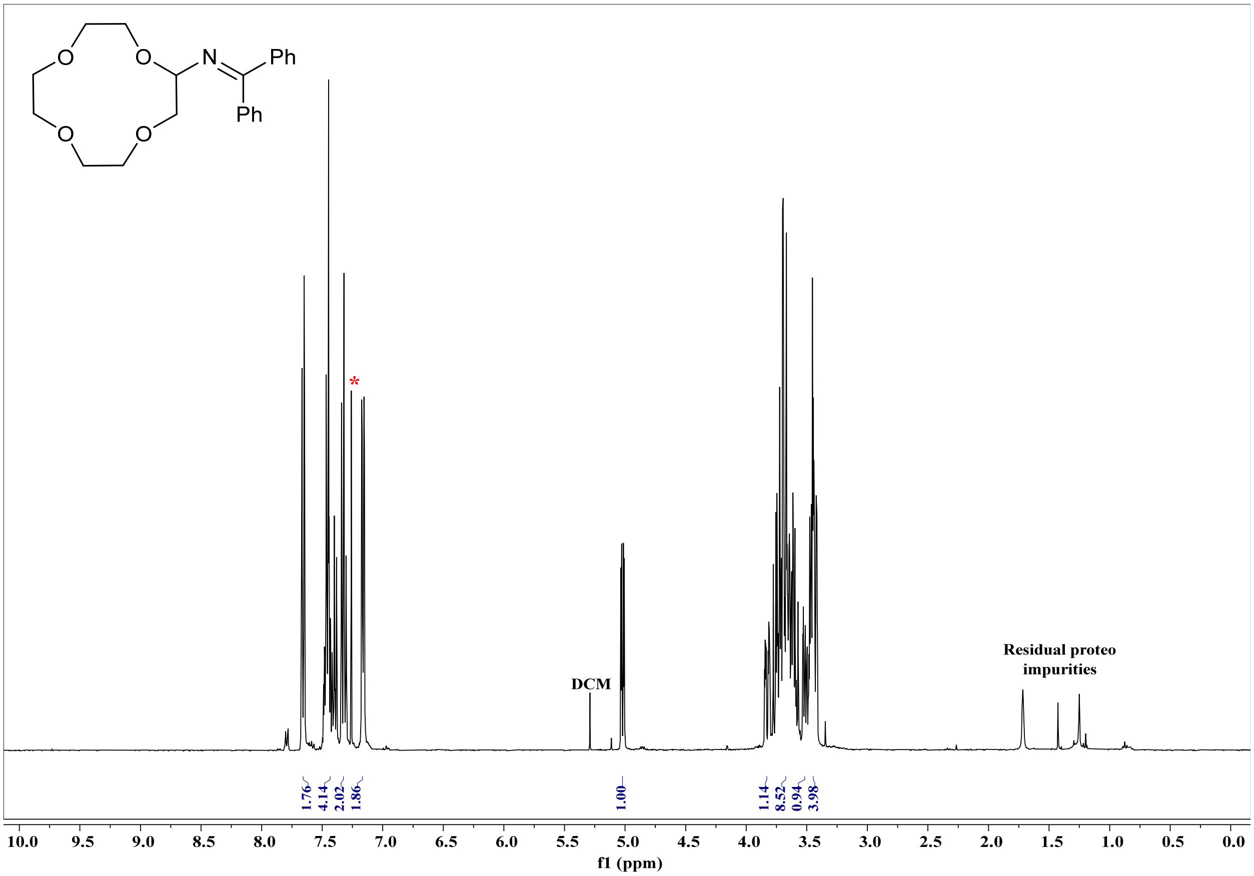

Figure S56. ^{1}H NMR spectrum (400 MHz, $CDCl_3$) of *N*-(1,4,7,10-tetraoxacyclododecan-2-yl)-1,1-diphenylmethanimine (**6d**).

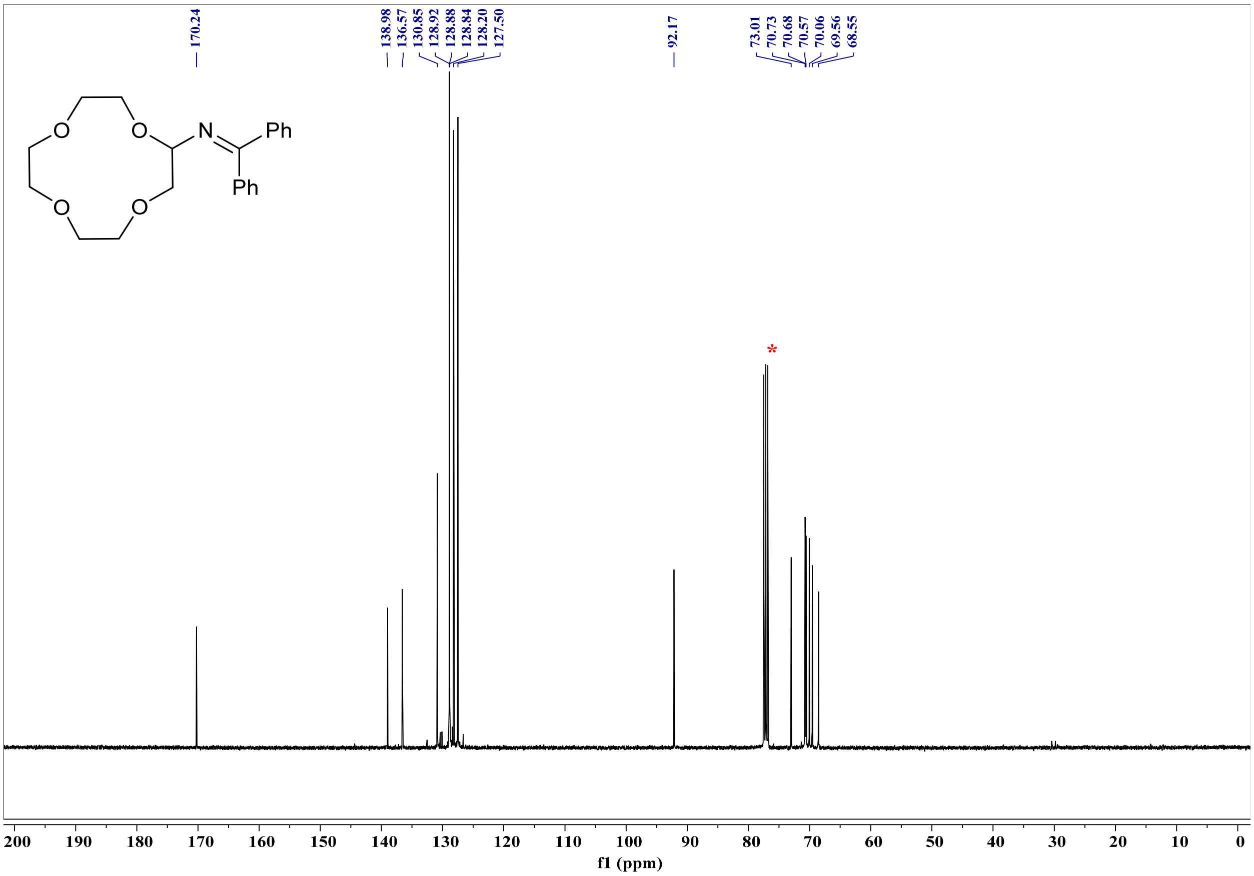

Figure S57. $^{13}C\{^1H\}$ NMR spectrum (100.47 MHz, $CDCl_3$) of *N*-(1,4,7,10-tetraoxacyclododecan-2-yl)-1,1-diphenylmethanimine (**6d**).

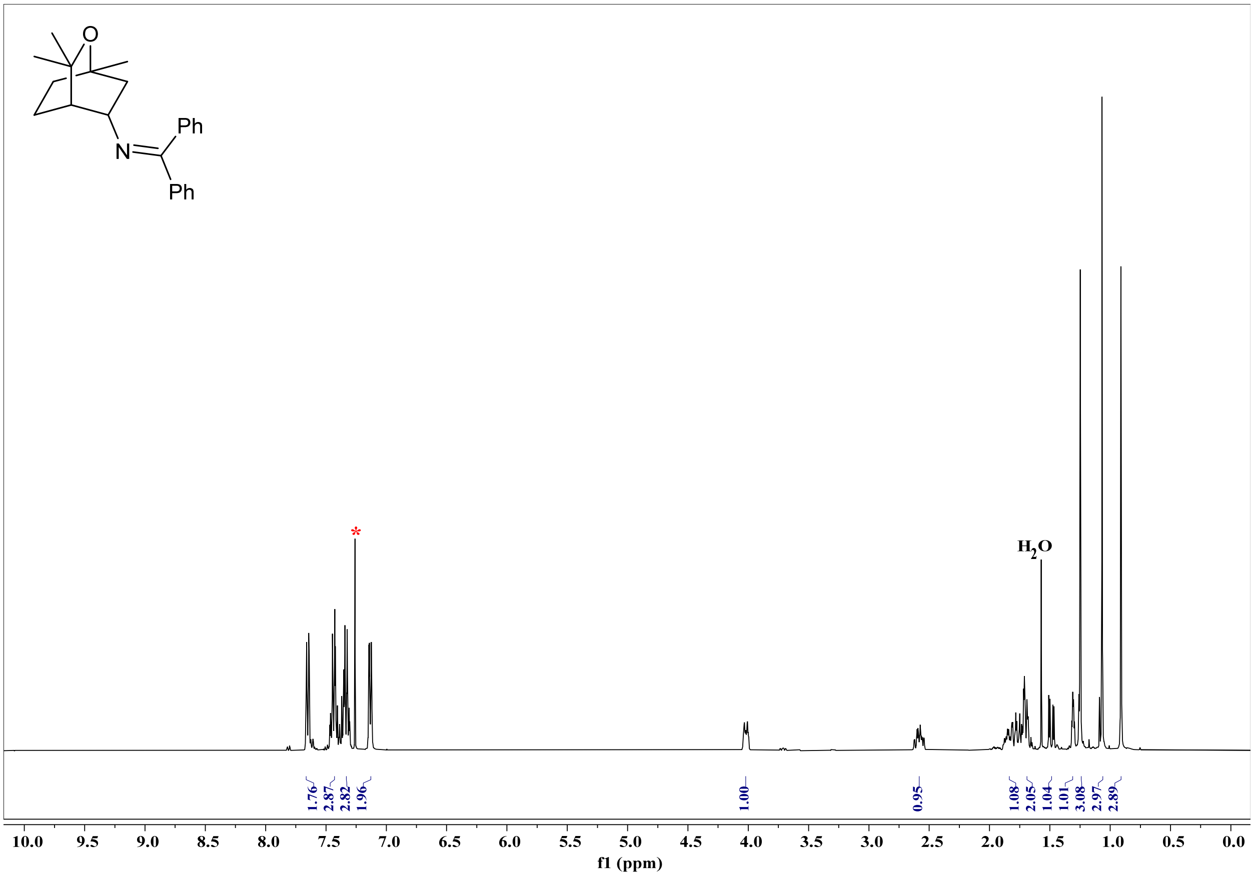

Figure S58. ^{1}H NMR spectrum (400 MHz, $CDCl_3$) of *N*-(1,3,3-trimethyl-2-oxabicyclo[2.2.2]octan-5-yl)-1,1-diphenylmethanimine (**6e**).

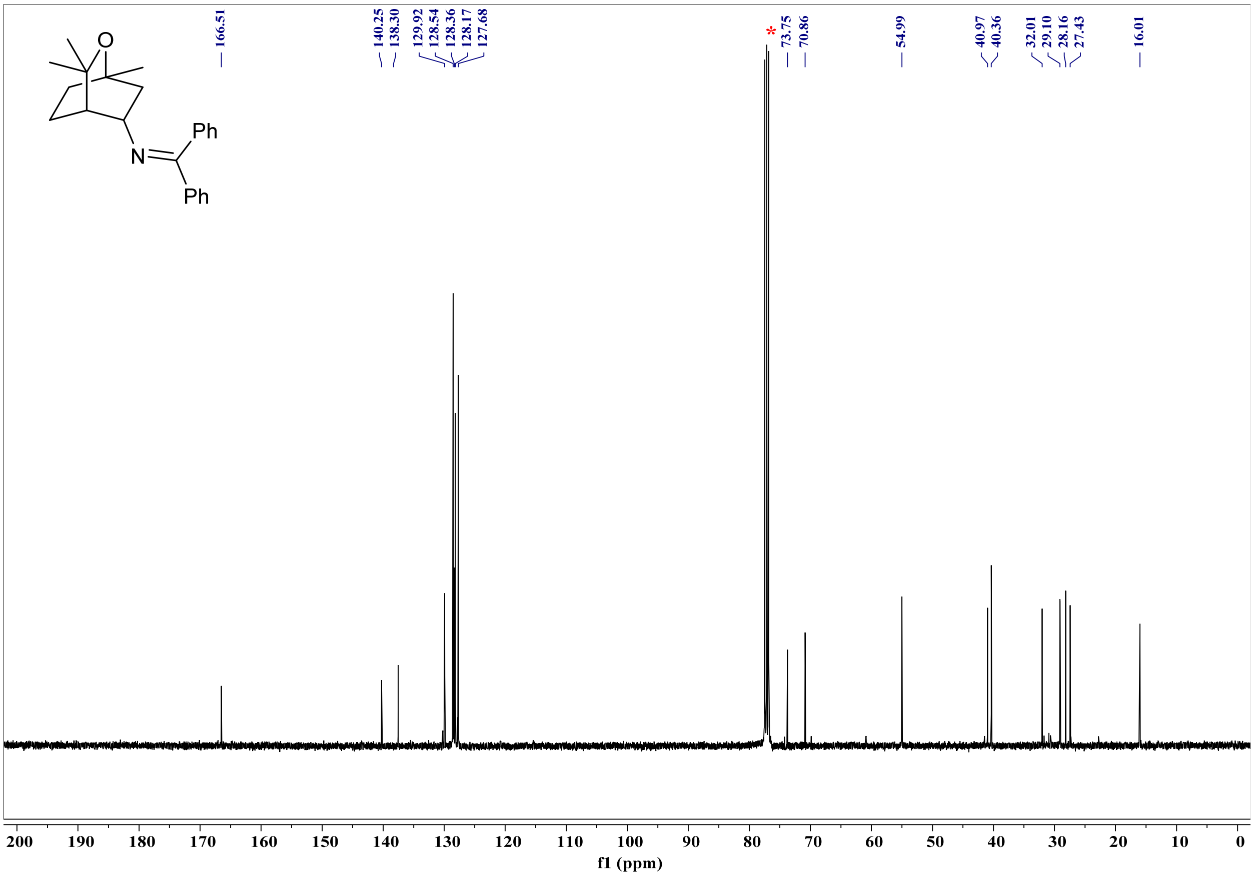

Figure S59. $^{13}C\{^{1}H\}$ NMR spectrum (400 MHz, $CDCl_3$) of *N*-(1,3,3-trimethyl-2-oxabicyclo[2.2.2]octan-5-yl)-1,1-diphenylmethanimine (**6e**).

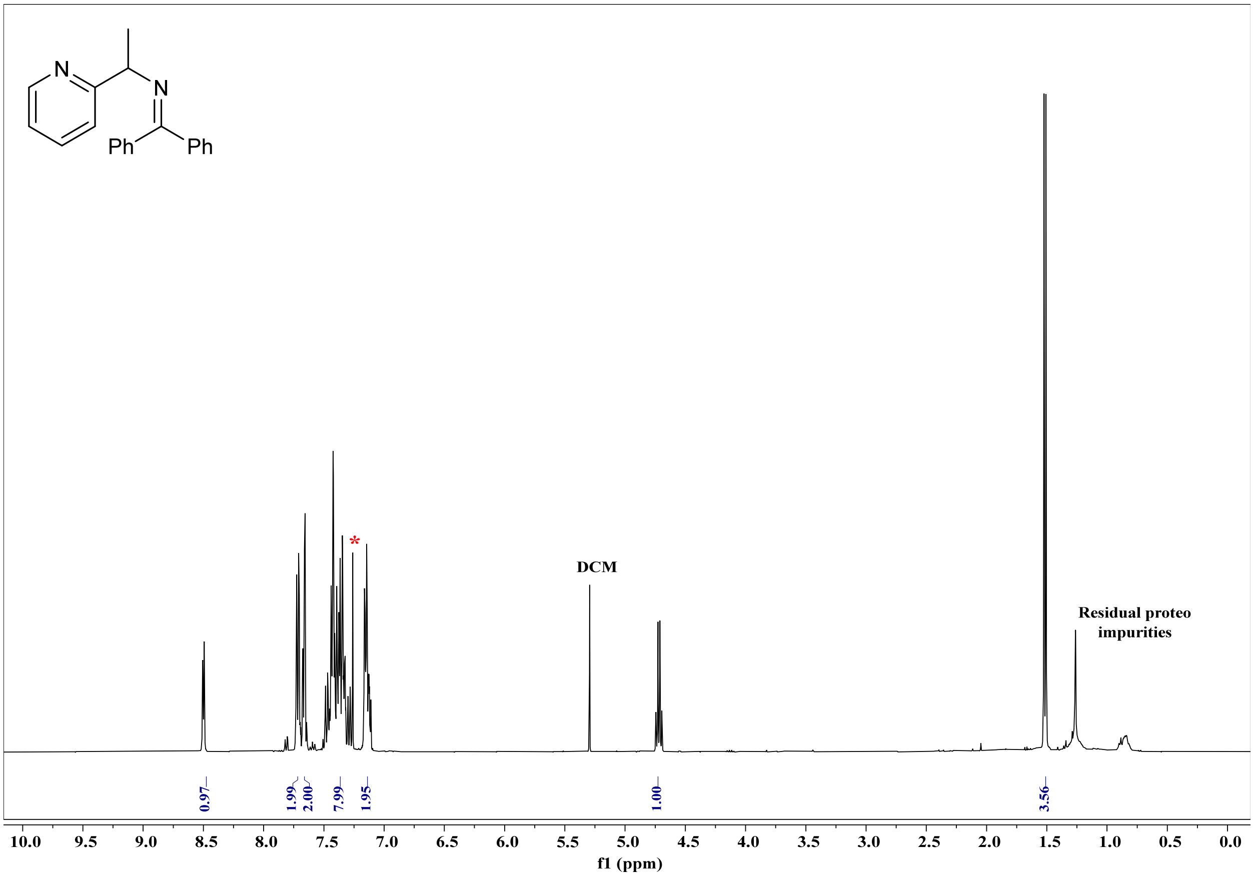

Figure S60. ^{1}H NMR spectrum (400 MHz, $CDCl_3$) of *N*-(1-(pyridin-2-yl)ethyl)-1,1-diphenylmethanimine (**6f**).

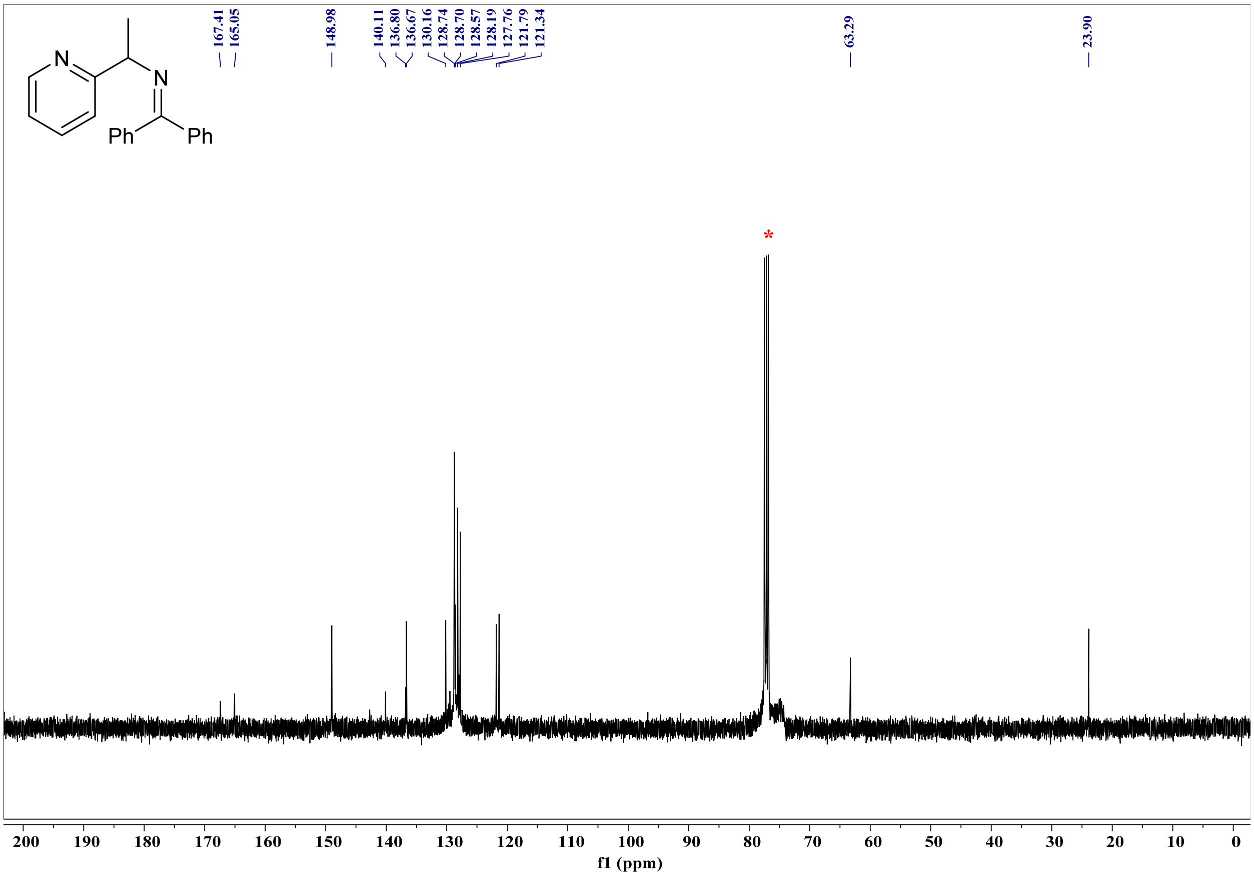

Figure S61. $^{13}C\{^{1}H\}$ NMR spectrum (100.47 MHz, $CDCl_3$) of *N*-(1-(pyridin-2-yl)ethyl)-1,1-diphenylmethanimine (**6f**).

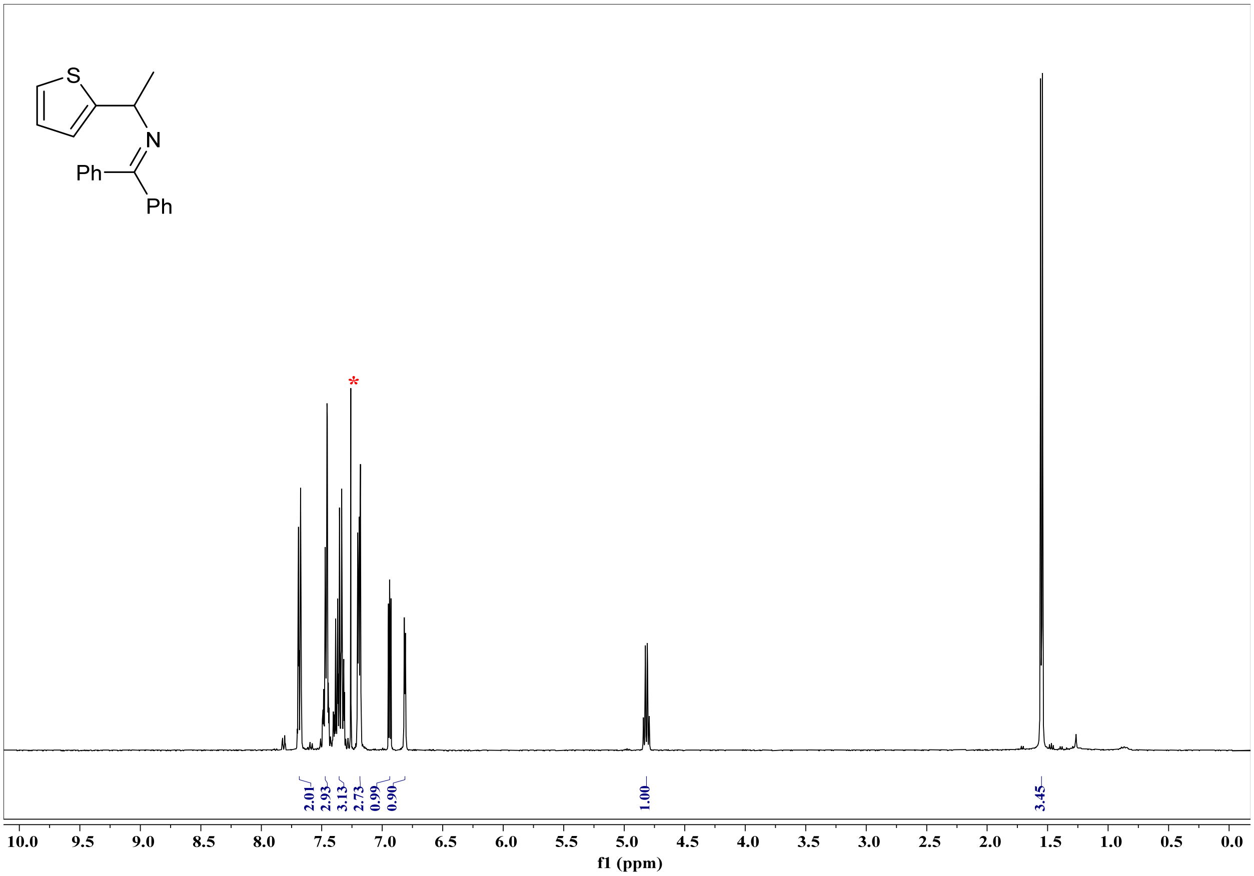

Figure S62. ^{1}H NMR spectrum (400 MHz, $CDCl_3$) of *N*-(1-(thiophen-2-yl)ethyl)-1,1-diphenylmethanimine (**6g**).

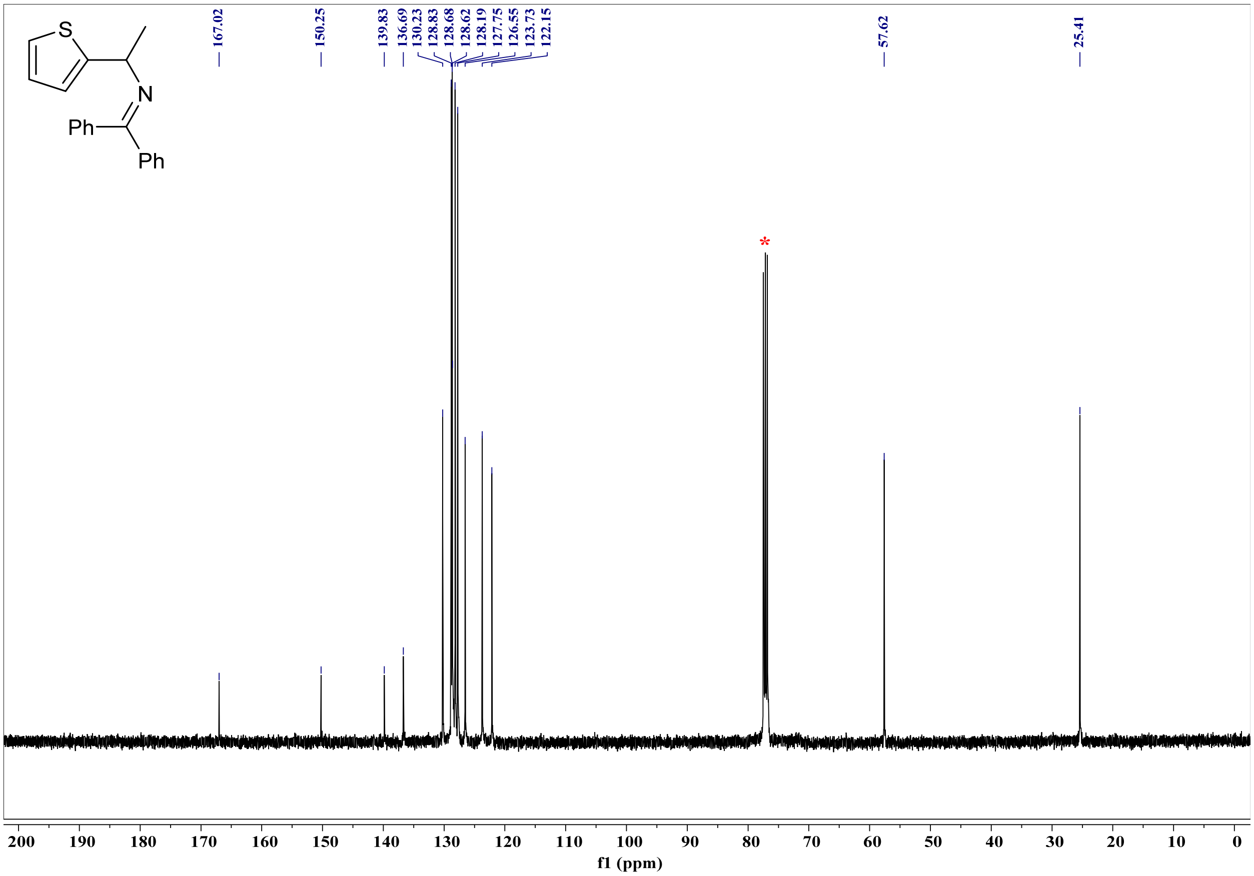

Figure S63. $^{13}C\{^1H\}$ NMR spectrum (100.47 MHz, $CDCl_3$) of 1,1-diphenyl-*N*-(1-(thiophen-2-yl)ethyl)methanimine (**6g**).

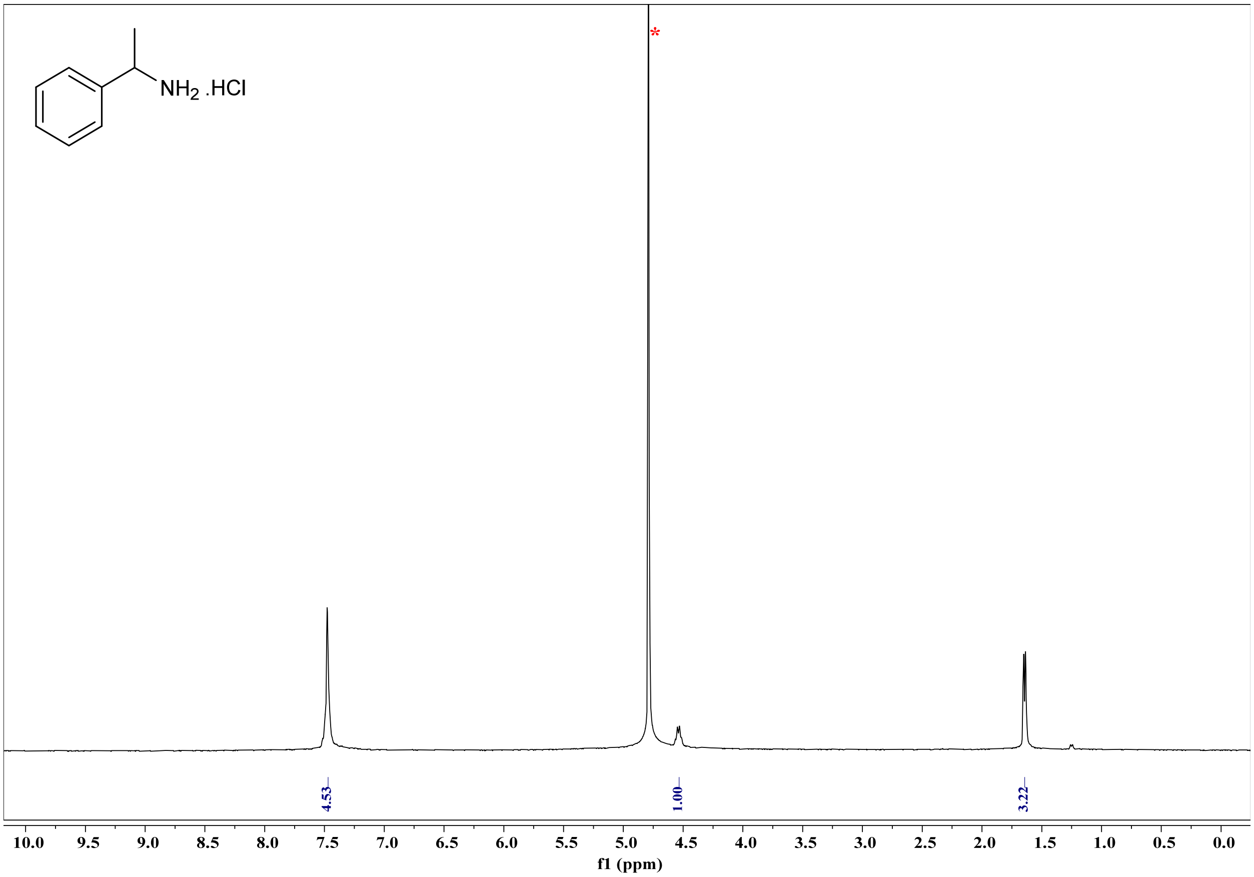

Figure S64. ^{1}H NMR spectrum (400 MHz, D_2O) of 1-phenylethan-1-amine hydrochloride (**6h**).

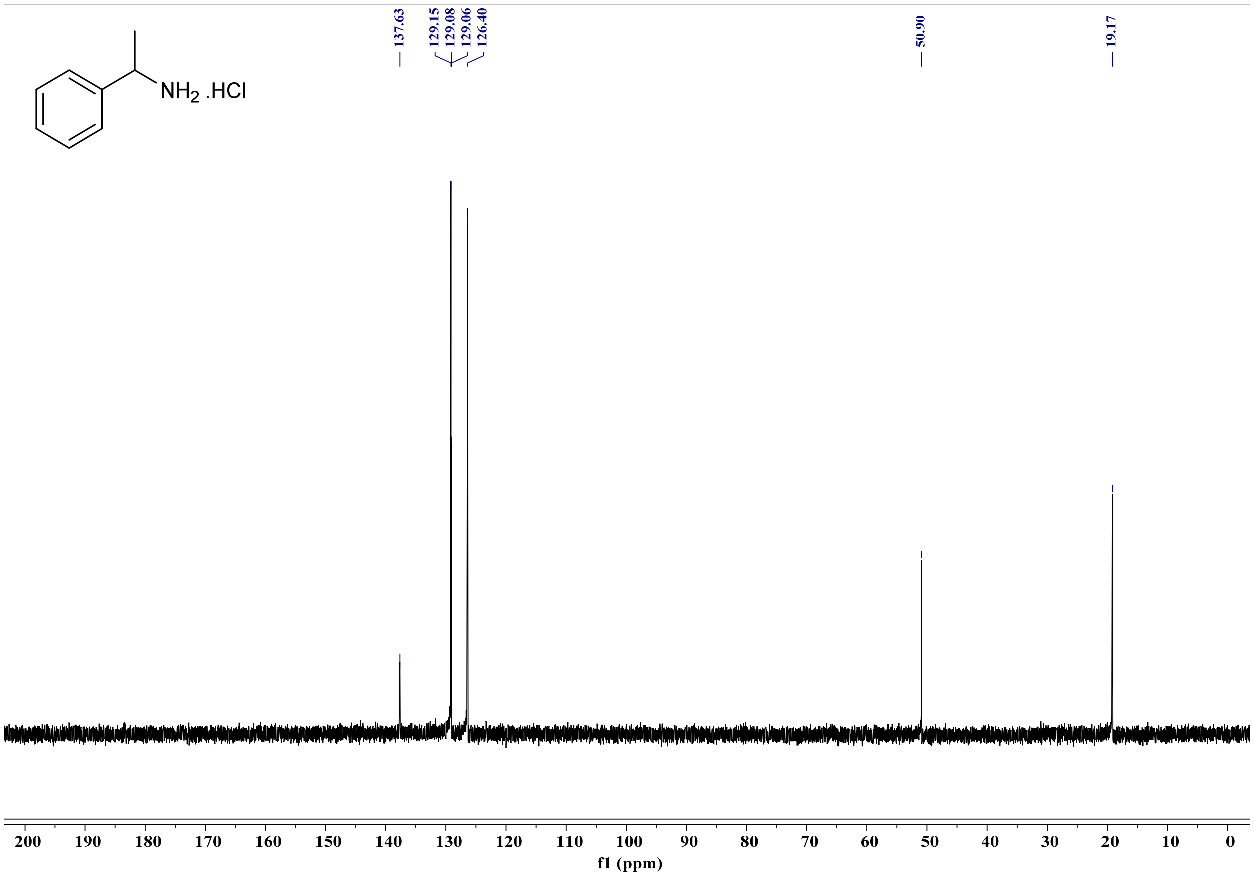

Figure S65. $^{13}C\{^{1}H\}$ NMR spectrum (100.47 MHz, D_2O) of 1-phenylethan-1-amine hydrochloride (**6h**).

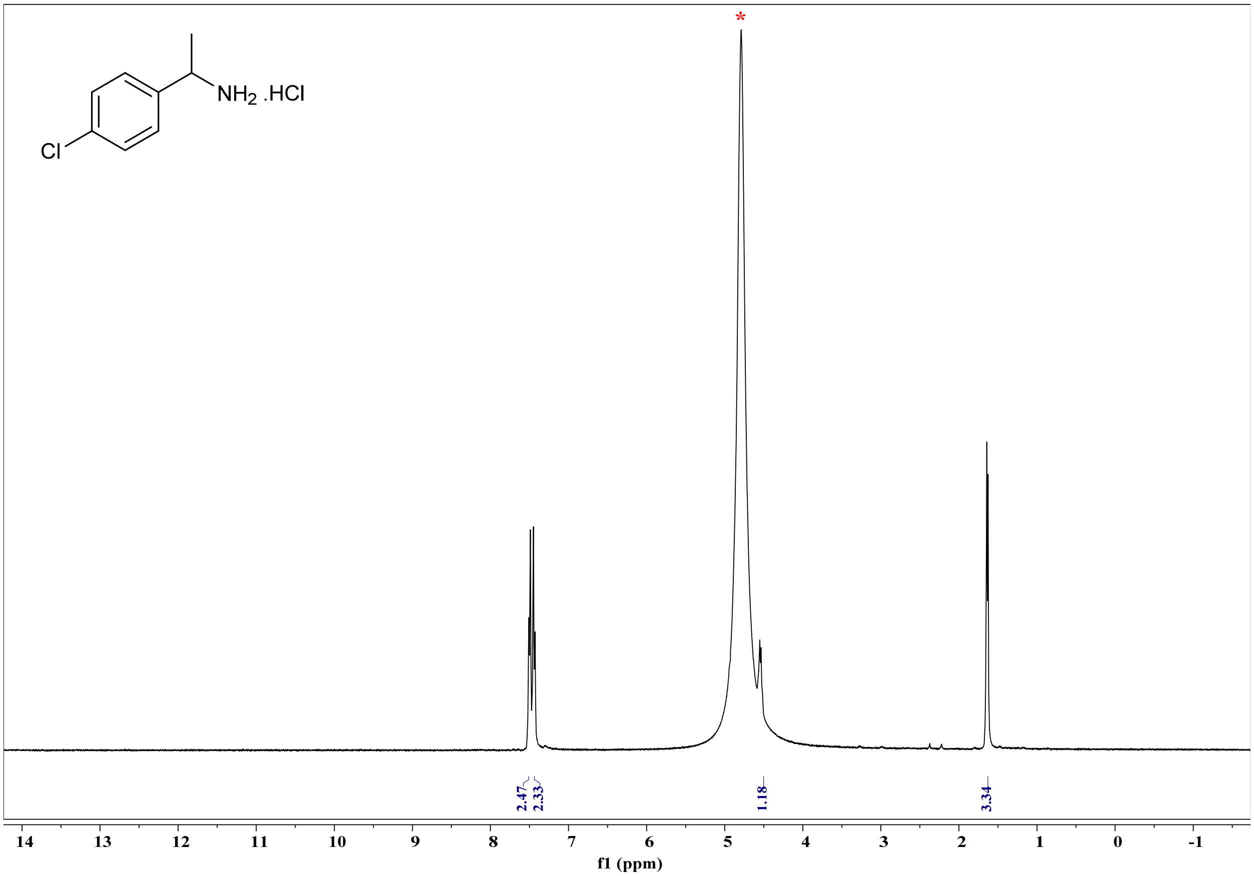

Figure S66. ^{1}H NMR spectrum (400 MHz,D_2O) of 1-(4-chlorophenyl)ethan-1-amine hydrochloride (**6i**).

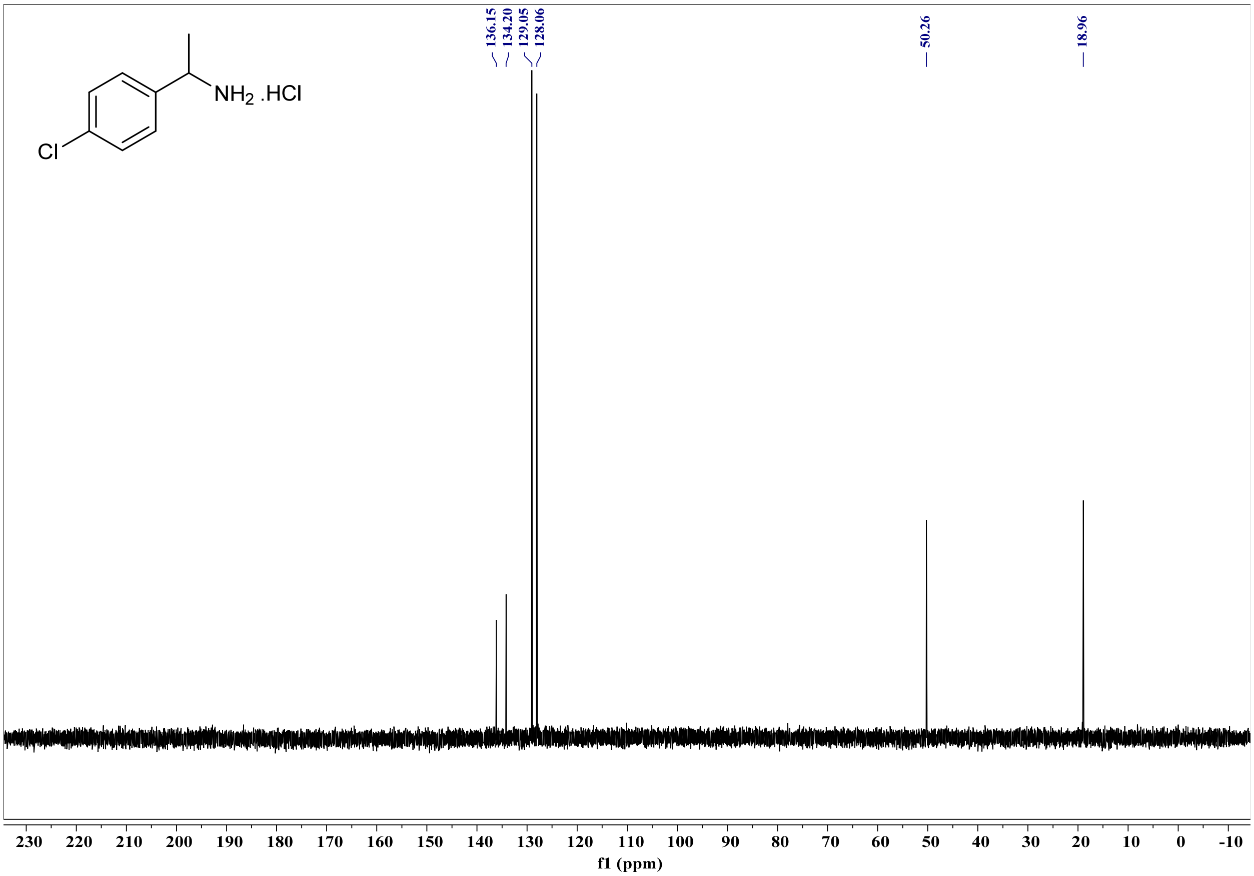

Figure S67. $^{13}C\{^{1}H\}$ NMR spectrum (100.47 MHz, D_2O) of 1-(4-chlorophenyl)ethan-1-amine hydrochloride (**6i**).

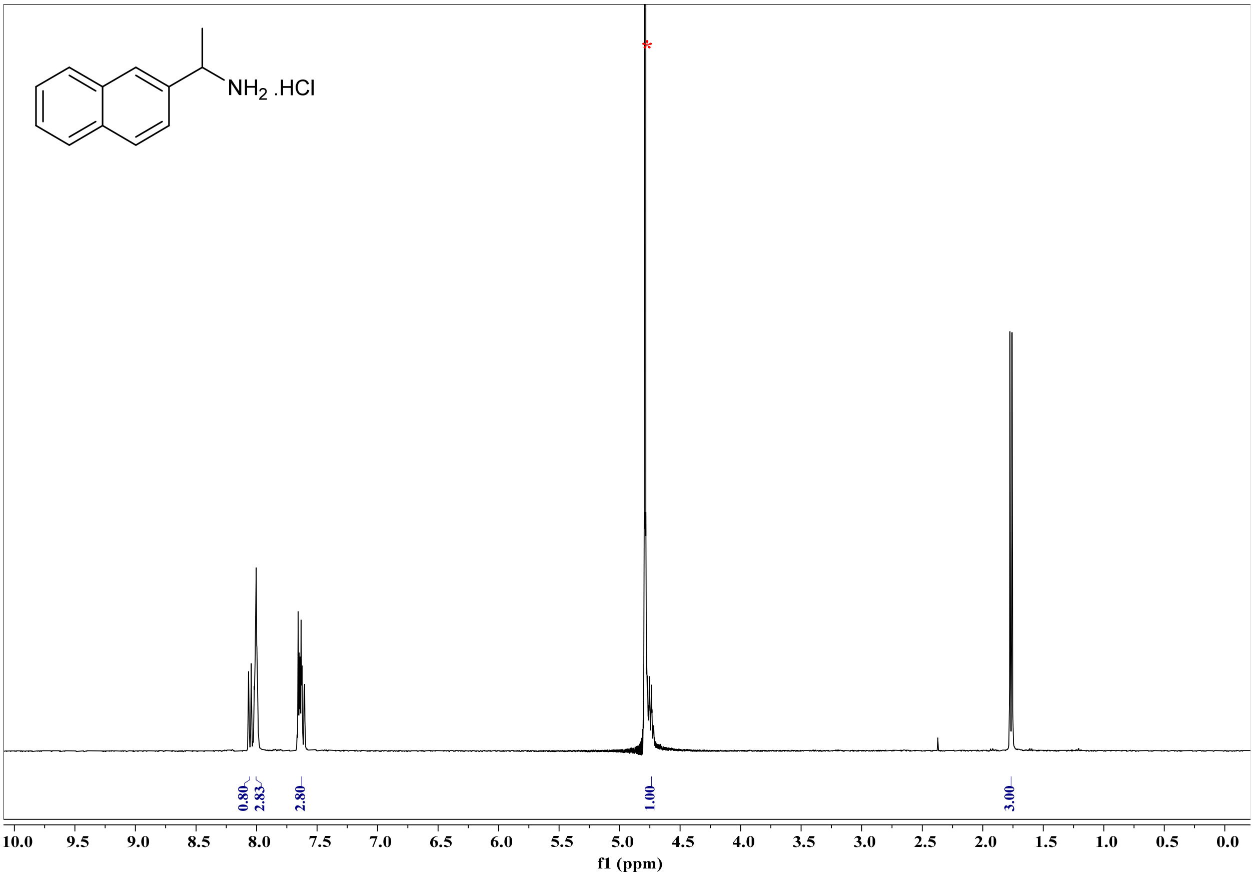

Figure S68. ^{1}H NMR spectrum (400 MHz, D_2O) of 1-(naphthalen-2-yl)ethan-1-amine hydrochloride (**6j**).

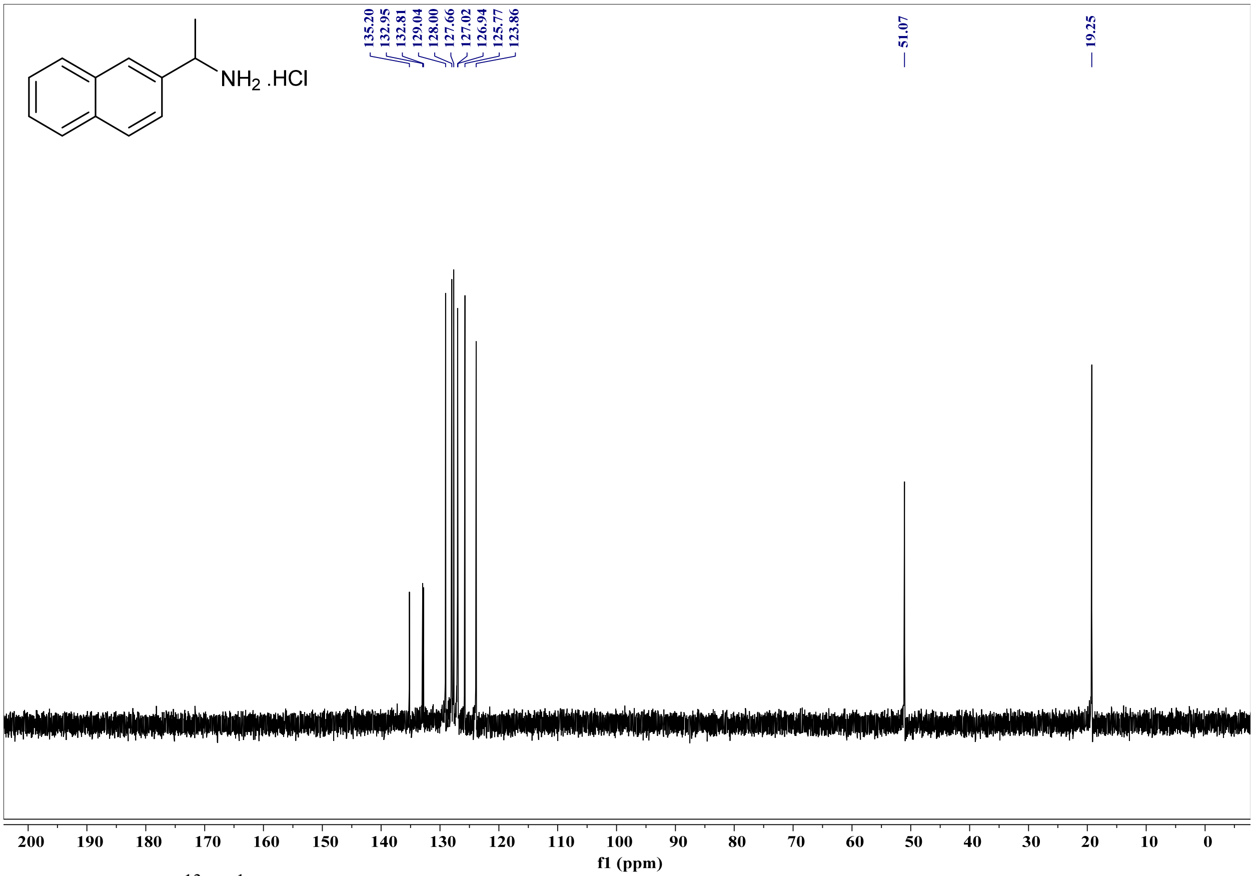

Figure S69. $^{13}C\{^{1}H\}$ NMR spectrum (100.47 MHz, D_2O) of 1-(naphthalen-2-yl)ethan-1-amine hydrochloride (**6j**).

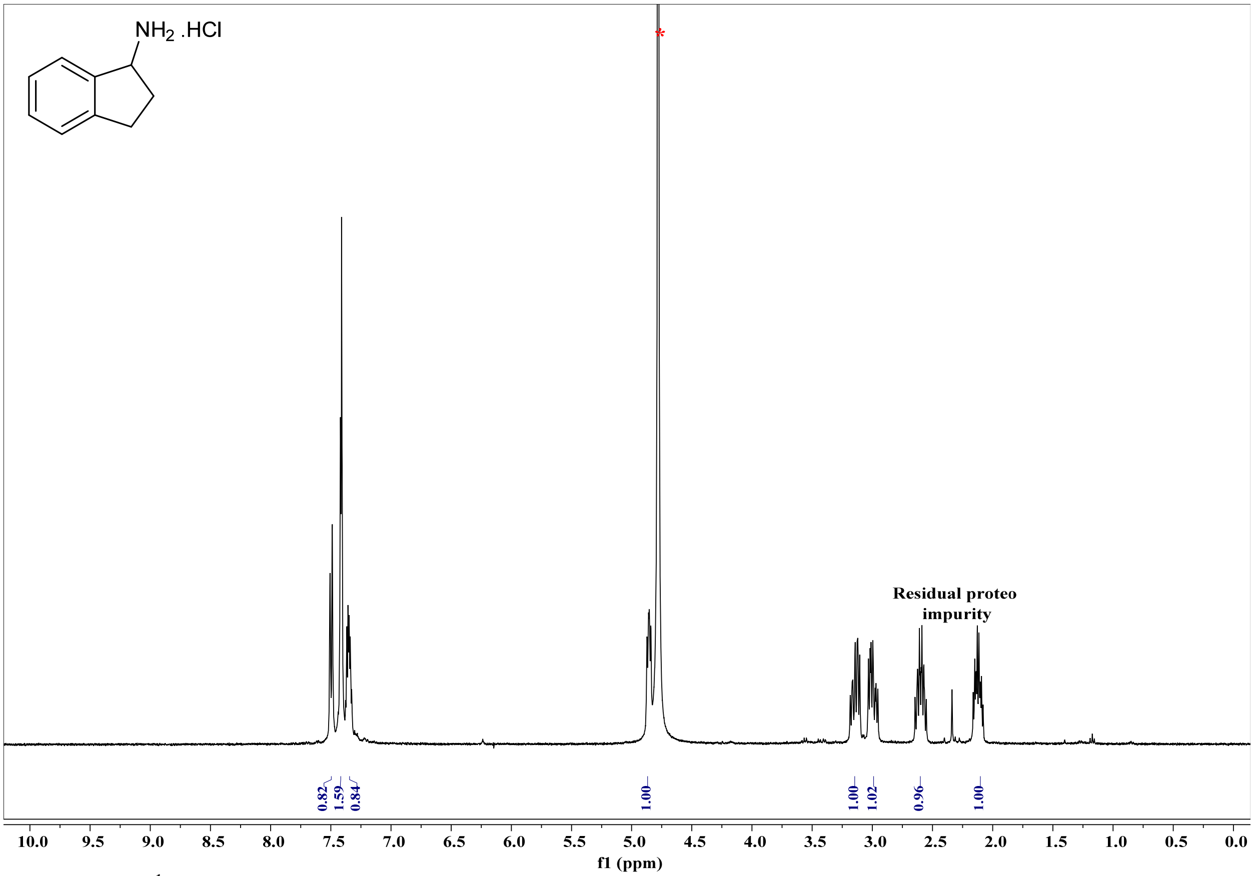

Figure S70. ^{1}H NMR spectrum (400 MHz,D_2O) of 2,3-dihydroinden-1-amine hydrochloride (**6k**).

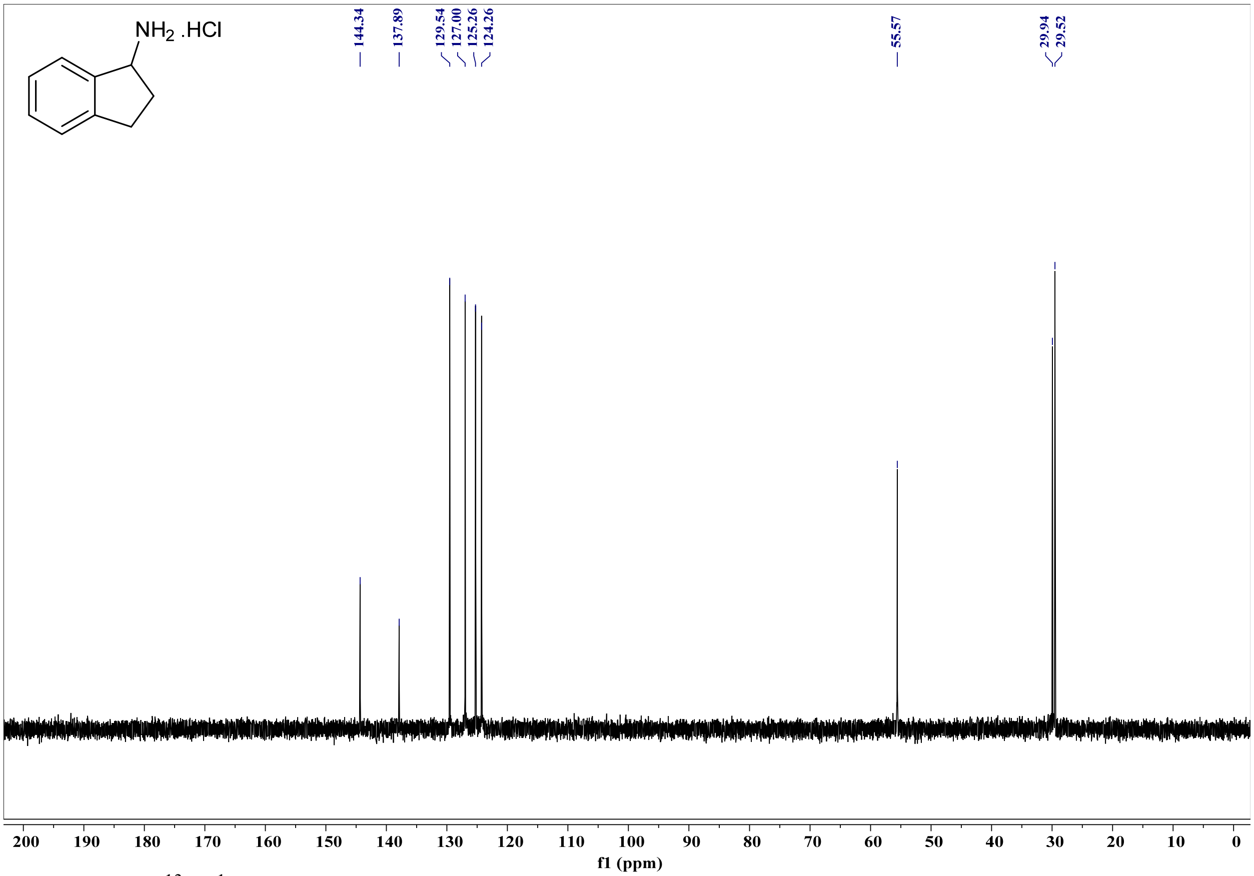

Figure S71. $^{13}C\{^1H\}$ NMR spectrum (100.47 MHz, D_2O) of 2,3-dihydroinden-1-amine hydrochloride (**6k**).

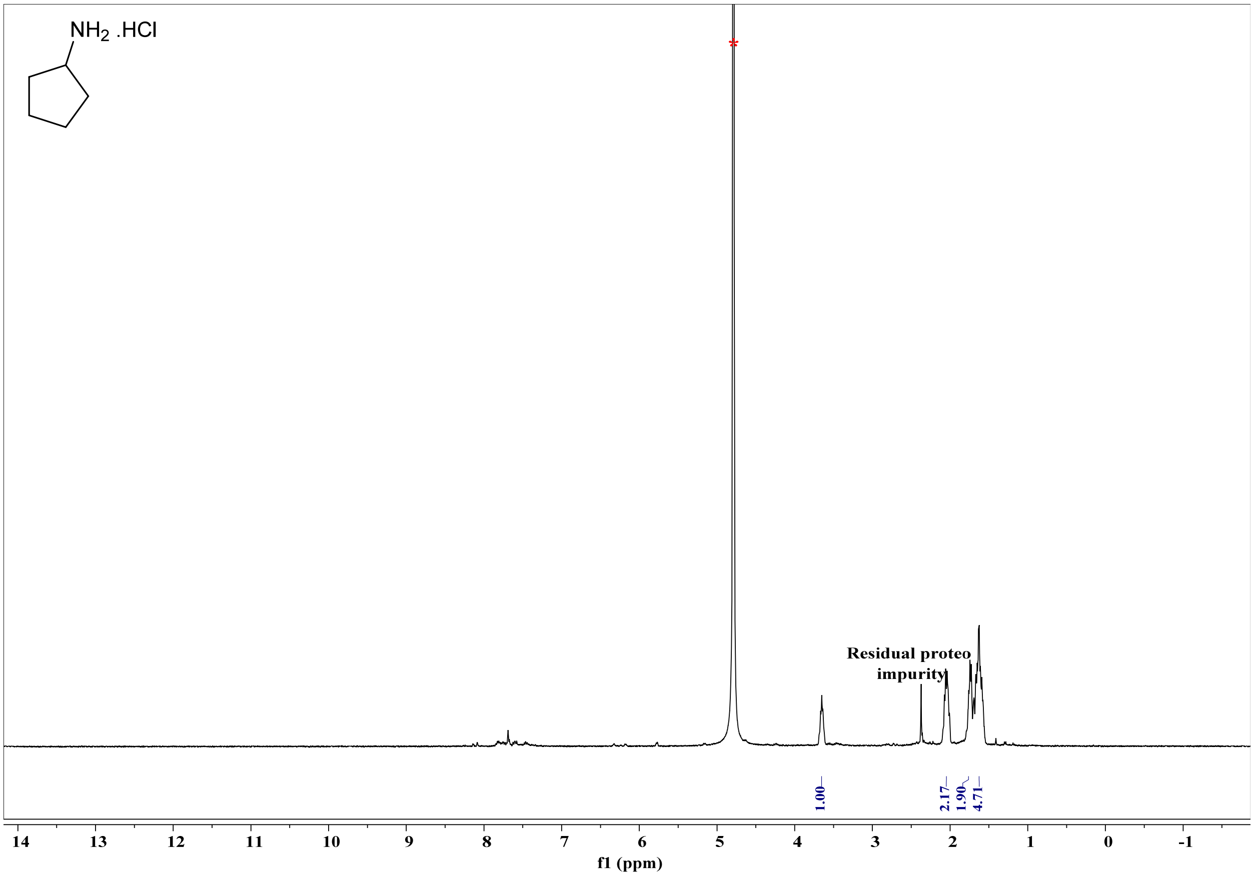

Figure S72. ^{1}H NMR spectrum (400 MHz,D_2O) of cyclopentylamine hydrochloride (**6l**).

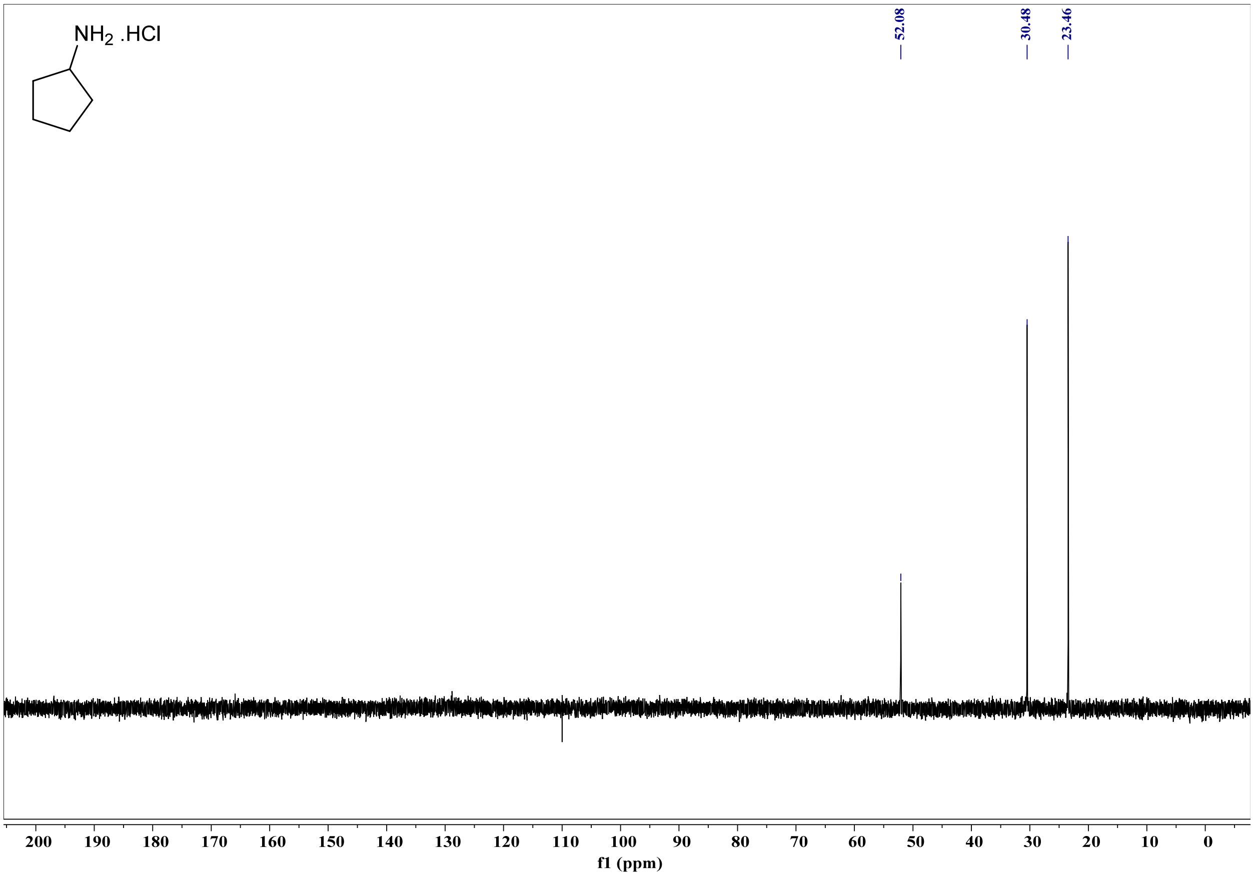

Figure S73. $^{13}C\{^1H\}$ NMR spectrum (100.47 MHz, D_2O) of cyclopentylamine hydrochloride (**6l**).

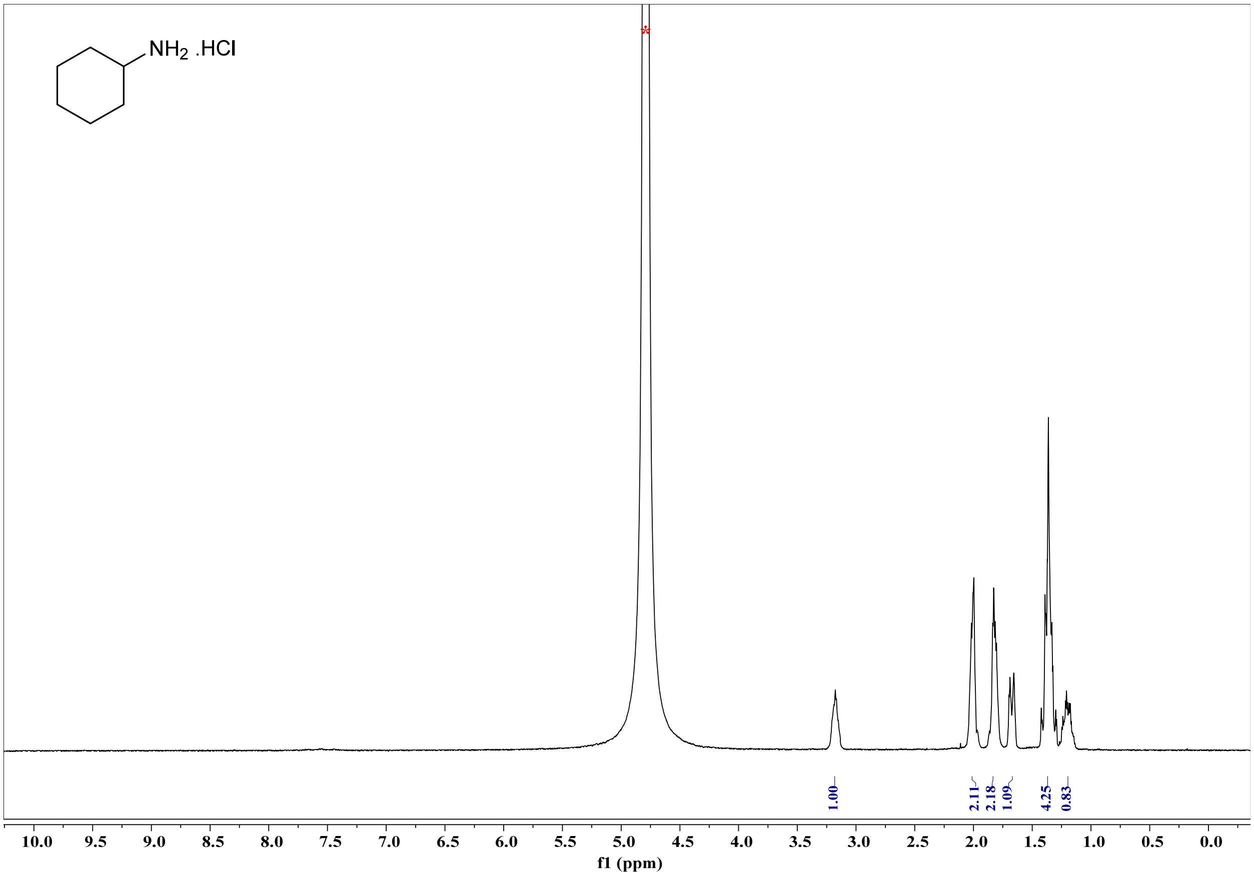

Figure S74. ^{1}H NMR spectrum (400 MHz,D_2O) of cyclohexylamine hydrochloride (**6m**).

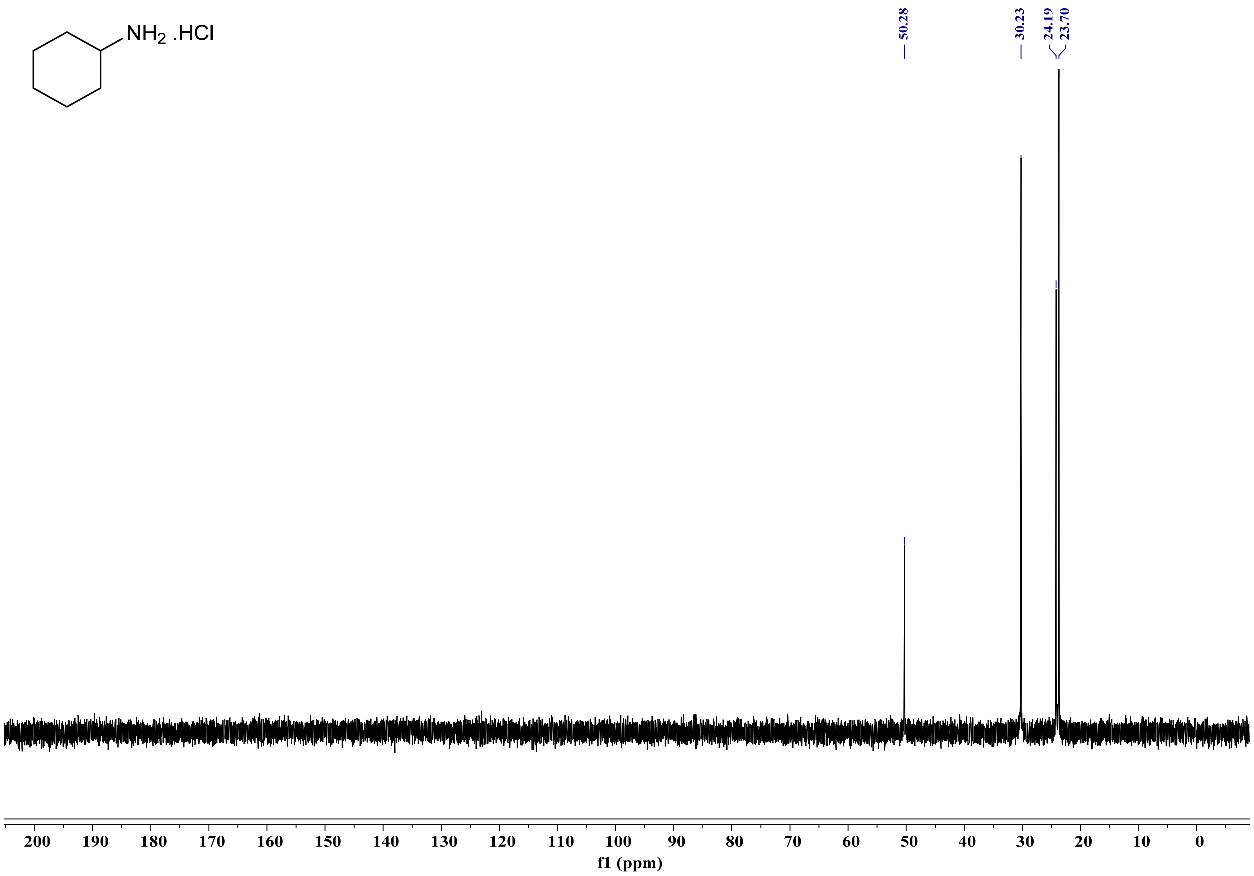

Figure S75. $^{13}C\{^1H\}$ NMR spectrum (100.47 MHz, D_2O) of cyclohexylamine hydrochloride (**6m**).

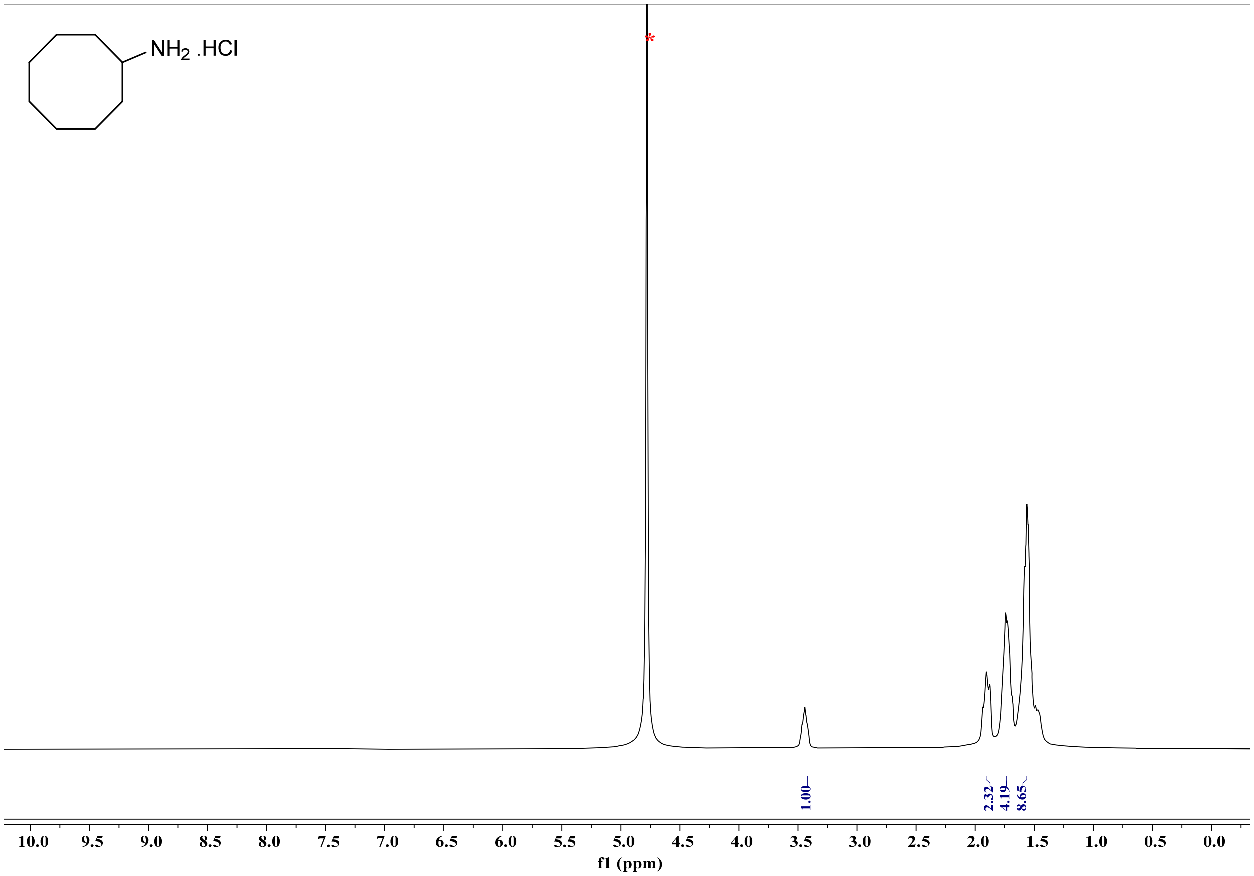

Figure S76. ^{1}H NMR spectrum (400 MHz,D_2O) of cyclooctylamine hydrochloride (**6n**).

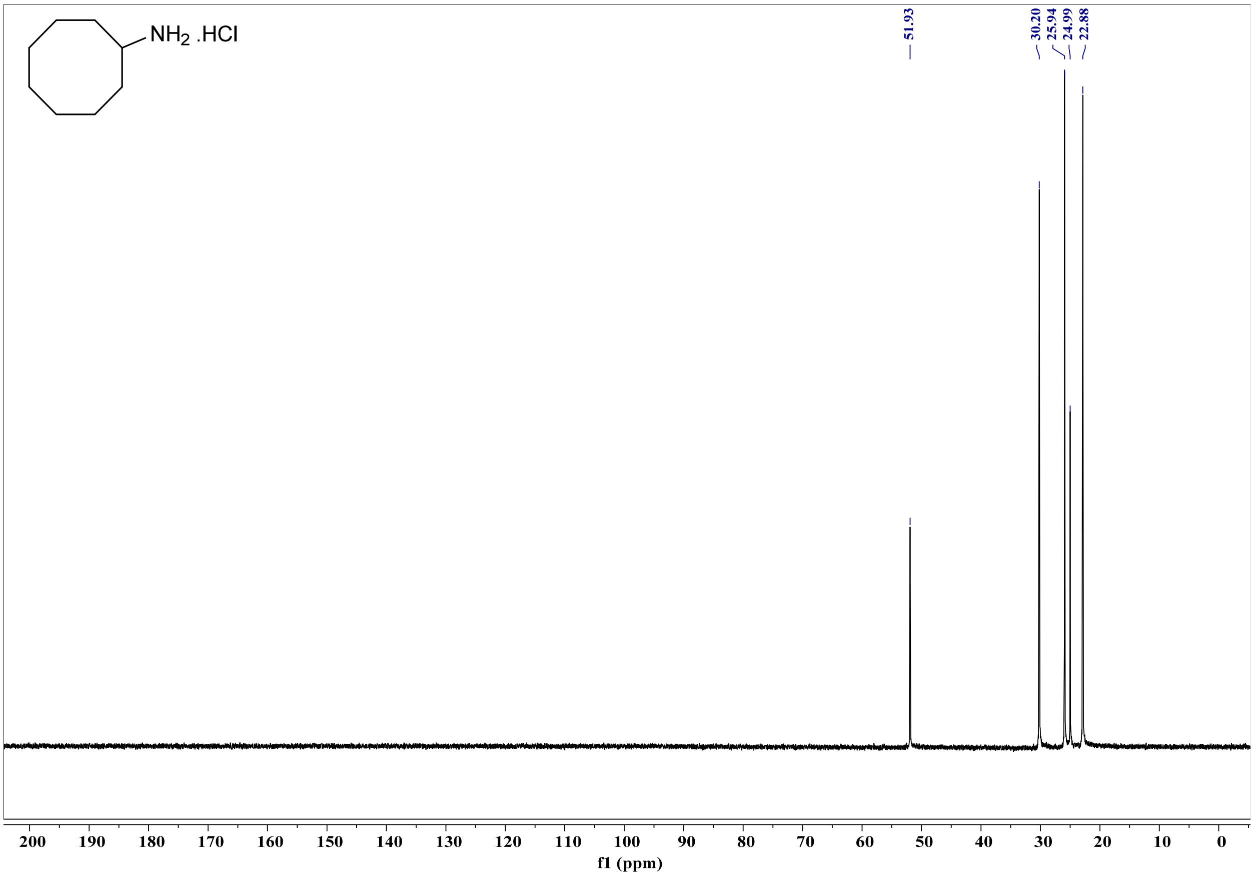

Figure S77. $^{13}C\{^1H\}$ NMR spectrum (100.47 MHz, D_2O) of cyclooctylamine hydrochloride (**6n**).

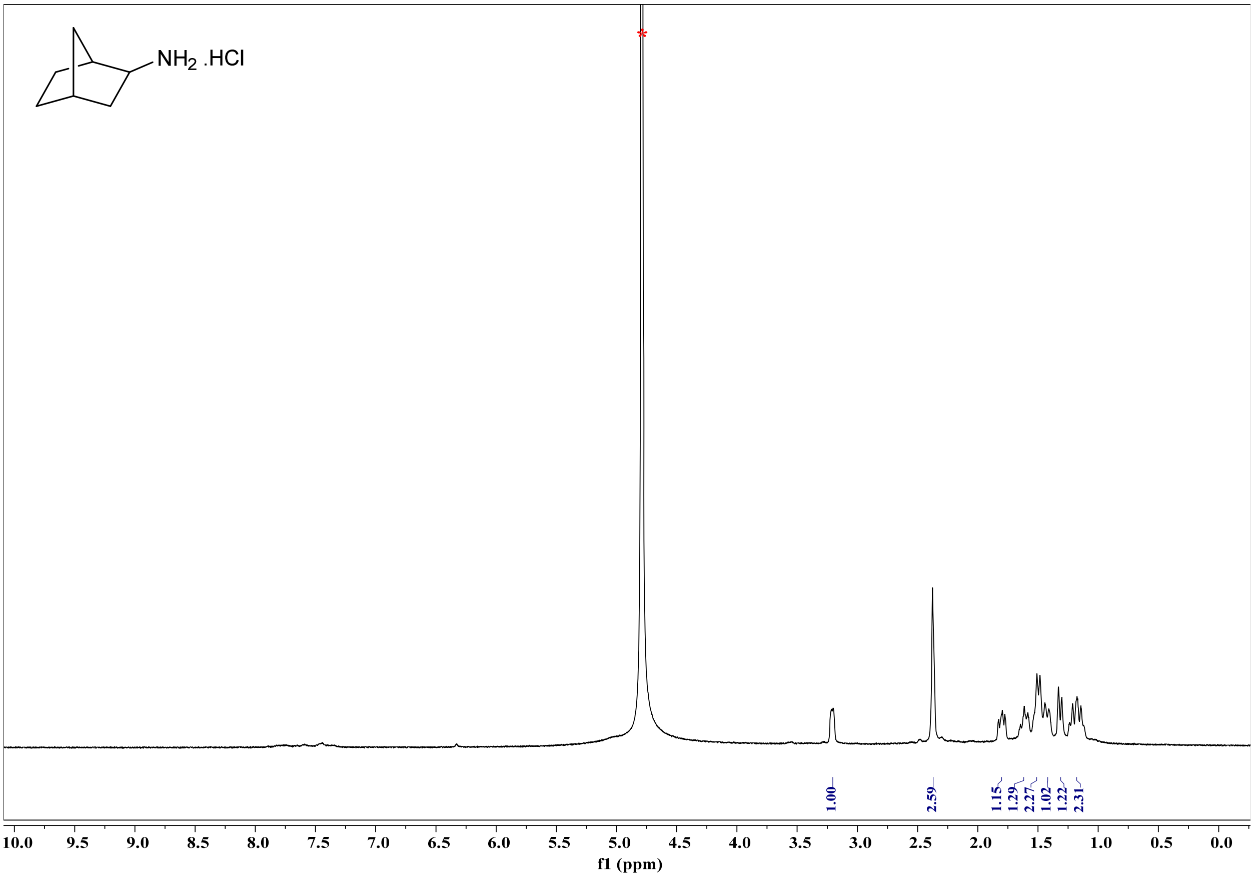

Figure S78. ^{1}H NMR spectrum (400 MHz, D_2O) of 2-aminonorbornane hydrochloride (**6o**).

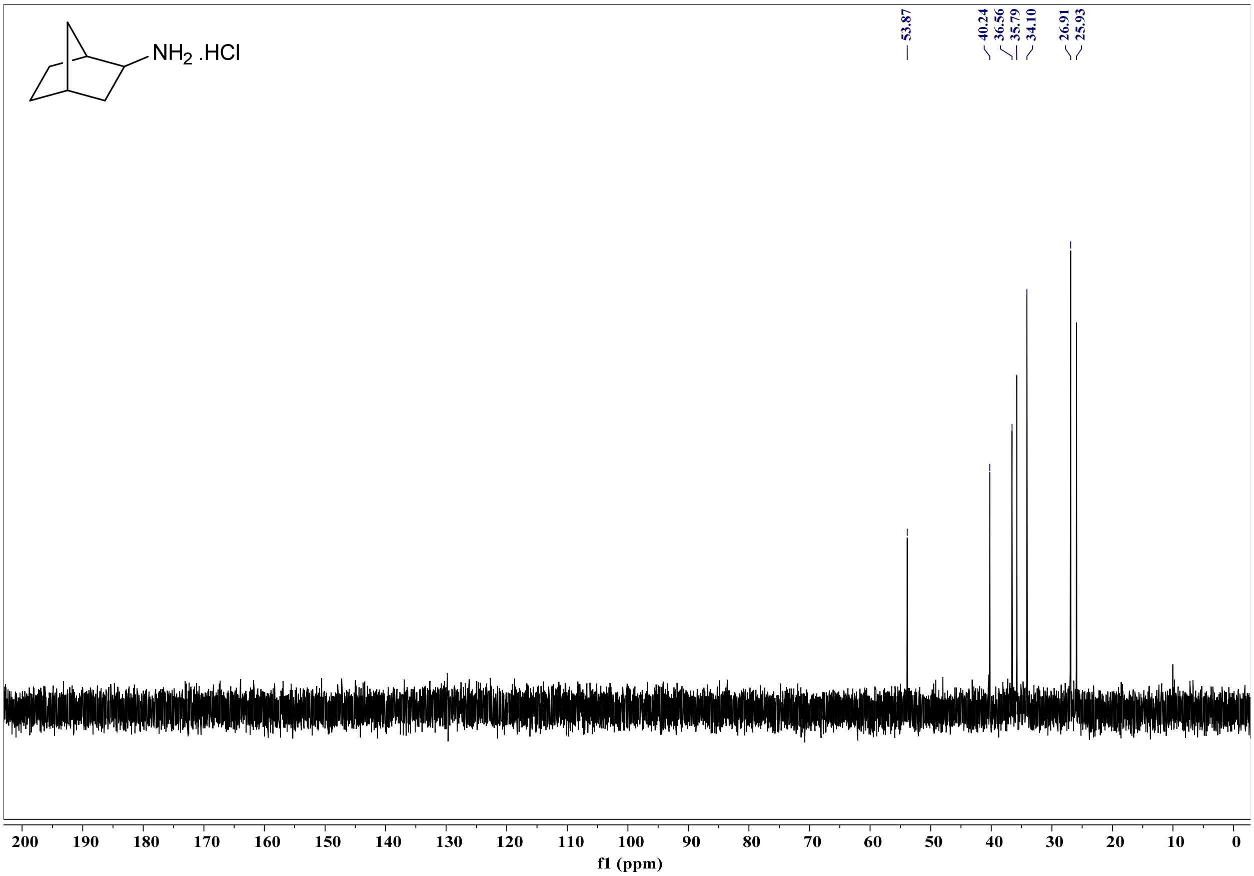

Figure S79. $^{13}C\{^1H\}$ spectrum (100.47 MHz, D_2O) of 2-aminonorbornane hydrochloride (**6o**).
